# Comparison of comprehensive quantitative EEG metrics between typically developing boys and girls in resting state eyes-open and eyes-closed conditions

**DOI:** 10.3389/fnhum.2023.1237651

**Published:** 2023-11-06

**Authors:** Mo Modarres, David Cochran, David N. Kennedy, Jean A. Frazier

**Affiliations:** ^1^The Eunice Kennedy Shriver Center, Department of Psychiatry, University of Massachusetts Chan Medical School, Worcester, MA, United States; ^2^The Eunice Kennedy Shriver Center, Department of Psychiatry, University of Massachusetts Chan Medical School/UMass Memorial Health Care, Worcester, MA, United States

**Keywords:** quantitative EEG (qEEG), spectral analysis, coherence, functional connectivity, resting state

## Abstract

**Introduction:**

A majority of published studies comparing quantitative EEG (qEEG) in typically developing (TD) children and children with neurodevelopmental or psychiatric disorders have used a control group (e.g., TD children) that combines boys and girls. This suggests a widespread supposition that typically developing boys and girls have similar brain activity at all locations and frequencies, allowing the data from TD boys and girls to be aggregated in a single group.

**Methods:**

In this study, we have rigorously challenged this assumption by performing a comprehensive qEEG analysis on EEG recoding of TD boys (*n* = 84) and girls (*n* = 62), during resting state eyes-open and eyes-closed conditions (EEG recordings from Child Mind Institute’s Healthy Brain Network (HBN) initiative). Our qEEG analysis was performed over narrow-band frequencies (e.g., separating low *α* from high *α*, etc.), included sex, age, and head size as covariates in the analysis, and encompassed computation of a wide range of qEEG metrics that included both absolute and relative spectral power levels, regional hemispheric asymmetry, and inter- and intra-hemispheric magnitude coherences as well as phase coherency among cortical regions. We have also introduced a novel compact yet comprehensive visual presentation of the results that allows comparison of the qEEG metrics of boys and girls for the entire EEG locations, pairs, and frequencies in a single graph.

**Results:**

Our results show there are wide-spread EEG locations and frequencies where TD boys and girls exhibit differences in their absolute and relative spectral powers, hemispheric power asymmetry, and magnitude coherence and phase synchrony.

**Discussion:**

These findings strongly support the necessity of including sex, age, and head size as covariates in the analysis of qEEG of children, and argue against combining data from boys and girls. Our analysis also supports the utility of narrow-band frequencies, e.g., dividing *α*, *β*, and *γ* band into finer sub-scales. The results of this study can serve as a comprehensive normative qEEG database for resting state studies in children containing both eyes open and eyes closed paradigms.

## Introduction

A majority of published studies comparing qEEG in typically developing children and children with neurodevelopmental or psychiatric disorders during resting state have focused on a subset of qEEG metrics. These include absolute and relative spectral powers, asymmetry of spectral powers between the right and left hemisphere, and intra- and inter-hemispheric magnitude coherence and phase synchrony. Furthermore, most previous studies have reported a limited number of EEG locations and frequency bands, and have been limited to either eyes open or eyes closed conditions. Most importantly, the majority of reported studies have used a control group (e.g., typically developing children, TD) that combines boys and girls. For example, a review (Newson and Thiagarajan, 2019) of spectral power in psychiatric disorders reports that a majority of studies on children with attention deficit hyperactivity disorder (ADHD) over the last 30 years have combined data from boys and girls in their control group, with a median proportion of girls at 31% (Kuperman et al., 1996; Clarke et al., 1998, 2002a,b; Hermens et al., 2005a,b,c; Clarke et al., 2008; Fonseca et al., 2008; Barry et al., 2009; Barry et al., 2010; Ogrim et al., 2012; Shi et al., 2012; Dupuy et al., 2013; Fonseca et al., 2013; Liechti et al., 2013; Buyck and Wiersema, 2014a,b, 2015; Kitsune et al., 2015; Kamida et al., 2016; Kim et al., 2016; Thomas and Viljoen, 2016; Giertuga et al., 2017; Rommel et al., 2017; Jarrett et al., 2020). Additionally, the majority of studies of children with autism spectrum disorder (ASD) have combined boys and girls in their controls and ASD groups with a median of 27% girls (Dawson et al., 1995; Sutton et al., 2005; Chan and Leung, 2006; Chan et al., 2007; Coben et al., 2008; Sheikhani et al., 2012; Poil et al., 2014; Machado et al., 2015; van Diessen et al., 2015; Jaime et al., 2016; Kozhushko et al., 2018; Lefebvre et al., 2018). Finally, most studies evaluating spectral power asymmetry, spectral ratio of *θ*/*β*, and coherences have also included both boys and girls in their control groups with the controls’ sample size ranging from 12–554, and percent girls ranging from 12%–40% (Barry et al., 2005, 2011; Duffy and Als, 2012; Carson et al., 2014; Han and Chan, 2017; Lauttia et al., 2019; Kang et al., 2021).

This typical presentation of data suggests a widespread supposition that typically developing boys and girls have similar brain activity at all locations and frequencies, allowing the data from TD boys and girls to be aggregated in a single group. Despite this implicit biological sex homogeneity assumption, there have been a number of studies in the past 30 years reporting differences in qEEG metrics of TD boys and girls in children. An earlier study by Marosi et al. (1993) reported a sex difference in EEG magnitude coherence of typically developing children (*n* = 18 girls and 24 boys). Barry et al. (2004) compared magnitude coherence in TD boys and girls, assessed during an eyes-closed resting condition, as a function of sex and age. They reported a difference of coherences in boys and girls across several regions and in the alpha frequency band. Nentwich et al. (2020) reported sex and age differences in EEG based functional connectivity, computed from measures of phase synchrony, using the same database as this study that included both TD children as well as children with a variety of psychiatric disorders.

A recent study (Isler et al., 2023) in children (4–11 years of age) has reported significant EEG spectral power differences in the eyes-open (EO) versus the eyes-closed (EC) conditions across spectral frequencies for all ages, where girls showed more prominent decrease in EO power (vs. EC) at 8 Hz (low alpha frequency) compared to boys.

We have used high-density EEG recordings, from a publicly available biobank that was created by the Child Mind Institute’s Healthy Brain Network (HBN) initiative and have performed a comprehensive qEEG analysis on TD boys (*n* = 84) and girls (*n* = 62) during resting state EO and EC conditions. Our qEEG analysis is performed over narrow-band frequencies (e.g., separating low *α* from high *α*, etc.), includes sex, age, and head size, as covariates in the analysis, and encompasses computation of a wide range of qEEG metrics that include both absolute and relative spectral power levels, regional hemispheric asymmetry (absolute and relative spectral powers), and inter- and intra-hemispheric magnitude coherence and phase synchrony among cortical regions. The goal of this comprehensive analysis is to determine whether sex differences in qEEG metrics warrant separation of analyses by sex, and to determine the breadth of dataset features that differ by sex in TD children and adolescents.

## Materials and methods

The source of data was EEG recordings from a publicly available biobank that was created by the Child Mind Institute’s Healthy Brain Network (HBN) initiative (Child Mind Institute, 2017). The biobank, obtained from children and adolescents (ages 5–21 years) residing in the New York City area, includes psychiatric, behavioral and cognitive phenotyping, as well as multimodal brain imaging, electroencephalography (EEG), eye tracking, genetics, digital voice and video samples, and actigraphy. Alexander et al. (2017) describes the structure of the databank, EEG, data acquisition, test procedures and paradigms, which we briefly summarize here.

The EEG signals of the biobank were obtained using a 128-channel EEG geodesic hydrocel system by Electrical Geodesics Inc. (EGI). High-density EEG data were recorded in a sound-shielded room at a sampling rate of 500 Hz with a bandpass of 0.1 to 100 Hz. Recorded EEG data were preprocessed at HBN in MATLAB (MathWorks, Natick, MA, United States) and EEGlab 13.3.2.b package according to the methods described in Langer et al. (2017). Briefly, preprocessing included identifying and replacing bad EEG electrodes using spherical spline interpolation. The EEG data were then high-pass filtered at 0.1 and notch filtered (59–61 Hz) with a Hamming windowed-sinc finite impulse response zero-phase filter. Next, sparse noise from the data were removed using principal components analysis (PCA). At the last stage of preprocessing the entire dataset for each subject was visually inspected and any segment that remained noisy after the automatic and manual noise removal methods were discarded. The resting paradigm consisted of EEG acquisition while the participant viewed a standard fixation cross in the center of the computer screen and a recorded voice of a female research assistant instructed them to “now open your eyes” (rest with eyes open for 20 s) and “now close your eyes” (rest with eyes closed for 40 s); this procedure was repeated 5 times, alternating between eyes opened and eyes closed.

From the entire database, we identified a group of children between 5 and 16 years of age, *n* = 84 boys (median age = 8.6), and *n* = 62 girls (based on biologic sex at birth, median age = 9.2), who had no diagnoses of ADHD, ASD, intellectual disability (ID), learning disorder, or psychiatric illness, and had usable EEG recordings for all 5 segments of eyes open and eyes closed paradigms. We refer to this cohort as “typically developing” (TD) in this manuscript.

### Data analysis

**Figure 1:**
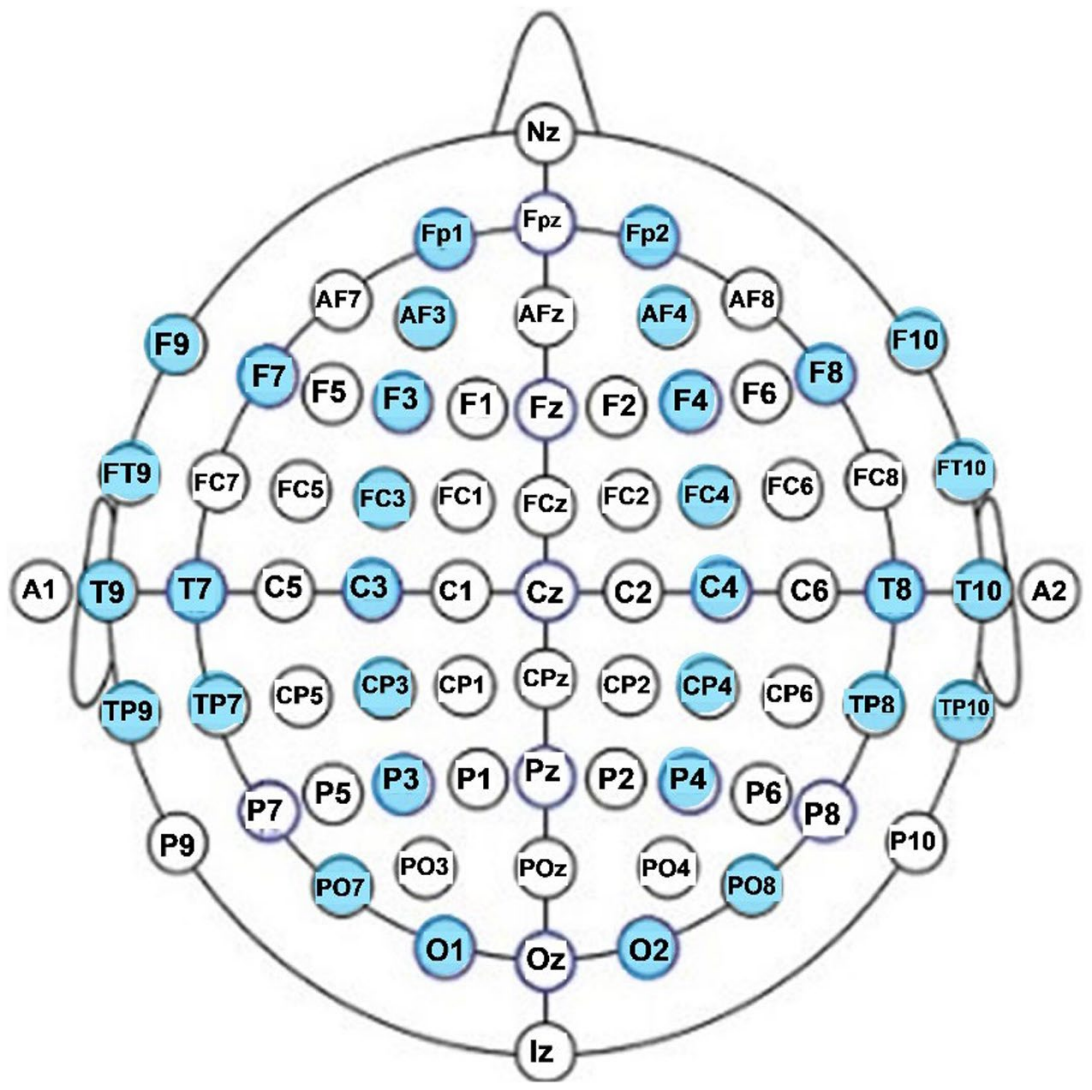
EEG electrode locations analyzed in this study (in blue).

For each participant, EEG data were analyzed and averaged for each of the two resting conditions (eyes open, eyes closed). To reduce overestimation bias of electrodes that are located in close proximity to each other, while keeping representation from all cortical lobes and areas of the two hemispheres, we focused our analysis on 31 EEG electrode sites: pre-frontal sites fp1 and fp2 anterior frontal sites AF3 and AF4; frontal sites F3, F4, F7, F8, F9; fronto-central sites FC3 and FC4; fronto-temporal sites Ft9 and Ft10; temporal sites T7, T8, T9, T10; central sites C3 and C4; centro-parietal sites CP3 and CP4; temporal-parietal sites TP7, TP8, TP9, TP10; parietal P3 and P4; parieto-occipital sites PO7and PO8, and occipital sites O1 and O2. The 32 EEG locations that were analyzed are shown in the diagram of Figure 1.

Data analysis consisted of 2 parts: (1) power spectral analysis on each of the 31 channels of EEG, and (2) pair-wise coherence analysis, consisting of computing the magnitude coherence and phase synchrony of 496 unique pairs of EEG channel recordings $\left(\overset{31}{\underset{n=1}{\sum }}n=496\right)\text{.}$

#### Spectral analysis

Using a custom script in MATLAB, we computed both absolute and relative powers of 4 s sliding windows which were overlapped by 1 s, and which produced a time-varying power spectra with a 0.25 Hz resolution (1/4 s). Relative spectra were computed by dividing the absolute power spectra of each 4 s segment by the total spectral power of that segment. We computed the mean and standard deviation (s.d.) of these power spectra over the entire eyes open or eyes closed segments, and over the specific narrow-band frequencies shown in Table 1.


(1) $$CF_{e_{i}\;e_{j}}\left(f\right)=\frac{{\left|G_{e_{i}\;e_{j}}\left(f\right)\right|}^{2}}{G_{e_{i}\;e_{i}x}\left(f\right)G_{e_{j}\;e_{j}}\left(f\right)}$$

Magnitude coherence between the EEG signals $e_{i}$ and $e_{j}$ . $G_{e_{i},e_{j}}\left(f\right)$ is the cross-spectral density between e_i_ and *e_j_* at the frequency *f*. $G_{e_{i},e_{i}}\left(f\right)$ and $G_{e_{j},e_{j}}\left(f\right)$ are the auto-spectral density (at frequency *f*) of *e_i_* and *e_j_*, respectively.


(2) $$\begin{array}{l} \mathrm{Local}\\ BCM\;\left(e_{i},e_{j},F_{\mathrm{range}},T_{\mathrm{period}}\right)=\\ \frac{1}{\mathrm{length}\left(T_{\mathrm{period}}\right)\mathrm{length}{\left(T_{\mathrm{range}}\right)}_{t=T}}\underset{t=T_{\mathrm{period}}}{\sum }\underset{f=F_{\mathrm{range}}}{\sum }C_{e_{i},e_{j}}\left(t,f\right) \end{array}$$

(3) $$\begin{array}{l} \mathrm{Regional}\\ BCM_{\mathrm{Region}}\left(F_{\mathrm{range}},T_{\mathrm{period},}\;\mathrm{Test}\;\mathrm{Condition}\right)\\ \;=\frac{1}{\mathrm{length}\left(e_{i},e_{j}\in \mathrm{Brain}\;\mathrm{Region}\right)}\;\underset{\;e_{i},e_{j}\in \mathrm{Brain}\;\mathrm{Region}}{\sum }\\ \;BCM\;\left(e_{i},e_{j},F_{\mathrm{range}},T_{\mathrm{period},}\;\mathrm{Test}\;\mathrm{Condition}\right)\; \end{array}$$

(4) $$\begin{array}{l} \mathrm{Global}\\ BCM_{\mathrm{Global}}=\\ \;f\;\left(w_{1}.BCM_{\mathrm{Region}\;1,}\;w_{2}.BCM_{\mathrm{Region}\;2,\ldots ,}\;w_{n}.BCM_{\mathrm{Region}\;n}\right) \end{array}$$

**Table 1:** Spectral frequency bands used in our analysis.

Frequency bands (Hz)	1–3.75	4–7.75	8–9.75	10–11.75	12–14.75	15–19.75	20–24.75	25–29.75	30–39.75	40–49.75	65–69.75	70–100
Symbol	*δ*	*θ*	*α*1	*α*2	*β*1	*β*2	*β*3	*β*4	*γ*1	*γ*2	*γ*3	*γ*4

#### Magnitude coherence

We utilized a hierarchical EEG coherence analysis method that has been developed and published by our group (Modarres et al., 2021), which allows for formal inclusion of analysis duration, EEG frequency band, cortical region, and experimental test condition in the computation of the EEG magnitude coherences.

The structure of the analysis, which is referred to as Brain Coherence Marker (BCM), is depicted in Eq. 2, where $C_{e_{i},e_{j}}\left(t,f\right)$ is the magnitude coherence between the EEG signals $e_{i}$ and $e_{j}$ from two different scalp electrode locations at frequency *f* (Eq. 1) and at time *t*. The summations of Eq. 2 indicate that these instantaneous coherences are integrated over a specific time period *T*_period_ and frequency range $F_{\mathrm{range}}$ .

Eq. 3 shows an expansion of the coherence marker where the BCMs are computed for a specified brain region (BCM_Region_) during a given test paradigm, and Eq. 4 is a further expansion that combines BCM_Region_ to produce a global BCM (BCM_Global_) reflecting the overall coherences across the entire brain. The time periods of BCM were selected to span the duration of eyes open and eyes closed periods. The computed BCMs of 5 repetitions of eyes closed (EC) and eyes open (EO) conditions were combined separately, and the frequency bands were similar to those shown in Table 1.

### Hemispheric and regional-based grouping of EEG channels and pairs

To reduce complexity and increase computational efficiency, we used the BCM_Region_ structure (Eq. 3) where coherence pairs were grouped across frontal, temporal, parietal, occipital, and central lobes and regions of both hemispheres, as shown in Table 2. The output of this computation was 36 “regional pairs” that consisted of left hemispheric, right hemispheric, and interhemispheric coherent pairs shown in Table 3.

**Table 2:** Grouping of EEG channels for each region of analysis.

Left hemisphere	Right hemisphere
Left frontal (LF) fp1, AF3, F3, F7, F9 Left central (LC) FC3, C3, CP3 Left temporal (LT) Ft9, T7, Tp7, TP9 Left parietal/occipital (LP/O) P3, PO7, O1	I. Right frontal (RF) fp2, AF4, F4, F8 Right central (RC) FC4, C4, CP4 Right temporal (RT) Ft10 T8 TP8 TP10 Right parietal/occipital (RP/O) P4 PO8 O2

**Table 3:** Regional pairs used in the coherence analysis.

Intra-hemispheric regional pairs			Inter-hemispheric connections between regions in the left (L) and right (R) hemispheres	
	Left hemisphere	Right hemisphere		
Within frontal region	LF–LF	RF-RF	LF–RF	RF–LC
Between frontal and central regions	LF–LC	RF -RC	LF–RC	RF–LT
Between frontal and temporal regions	LF–LT	RF–RT	LF–RT	RF–LP/O
Between frontal and parietal/occipital	LF–LP/O	RF–RP/O	LF–RP/O	RC–LT
Within central region	LC–LC	RC–RC	LC–RC	RC–LP/O
Between central and temporal regions	LC–LT	RC–RT	LC–RT	RT–LP/O
Between central and parietal/occipital	LC–LP/O	RC–RP/O	LC–RP/O	
Within occipital region	LT–LT	RT–RT	LT–RT	
Between temporal and parietal/occipital	LT–LP/O	RT–RP/O	LT–RP/O	
Within parietal/occipital region	LP/O–LP/O	RP/O–RP/O	LP/O–RP/O	

#### Phase synchrony


(5) $$\mathrm{Coherency}=\frac{G_{e_{i},e_{j}}}{\sqrt{G_{e_{i},e_{j}}\left(f\right)G_{e_{i},e_{j}}\left(f\right)}}$$

$G_{e_{i},e_{j}}\left(f\right)$ is the cross-spectral density between *e_i_* and *e_j_* at the frequency *f*. $G_{e_{i},e_{i}}\left(f\right)$ and $G_{e_{j},e_{j}}\left(f\right)$ are the auto-spectral density (at frequency *f*) of *e_i_* and *e_j_*, respectively


$$\mathrm{Phase}\;\mathrm{Synchrony}=\mathrm{Imag}\;\left(\mathrm{Coherency}\right)$$

The magnitude coherence of Eq. 1 can be thought of as amplitude-amplitude coupling of two EEG waveforms. This amplitude coupling, however, can be affected by the neural activity of a single generator within the brain that is observable in the EEG measurement from many scalp locations (volume conduction artifact). In such a case, part of the amplitude coupling is related to the common source affecting the amplitudes of all EEG signals, and hence, not related to the coupling of the two EEG sites under consideration. A solution to the volume conduction artifact is computing a phase-phase coupling between two EEG signals, as there would be minimal or no time-lag between scalp EEG and the underlying common source activity. Nolte et al. (2004) suggested a measure of such phase coupling, referred to as “phase synchrony,” computed according to Eq. 5: we first compute coherency, a complex measure of cross-spectral density that is unweighted by the signal amplitudes/power. The phase synchrony measure is then computed as the imaginary part of this coherency, reflecting stable phase differences between the two EEGs (phase coupling).

### Group comparisons

A custom script in MATLAB was utilized in all of the group comparisons between boys and girls. Since Nentwich et al. (2020) have reported that head size of the children in the HBN database was correlated with their age and sex, we included the head size in our regression analysis. The analysis thus consisted of a 3-factor ANOVA structure, where factor 1: sex (boy or girl), factor 2: participant age, and factor 3: Head Size. The analysis produced *F* and *p*-values for each of the factors. We used a significant *p*-value of 0.05 after performing a multiple comparison correction described below. The resultant computations of qEEG-frequency with significant relationship with sex and age sex are thus controlled for differences in head size.

### Correction for multiple comparisons

For each participant, we computed a total of 372 variables for spectral analysis (31 EEG locations X 12 Freq.-bands), and 432 variables (36 region pairs X 12 Freq.-bands) for coherence/phase analysis, during eyes open and eyes closed conditions. For group comparisons and statistical analysis, this large number of variables from each subject requires statistical accounting for multiple comparisons. We implemented a nonparametric statistical testing approach based on repeated random partition of groups described in Maris and Oostenveld (2007) and outlined in the following steps:

1) Perform statistical comparisons of the mean of a particular location-frequency variable from boys and girls (e.g., 3 factor ANOVA) and obtain the *F* statistic and *p*-values of the ANOVA factors, referred to as experimental *F* and *p*-values for each factor.
2) Place the variables sequentially in a single array, i.e., all 84 boy variables first followed by all 62 girl variables.
3) Randomize the order of the above array and select the first 84 variables as set 1 and the remaining 62 variables as set 2.
4) Perform statistical comparisons on sets 1 and 2 similar to step 1 and save the *F* and *p*-values.
5) Repeat steps three and four 10,000 times (Monto-Carlo estimate).
6) Compute the percent of *F* values for each factor in the 10,000 random partitions that exceed experimental *F* values of step 1, this is referred to as false discovery rate (FDR) for that factor.
7) If FDRs <0.05, conclude that experimental *p*-values of step 1 are significant, otherwise accept the null hypothesis (i.e., no difference in means of the variables for each factor).
8) Repeat steps 1–7 for all of 496 region-frequency pairs (coherence) and 372 EEG-Freq. pairs (spectral analysis).

### Muscle activity artifact can potentially contaminate EEG gamma power

EEG gamma signal can be prone to artifacts introduced by muscle activity (e.g., from the neck and temporal regions) as the gamma frequency band overlaps with the high frequency electrical activity generated by muscle contraction (Muthukumaraswamy, 2013). We investigated this potential artifact by comparing the absolute and relative power of the gamma band from the channels closest to the neck, consisting of EEG channels PO7, O1, O2, PO8 (Group 1), with more anterior electrodes P3-CP3-P4-CP4 (Group 2) in TD boys and girls. Our analysis consisted of computing the ratio of [averaged Group 1 (*γ*1–*γ*4 power)]/[averaged Group 2 (*γ*1–*γ*4 power)] for every TD child in our database. A ratio >1 indicates excess activity in Group 1 (electrodes close to neck) compared with Group 2 (farther away from muscle activity). We then performed student-*t* comparisons of these ratios for TD boys (*n* = 84) and girls (*n* = 61) separately, with the null hypothesis of ratio = 1.

## Results

All the numerical results of group comparisons are provided in the Supplementary material in table formats. For the main manuscript, however, we have developed a novel method of presenting the statistical group comparisons of the extensive set of variables (432 for coherence, 372 for spectral power) in a compact and easy-to-visualize method.

### Spectral analyses

Supplementary Tables S1, S2 in the Supplementary material show the numerical results of comparing boys’ and girls’ absolute power levels during eyes- closed and eyes-open conditions for a subset of 372 EEG-Freq. pairs (31 EEG locations X 12 Freq.-bands) that had at least one significant *p*-value in the sex and age factors ANOVA, These tables provide the mean and standard deviation of the absolute powers for boys and girls and ANOVA *F* and *p*-values for the sex and age factors. The tables also provide the result of correlation (Pearson) of the spectral power values of boys and girls with their age. Supplementary Tables S3, S4 have a similar structure to Supplementary Tables S1, S2 showing the numerical results of comparing boys’ and girls’ relative spectral power levels during eyes-closed and eyes-open conditions.

**Figure 2:**
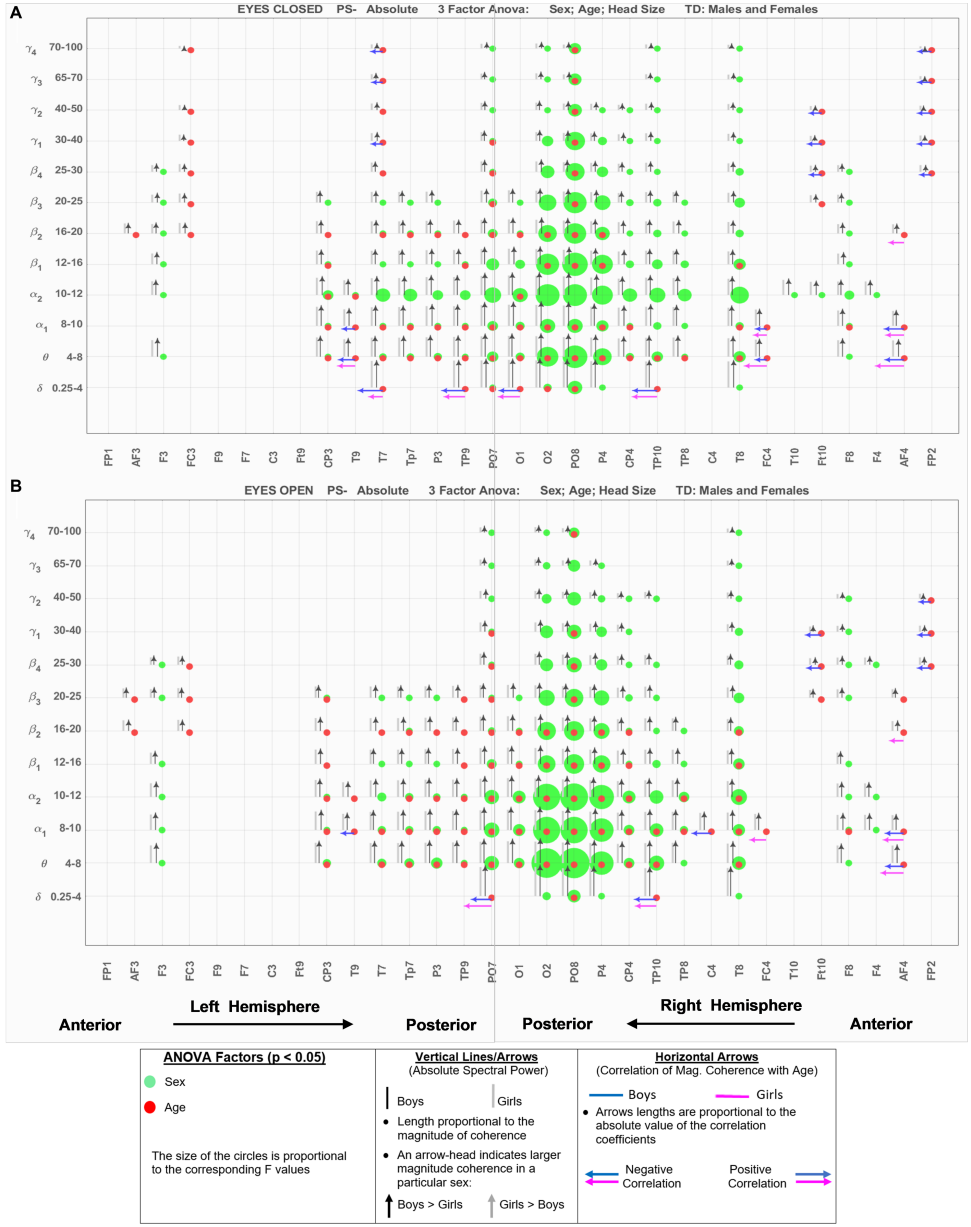
EEG-Frequency pairs with significant (*p* < .05) 3-factor ANOVA (sex, age, head size) differences between boys’ and girls’ Absolute Spectral Power. **(A)** Eyes Closed. **(B)** Eyes Open.

Figure 2 shows the eyes-closed (A) and eyes-open (B) data of Supplementary Tables S1, S2 in a compact graphical presentation that allows us to visually examine the results of the ANOVA sex and age comparisons, the magnitude of absolute spectral powers for each of the 2 sexes, along with their correlation coefficient with age, all in a single graph. In Figure 2, green circles indicate EEG location-frequency with significant differences between boys and girls (from ANOVA sex factor), and red circles indicate significant correlations with age (adjusted *p* < 0.05). The sizes of the circles are proportional to their corresponding *F*.

Black and gray vertical lines/arrows are proportionally related to the magnitude of absolute spectral power of boys and girls, respectively. An arrow-head was placed on the vertical line associated with the specific sex with larger absolute power (e.g., black arrow-head indicates boys had larger absolute spectral power). Horizontal arrows indicate that the EEG location—frequency band had a significant correlation with Age for boys (blue) and girls (magenta); their length is proportional to the absolute value of the correlation coefficient, and the left and right arrows correspond to negative and positive correlations, respectively.

**Figure 3:**
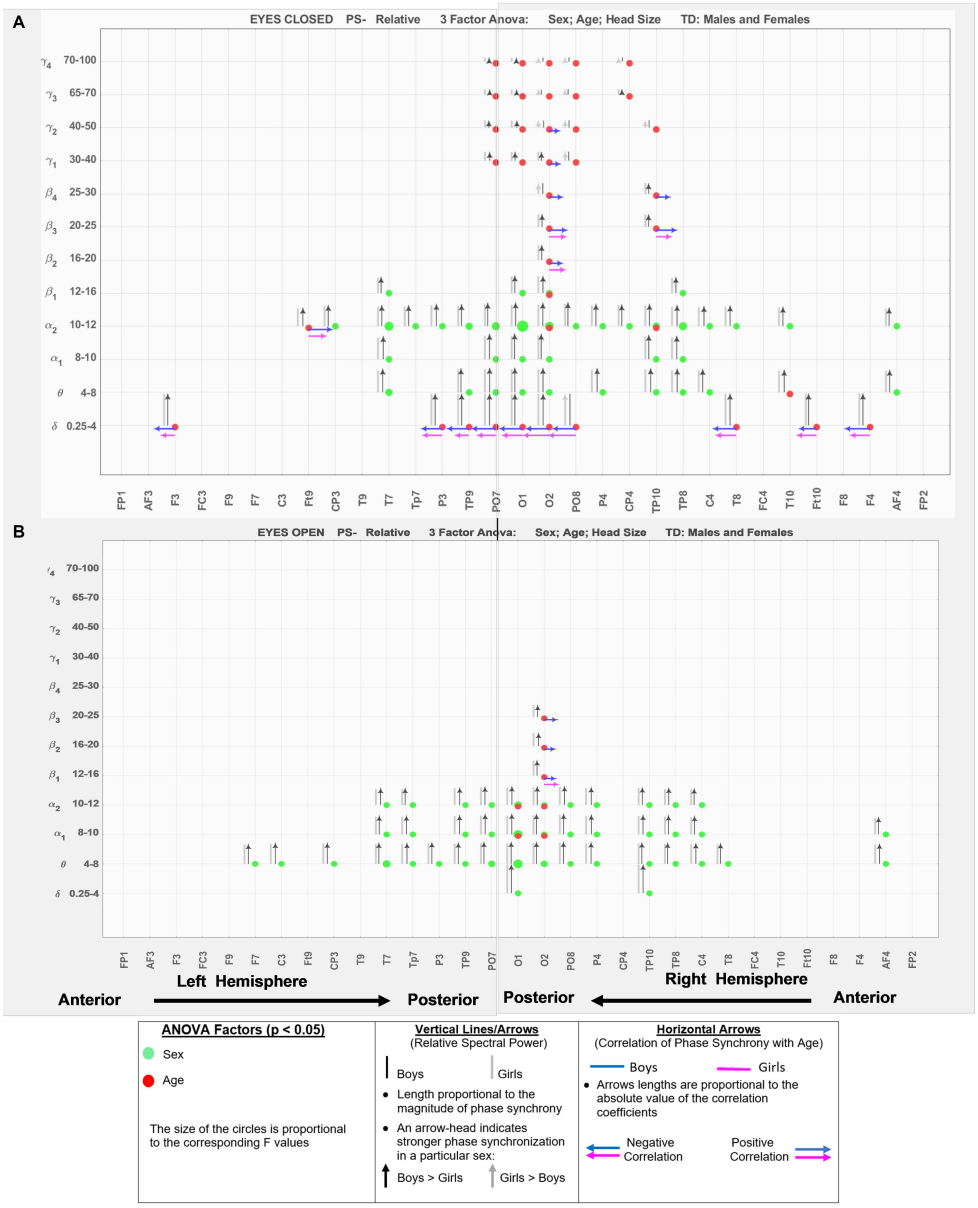
EEG-frequency pairs with significant (*p* < .05) 3-factor ANOVA (sex, age, head size) differences between boys’ and girls’ Relative Spectral Power. **(A)** Eyes Closed. **(B)** Eyes Open.

Figure 3 shows the results for relative spectral powers during eyes closed (A) and eyes-open (B) conditions, in a similar format as Figure 2. Table 4 summarizes the key findings from Figures 2, 3.

**Table 4:** Summary of key observations from Figures 2, 3.

Absolute power	Relative power
Eyes-closed Boys >Girls: wide spread sex differences in both hemispheres and across low and high frequencies The differences were more pronounced in the posterior sites, with the right-hemisphere having larger differences between the sexes. Specifically, O2, PO8, and P4 had the largest inter-sex differences at *δ*, *θ*, *α*1–*α*2, and higher bands *β*1–*γ*1 frequency bands. Frontal sites (F3, and F8) also showed boys with a larger spectral power for the frequency bands spanning from *θ* to *β*4. Age factor: A significant age relationship was observed across the two hemispheres, at both anterior and posterior sites, and at lower frequencies *δ*, *θ*, and *α*1. Negative correlation between spectral power and age was observed prominently at very low (*δ*, *θ*) and very high (*β*4–*γ*4)	Eyes-closed Boys >Girls: both hemispheres (anterior and posterior regions), mainly in *θ*, *α*1, *α*2 bands Age factor was strongly present at the anterior and posterior sites of both hemispheres at both very low (*δ*) and high frequency bands (*α*2 and above), particularly in the occipital sites. There was a negative correlation with age at *δ*, and a positive correlation with age at *α*2 and higher bands for both sexes. This suggests that relative spectral powers are increased at higher frequencies with age and become lower at the very low *δ*
Eyes-open Virtually identical to eyes-closed condition in terms of Boys >Girls, and similar observations for the location and frequency of the sex and age factors	Eyes-open Similar pattern to eyes-closed for the sex factor, in terms of boys >girls at *θ*, *α*1, *α*2 bands Relative spectral powers of both boys and age show age dependence concentrated in the occipital sites. Similar to eyes-closed, positive age correlation for *β*1–*β*3 bands

### Assessing potential muscle activity artifact contaminating EEG gamma power in our study

Our results indicated that for both boys and girls, the ratios for absolute gamma power in Group 1 (PO7, O1, O2, PO8; closest to the neck region)/Group 2 (P3-CP3-P4-CP4-farther away from the neck) were significantly >1 during both eyes open and eyes closed conditions, suggesting the potential existence of muscle artifacts in the gamma band records in the electrodes near the neck region. For the relative spectral power, the ratio was also >1 during eyes-open condition. However, during eyes closed condition, the ratio from relative power was not significantly different than 1, suggesting that relative gamma activity during eyes closed condition is not noticeably affected by the muscle movement in our database.

### Hemispheric asymmetry

**Figure 4:**
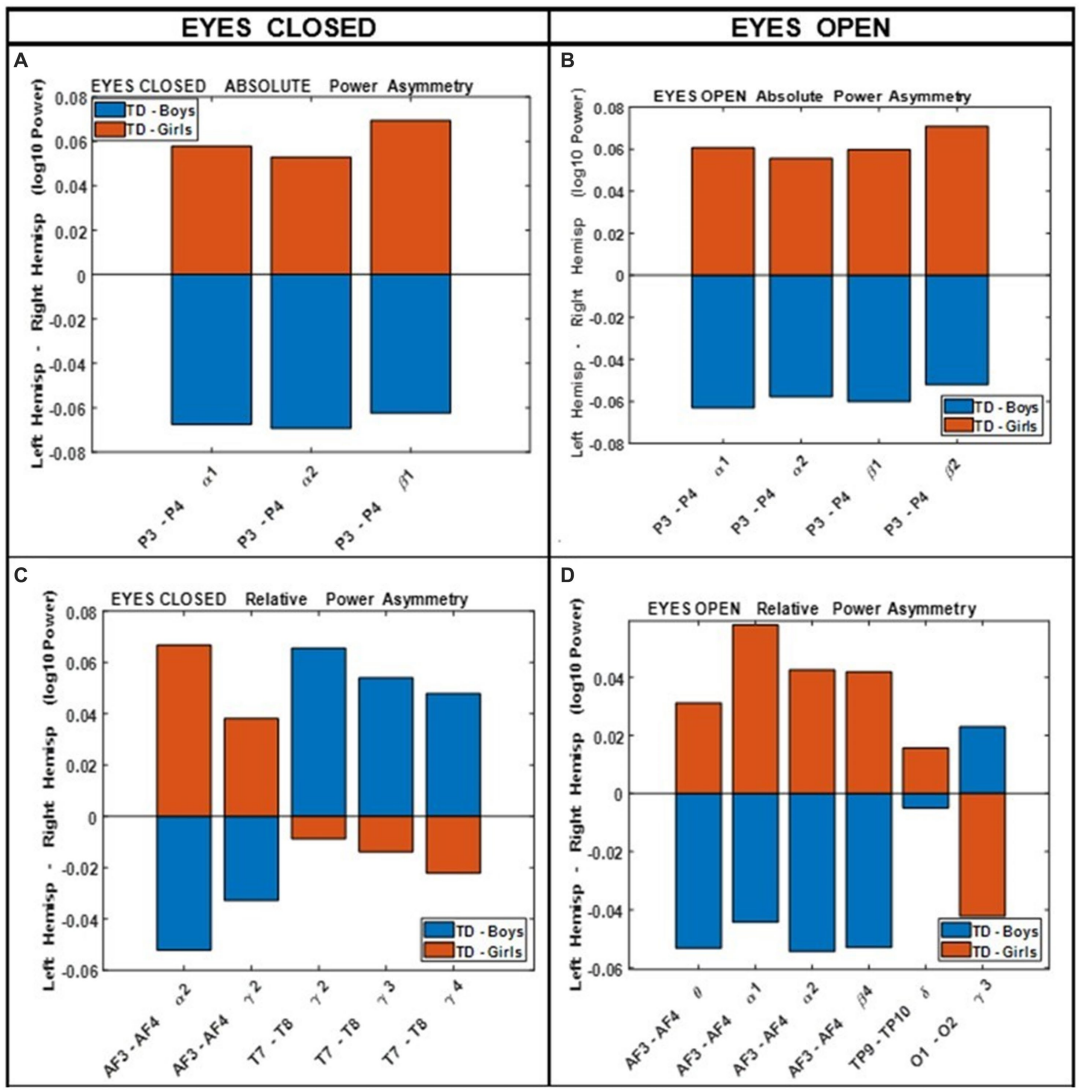
Comparing boys’ and girls’ asymmetry of absolute power **(A,B)** and relative power **(C,D)** during eyes closed (left column) and eyes open (right column) conditions.

Figure 4 shows the results of comparing the asymmetry of spectral powers between the two hemispheres, defined as log10 (spectral power of left hemisphere) − log10 (spectral power of right hemisphere) in TD boys and girls during eyes-closed and eyes-open conditions (right and left columns, respectively). The absolute and relative power asymmetries are shown in the top and bottom rows, respectively. The data of Figure 4 depict EEG-Freq. bands whose hemispheric differences were significant between boys and girls (*p* < 0.05). Table 5 summarizes the observations from Figure 4. Supplementary Table S5 in the Supplementary material shows the numerical values/statistics used to generate Figure 4.

**Table 5:** Summary of key observations from Figure 4.

Absolute power	Relative power
Eyes-open and eyes-closed conditions. Show similar differences between the sexes The major difference between boys and girls is the asymmetry at the right–left parietal power (P3–P4) at *α* and low *β* frequencies: Girls P3 >P4 (left parietal dominance). Boys P3 < P4 (right parietal dominance)	Eyes closed: Girls: Left hemisphere dominance in the frontal site, AF3 >AF4 at *α*, *γ* Right dominance at temporal site *γ* (T7 <T8) Boys: Right hemisphere dominance at frontal; AF3 <AF4 (*α* and *γ*) Left dominance at temporal site *γ* (T7 >T8) Eyes open: Girls: Frontal left dominance (AF3 >AF4) at *θ α*1 *α*2 *β*2; occipital right dominance (O2 >O1) Boys: Frontal right dominance (AF3 <AF4) at *θ α*1 *α*2 *β*2; occipital left dominance (O2 <O1)

### Magnitude coherence and phase synchrony

Supplementary Tables S6, S7 in the Supplementary material show the numerical results of comparing boys’ and girls’ magnitude coherence during eyes-closed and eyes-open conditions for a subset of 432 EEG-Freq. pairs (36 EEG locations X 12 Freq.-bands) that had at least one significant *p*-value in the sex and age factors of ANOVA. These tables provide the mean and standard deviation of the magnitude coherence for boys and girls and ANOVA F and *p*-values for the sex and age factors. The tables also provide the result of correlation (Pearson) of the coherence values of boys and girls with their age. Supplementary Tables S8, S9 have a similar structure to Supplementary Tables S6, S7, showing the numerical results of comparing boys’ and girls’ phase synchrony levels during eyes-closed and eyes-open conditions.

**Figure 5:**
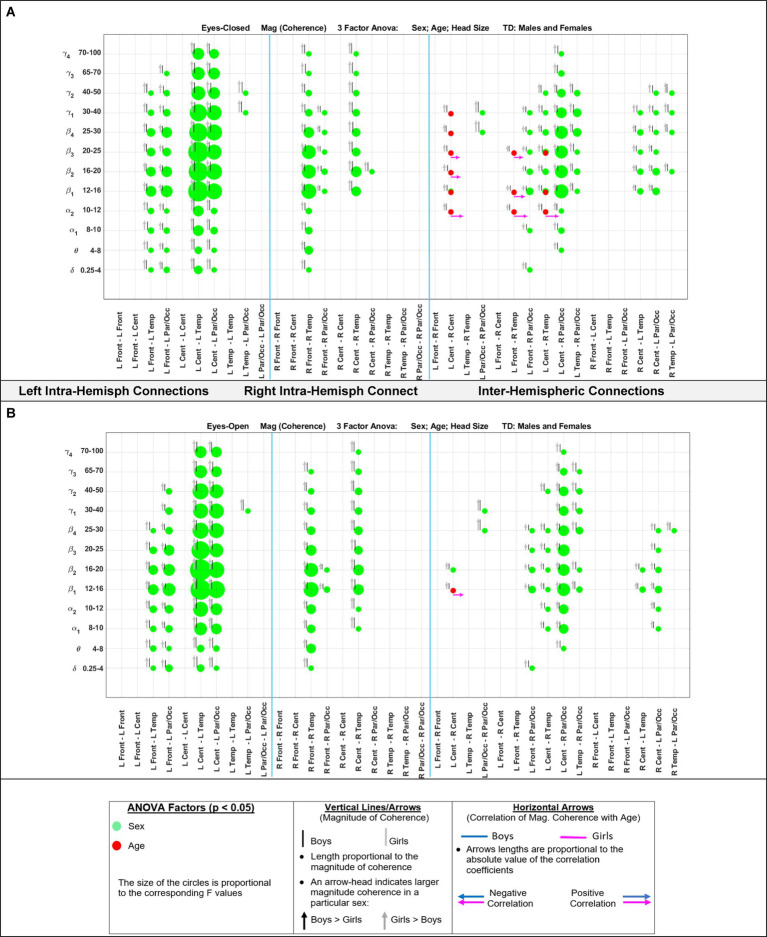
Results of 3-factor ANOVA (sex, age, head size) comparison of boys’ and girls’ magnitude coherence. **(A)** Eyes Closed condition. **(B)** Eyes Open condition.

**Figure 6:**
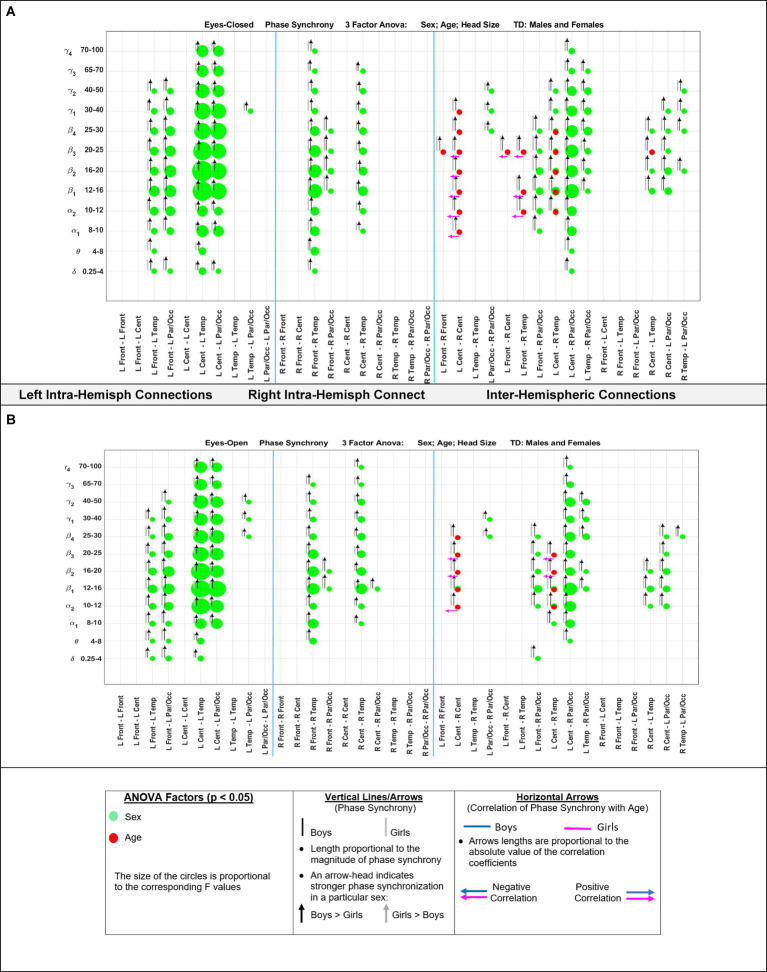
Results of 3-factor ANOVA (sex, age, head size) comparison of boys’ and girls’ phase synchrony. **(A)** Eyes Closed condition. **(B)** Eyes Open condition.

Figure 5 shows the eyes-closed (A) and eyes-open (B) magnitude coherence comparisons of Supplementary Tables S6, S7 in a compact graphical presentation, similar to those of Figures 2, 3, which allows for visual examinations of the ANOVA sex and age comparisons for all of the 432 regions-frequency pairs. Figure 6 provides similar presentation for phase synchrony comparisons for eyes closed (A) and eyes open (B) conditions.

**Figure 7:**
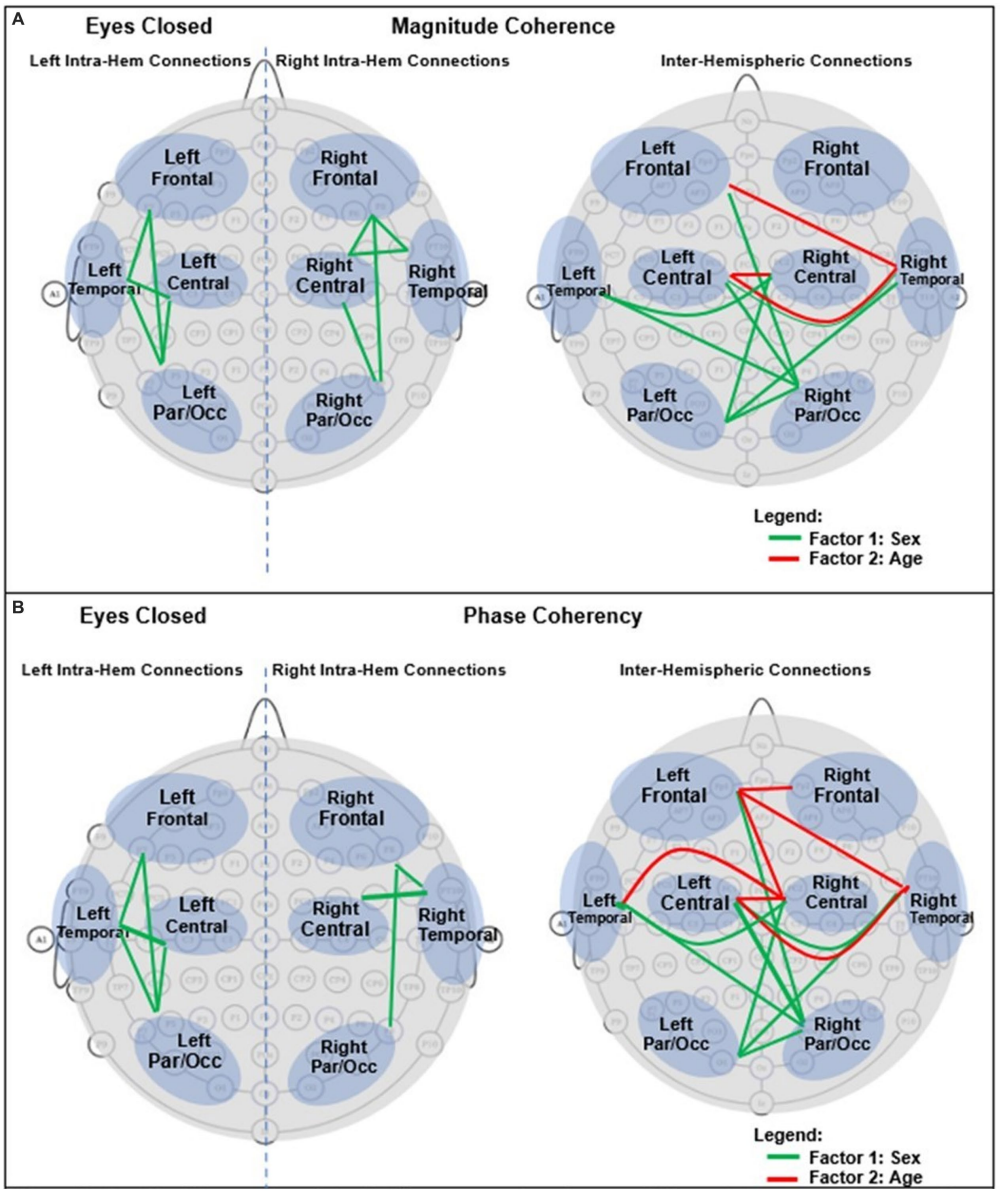
Graphical presentation of the significant connections that were different in boys compared with girls in Eyes Closed. **(A)** Magnitude Coherence. **(B)** Phase Synchrony.

**Figure 8:**
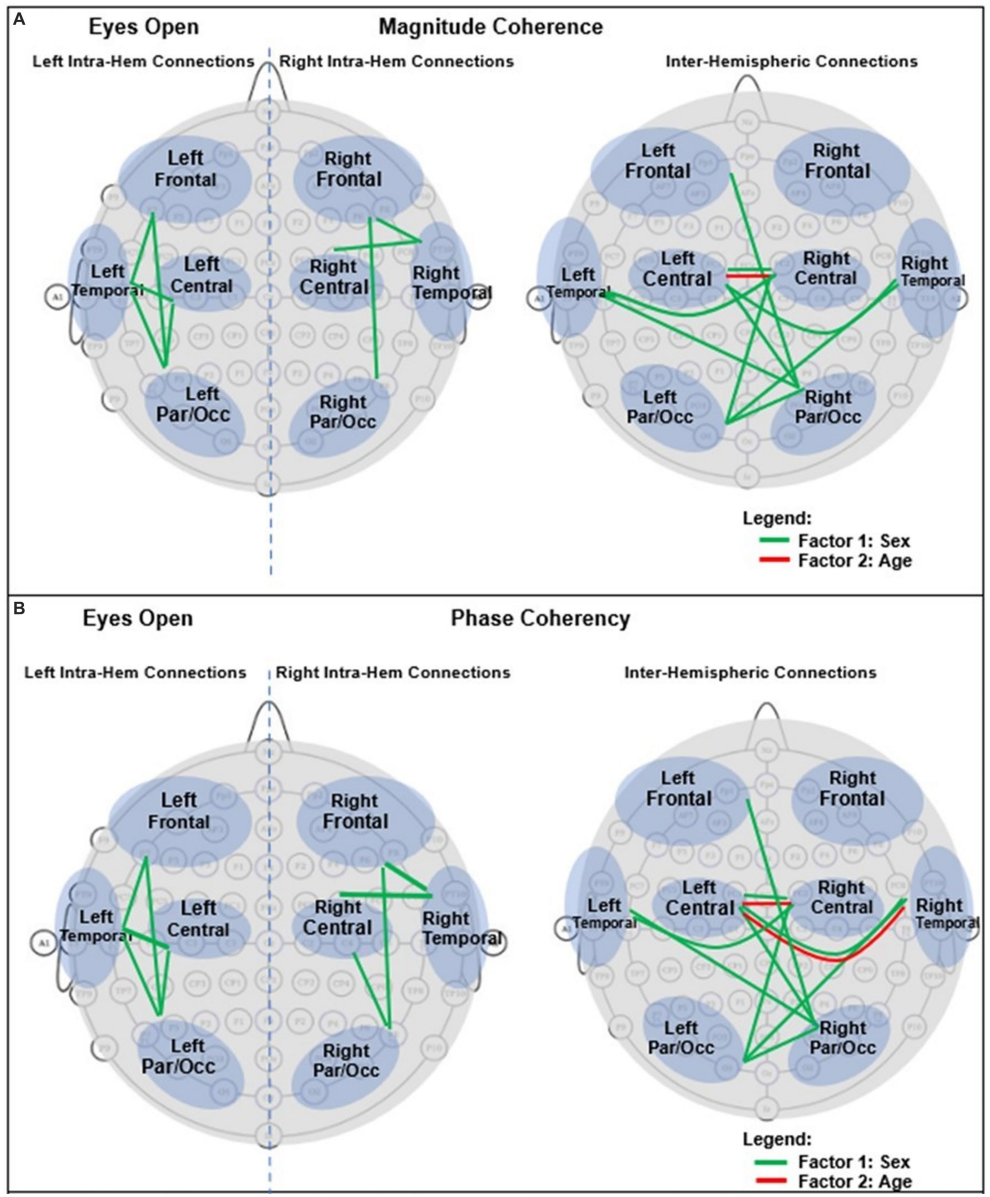
Graphical presentation of the significant connections that were different in boys compared with girls in Eyes Open. **(A)** Magnitude Coherence. **(B)** Phase Synchrony.

For an easier visualization of connections with different magnitude coherence and phase-synchrony in boys and girls, Figure 7 shows head maps corresponding to left and right intra-hemispheric, and inter-hemispherical connections during eyes closed conditions. The lines in Figure 7 magnitude coherence (A) and phase synchrony (B) depict the specific connections between regions where the ANOVA factors were significant (*p* < 0.05) with the same coloring map as those in Figure 5, i.e., a green line between two sites indicates that the sex factor was significant for that connection, and a red line represents a significant age factor. Figure 8 provides head map comparisons, with the same format as of Figure 7, for the eyes open condition.

Table 6 summarizes the key magnitude coherence and phase synchrony differences between TD boys and girls from Figures 5–8.

**Table 6:** Summary of key observations from Figures 5–8.

Magnitude coherence	Phase synchrony
Eyes closed Sex factor: Girls >Boys: Right and left intra-hemispheric, between anterior and posterior sites, entire spectrum (*δ*–*γ*4) Inter-hemispheric connections between parietal/occipital, and central-temporal sites. Age factor: Significant for Inter-hemispheric connections: Between right central -temporal and left central-frontal sites (*α*2 and higher bands) with positive correlation with age for girls	Eyes closed Sex factor: Boys >Girls: Right and left intra-hemispheric, between anterior and posterior sites, entire spectrum (*δ*–*γ*4) Inter-hemispheric connections between parietal/occipital, and central-temporal sites. Age factor: Significant for Inter-hemispheric connections between central -temporal-frontal sites (*α*2 and higher bands) with negative correlation with age for girls
Eyes open Similar results compared to the eyes-closed condition in term of the sex factor (Girls >Boys). Age factor was significant for inter-hemispheric Central connection at *β*1	Eyes open Similar results compared to the eyes-closed condition in term of the sex factor (Girls >Boys). Age factor was significant for inter-hemispheric Central-temporal sites connection at (*α*2 and higher bands)

## Discussion

In this paper, we have provided a comparison of comprehensive qEEG metrics in typically developing (TD) boys and girls during resting state eyes-open and eyes-closed conditions. To the best of our knowledge, this is the first paper that provides such comparisons between TD boys and girls using high density EEG, narrow-band frequencies, and comprehensive qEEG metrics. Our results show there are wide-spread EEG locations and frequencies where TD boys and girls exhibit differences in their absolute and relative spectral powers, hemispheric power asymmetry, magnitude coherence and phase synchrony. Furthermore, we have shown the specific EEG-Freq. pairs whose qEEG metrics are significantly different in the eyes closed vs. eyes-open conditions. By explicitly incorporating sex, age, and head size in statistical analysis, we have shown the specific EEG-Freq. pairs that are sensitive to the Sex and Age factors while controlling for differences in head size that vary with age.

There have only been a handful of studies in the past 30 years that compare qEEG metrics of TD boys and girls in children. Compared to our current study, previous studies had a smaller sample size and limited (1) subset of qEEG metrics, (2) number of EEG channels, (3) number of frequency bands (wide-band frequency ranges), and (4) either eyes open or eyes closed resting state conditions. The limitations of these previous studies greatly reduce the ability to directly compare our findings in terms of observed qEEG differences between TD boys and girls. Nevertheless, we will compare our findings with two previous studies which had 10 or more subjects in each group, and at least 4 nominal frequency bands (*δ*, *θ*, *α*, and *β*).

Clarke et al. (2001) compared the absolute and relative EEG spectral power in typically developing children, 40 boys and 40 girls ages 8–12 years, during eyes closed resting condition. They used four wide-band frequency ranges: *δ* (0.5–2.5 Hz), *θ* (2.5–7.5 Hz), *α* (7.5–13.5 Hz) and *β* (13.5–20.5 Hz). They reported posterior (temporal, parietal, and occipital sites combined) right-hemisphere dominance of absolute power in boys (i.e., posterior right absolute power >posterior left absolute power), and the reverse pattern in girls. This is consistent with our asymmetry results of Figure 4 (top left), which shows that the difference of hemispheric powers, left hemisphere-right hemisphere, is negative for boys (i.e., boys right >boys left), and positive for girls (girls right <girls left). These differences are for P3-P4 (within the posterior region). Clarke et al. (2001) also reported a frontal left hemisphere dominance in boys, and frontal right dominance in girls. The frontal asymmetry difference between boys and girls in our current study did not reach statistical significance, and therefore is not shown in Figure 4. However, as shown in the Supplementary Figure S1, the median difference between the left and the right frontal sites (Fp1, Fp2) in our study is consistent with the reported results of Clarke et al. (2001). As shown in Supplementary Figure S1, boys had a positive Fp1–Fp2 power difference at all frequency bands, indicating a left hemisphere dominance (Fp1 >Fp2 power). Conversely, girls show a negative difference of Fp1–Fp2 power, suggesting a right hemisphere dominance (Fp1 <Fp2 power).

Marosi et al. (1993) reported a sex difference in EEG coherence of typically developing children (*n* = 18 girls and 24 boys,; ages 7.6 to 13.3) during eyes-closed resting condition at their 4 frequency bands defined as *δ* (1.5–3.5 Hz), *θ* (3.75–7.5 Hz), *α* (7.5–12.5 Hz), and *β* (12.5–19 Hz) frequencies. They reported that girls had higher coherences at the four *δ*, *θ*, *α*, and *β* bands; this is consistent with our Figures 5A, 8A results that shows girls with a higher magnitude coherence at several intra- and interhemispheric connections at the *θ*, *α*1, *α*2, *β*1, and *β*2 bands during eyes-closed resting conditions.

### Comparison of our findings with recent studies in young healthy adults

Ko et al. (2021) described a normative qEEG database (4.5–81 years old), and reported that in the young healthy group (15 < age < 20), consisting of *n* = 85 normal males and = 85 normal females, spectral power in the *θ* band was larger (*p* < 0.1) in females compared with male subjects mainly at the central and temporal sites. Cave and Barry (2021) have reported on the differences in the EEG activity (spectral power) during eye-closed resting state in healthy young males (*n* = 40) and females(*n* = 40), with a mean age of 20.4 (range 18–26) years. They reported that females had greater EEG activity compared with males across the scalp in the *δ*, *α* and *β* bands. The reported finding in these two references is opposite to our results, shown in Figures 2, 3, in that the girls in our study had a smaller spectral power than boys. To potentially resolve this seemingly opposite results, we re-examined our data and compared the spectral powers of boys and girls who were closer in age to the participants of Ko et al. (2021) and Cave and Barry (2021). Thus, we separated the groups into 3 age bins: [bin 1] 7–10 years, *N* (boys, girls) = (37, 27); [bin 2] 10–13 years, *N* (boys, girls) = (17, 11); and [bin 3] 13–16 years *N* (boys, girls) = (14, 13). We hypothesized that the results of bin 3 (13–16 years) would be closer to those of the previous work.

Supplementary Figure S2 in the Supplementary material has six panels showing the differences of median absolute spectral power (log10) in the 3 age bins for the EEG channels and *δ*, *θ*, *α*1, *α*2, *β*1, and *β*2 bands. The figures are in a heat map style with a colormap that shows the hotter colors (brown and yellow) correspond to a positive value for the difference of girls − boys (i.e., girls >boys), and the light green and blue (cooler colors) correspond to a negative value for the difference girls-boys (i.e., boys >girls). The top and middle panels of the left column of Supplementary Figure S2, corresponding to *δ*, *θ*, clearly shows cool colors for the 7–10- and 10–13 years bins (i.e., boys >girls) across the scalp that reached significance at the left-hemisphere temporal, parietal and occipital sites (particularly for *θ*).

Interestingly, there appears to be a transition at later ages where 13–16 years girls’ spectral power was larger than boys, although this increase in girls’ spectral power did not reach statistical significance. The bottom left panel of Supplementary Figure S2 (*α*1 band) shows a similar reversal of spectral differences such that girls had higher power after 13 years of age at the majority of the EEG locations. The *α*1 band panel also reveals that the reversal of the relative power apparently initiates in the middle bin (10–13 years) which shows warmer colors compared to the 7–10 years group. The 3 panels in the right column of Supplementary Figure S2, corresponding to *α*2, *β*1, and *β*2 bands, also show a reversal of polarity of girls-boys at 13 years of age, with girls having increased spectral power in the 13–16 years group albeit at fewer and more localized EEG positions (compared to lower frequency bands). The overall results of Supplementary Figure S2 hint at a possible reversal (increase) of the strengths of spectral power of girls compared to boys around 13 years of age.

The results of this post-hoc analysis should be interpreted with caution given the small sample sizes in each of the age bins, particularly the age 13–16 groups. However, sex-dependent changes in brain electrical activity in puberty is supported in past studies, such as Benninger et al. (1984) who examined the spectral power of a 20 s EEG recording (C3, C4, O1, and O2) of 96 healthy children (47 boys and 49 girls), 4–17 years old, who were followed for up to 7 years with serial EEGs. They reported that girls under 6 years of age showed significantly less alpha wave activity compared with boys. However, they reported a higher developmental velocity (change per year) in girls than in boys resulting in girls becoming more similar to boys in brain activity during puberty. An earlier study by Petersén and Olofsson (1971) reported differences between boys and girls in their low frequency activity, which was significantly higher in boys up to 8 years of age, but significantly higher in girls at ages 14 and 15 years.

With regards to coherence analysis, the results of both magnitude coherence and phase synchrony are presented to demonstrate the robustness of results to method of coherence analysis, and to demonstrate that volume conduction artifacts are not the causal factor in the resulting observed coherence. Figures 5–8 demonstrate the similarity in patterns of coherence differences between the two methods. In fact, the phase synchrony analysis, which eliminates volume conduction artifacts, reveal a greater number of significant differences than the magnitude coherence analysis alone. Phase synchrony may therefore be a more sensitive measure for differences associated with sex and age.

In conclusion, the results presented here strongly support the necessity of including sex, age, and head size as covariates in the analysis of qEEG of children, and argue against combining data from boys and girls. Our results also support the utility of narrow-band frequencies, e.g., dividing *α*, *β*, and *γ* band into finer sub-scales. For example, both absolute and relative spectral powers at *α*1 and *α*2 show pronounced differences in their sensitivity to the age and sex factors in both eyes open and eyes closed condition (Figures 2, 3). Therefore, combining *α*1 and *α*2 into a single 8–12 Hz *α* band could result in less sensitivity to the age and sex differences. Another example is in the asymmetry plots of Figure 7 which show significant boy-girl differences in the power asymmetry at *α*2 (but not *α*1), as well as *β*1 and *β*2 (but not *β*3 and *β*4). The results of this study can serve as a comprehensive normative qEEG database for resting state studies in children containing both eyes open and eyes closed paradigms.

Limitations of the current analysis include the limited number of subjects (although greater than most previous studies), and the narrow age range, 5–16 years; younger and older subjects may have different patterns than those described here. Finally, these results are limited to typically developing subjects with no psychiatric or neurodevelopmental disorders, and the generalizability to individuals with these disorders remains to be investigated. Future studies should pursue comparison of sex differences in individuals with specific neurodevelopmental or psychiatric diagnoses.

## Data availability statement

Publicly available datasets were analyzed in this study. This data can be found at: http://fcon_1000.projects.nitrc.org/indi/cmi_healthy_brain_network/sharing_neuro.html.

## Ethics statement

The studies involving humans were approved by the Chesapeake Institutional Review Board (https://www.chesapeakeirb.com/). Prior to conducting the research, written informed consent is obtained from participants ages 18 or older. For participants younger than 18, written consent is obtained from their legal guardians and written assent obtained from the participant. The studies were conducted in accordance with the local legislation and institutional requirements.

## Author contributions

MM and DK: conceptualization. MM, DC, and JF: study design and execution. MM, DK, and DC: data curation. MM: data processing and algorithm development, results visualization, and writing—original draft. MM, DC, DK, and JF: data analysis and interpretation and writing- review and editing. All authors contributed to the article and approved the submitted version.
