# Supplementary material for: Comparison of comprehensive quantitative EEG metrics between typically developing boys and girls in resting state eyes-open and eyes-closed conditions
Source: Front Hum Neurosci. 2023 Nov 6;17:1237651. doi: 10.3389/fnhum.2023.1237651 (PMC10659091; doi:10.3389/fnhum.2023.1237651)

# *Supplementary Material*

Title: Comparison of Comprehensive Quantitative EEG (qEEG) Metrics Between Typically Developing Boys and Girls in Resting State Eyes-Open and Eyes-Closed Conditions.

**Authors**: Mo Modarres, David Cochran , David N. Kennedy , and Jean A. Frazier

**Table S1.** Comparison of **<u>Absolute Spectral Power</u>** (log10) between Boys and Girls during **<u>Eyes-Closed</u>** Condition.

Significant Sex Factor | Significant Age Factor

| Eyes Closed | Absolute Spectral Power (log10) | | | ANOVA | | | | Pearson Correlation with Age ( %) | | | |
|---|---|---|---|---|---|---|---|---|---|---|---|
| | | Boys | Girls | Factor: SEX | | Factor: AGE | | Boys | | Girls | |
| EEG | Freq. | Mean ± Std | Mean ± Std | F | p | F | p | Corr. Coeff | p | Corr. Coeff | p |
| AF4 | θ | 2.3 ± 0.43 | 2.1 ± 0.39 | 1.41 | 0.24 | 2.82 | 0.0027 | -36.6 | 0.00063 | -53.8 | 6.6e-06 |
| | α1 | 2.1 ± 0.44 | 1.9 ± 0.4 | 0.972 | 0.33 | 2.34 | 0.012 | -37.7 | 0.0004 | -35.1 | 0.0052 |
| | β2 | 1.3 ± 0.36 | 1.2 ± 0.34 | 0.622 | 0.43 | 2.01 | 0.033 | -17.6 | 0.11 | -29.6 | 0.02 |
| FP2 | β4 | 0.91 ± 0.42 | 0.85 ± 0.46 | 0.0169 | 0.9 | 2.01 | 0.033 | -23.5 | 0.031 | -16.4 | 0.2 |
| | γ1 | 0.74 ± 0.46 | 0.69 ± 0.48 | 0.0612 | 0.81 | 2.39 | 0.01 | -27.8 | 0.01 | -12.8 | 0.32 |
| | γ2 | 0.61 ± 0.49 | 0.56 ± 0.49 | 0.0823 | 0.77 | 2.5 | 0.0074 | -27.4 | 0.012 | -13.1 | 0.31 |
| | γ3 | 0.42 ± 0.5 | 0.39 ± 0.5 | 0.0991 | 0.75 | 2.18 | 0.02 | -27.3 | 0.012 | -10.1 | 0.44 |
| | γ4 | 0.29 ± 0.5 | 0.27 ± 0.5 | 0.12 | 0.73 | 2.05 | 0.029 | -27.1 | 0.013 | -11.7 | 0.37 |
| AF3 | β2 | 1.4 ± 0.48 | 1.3 ± 0.38 | 0.995 | 0.32 | 2.18 | 0.02 | -10.3 | 0.35 | -9.2 | 0.48 |
| F3 | θ | 2.4 ± 0.57 | 2.1 ± 0.46 | 6.55 | 0.012 | 0.869 | 0.57 | -20.6 | 0.06 | -32.1 | 0.011 |
| | α2 | 2 ± 0.56 | 1.8 ± 0.42 | 6 | 0.016 | 0.552 | 0.86 | 0.697 | 0.95 | -0.661 | 0.96 |
| | β1 | 1.6 ± 0.52 | 1.4 ± 0.4 | 4.94 | 0.028 | 0.699 | 0.74 | -4.59 | 0.68 | -15.3 | 0.23 |
| | β2 | 1.4 ± 0.52 | 1.2 ± 0.41 | 4.99 | 0.027 | 0.914 | 0.53 | -1.28 | 0.91 | -13.7 | 0.29 |
| | β3 | 1.3 ± 0.53 | 1 ± 0.42 | 6.7 | 0.011 | 0.885 | 0.56 | 0.446 | 0.97 | -14.4 | 0.26 |
| | β4 | 1.1 ± 0.55 | 0.86 ± 0.42 | 5.48 | 0.021 | 0.908 | 0.53 | -7.66 | 0.49 | -21.2 | 0.098 |
| FC3 | β2 | 1.4 ± 0.38 | 1.4 ± 0.37 | 0.026 | 0.87 | 2.15 | 0.022 | -1.4 | 0.9 | -5.65 | 0.66 |
| | β3 | 1.2 ± 0.38 | 1.2 ± 0.39 | 0.307 | 0.58 | 2.3 | 0.014 | 1.31 | 0.91 | -5.45 | 0.67 |
| | β4 | 1 ± 0.41 | 0.99 ± 0.4 | 0.04 | 0.84 | 2.17 | 0.02 | -9.61 | 0.38 | -15.8 | 0.22 |
| | γ1 | 0.86 ± 0.45 | 0.81 ± 0.42 | 0.0041 | 0.95 | 2.42 | 0.0095 | -10.7 | 0.33 | -11.7 | 0.36 |
| | γ2 | 0.7 ± 0.48 | 0.65 ± 0.44 | 0.0686 | 0.79 | 2.28 | 0.015 | -7.31 | 0.51 | -11.1 | 0.39 |
| | γ4 | 0.37 ± 0.53 | 0.33 ± 0.47 | 0.126 | 0.72 | 2.1 | 0.025 | -3.71 | 0.74 | -10.7 | 0.41 |
| CP3 | θ | 2.7 ± 0.37 | 2.5 ± 0.41 | 6.92 | 0.0097 | 2.86 | 0.0023 | -18.9 | 0.085 | -34.2 | 0.0066 |
| | α1 | 2.7 ± 0.43 | 2.5 ± 0.47 | 5.6 | 0.02 | 3.23 | 0.00071 | -14.9 | 0.18 | -9.95 | 0.44 |
| | α2 | 2.5 ± 0.47 | 2.2 ± 0.44 | 13 | 0.00046 | 2 | 0.035 | 15.1 | 0.17 | 17.6 | 0.17 |
| | β1 | 1.9 ± 0.36 | 1.8 ± 0.39 | 4.98 | 0.027 | 2.25 | 0.016 | 9.42 | 0.39 | -5.17 | 0.69 |
| | β2 | 1.7 ± 0.33 | 1.6 ± 0.4 | 4.23 | 0.042 | 2.51 | 0.0072 | 11.9 | 0.28 | -5.78 | 0.66 |
| | β3 | 1.6 ± 0.35 | 1.4 ± 0.42 | 4.84 | 0.03 | 1.94 | 0.041 | 13.4 | 0.23 | -6.75 | 0.6 |
| T9 | θ | 2.4 ± 0.48 | 2.3 ± 0.51 | 2.12 | 0.15 | 2.09 | 0.026 | -32.3 | 0.0027 | -33.8 | 0.0072 |
| | α1 | 2.3 ± 0.51 | 2.1 ± 0.55 | 2.11 | 0.15 | 2.84 | 0.0024 | -26 | 0.017 | -17.4 | 0.18 |
| | α2 | 2.1 ± 0.43 | 1.9 ± 0.5 | 5.69 | 0.019 | 1.98 | 0.036 | -0.13 | 0.99 | -2.44 | 0.85 |
| T7 | δ | 3.6 ± 0.42 | 3.5 ± 0.42 | 0.192 | 0.66 | 2.04 | 0.03 | -46.4 | 8.8e-06 | -27.1 | 0.033 |
| | θ | 2.7 ± 0.35 | 2.5 ± 0.37 | 8.71 | 0.0038 | 3.22 | 0.00072 | -36.1 | 0.00075 | -30 | 0.018 |
| | α1 | 2.7 ± 0.42 | 2.4 ± 0.47 | 7.32 | 0.0078 | 2.7 | 0.0038 | -25.8 | 0.018 | -9.73 | 0.45 |
| | α2 | 2.5 ± 0.4 | 2.2 ± 0.44 | 18.3 | 3.8e-05 | 1.25 | 0.26 | 11.2 | 0.31 | 16.6 | 0.2 |
| | β1 | 1.9 ± 0.31 | 1.7 ± 0.35 | 8 | 0.0055 | 1.5 | 0.14 | 1.96 | 0.86 | -0.405 | 0.98 |
| | β2 | 1.7 ± 0.29 | 1.6 ± 0.35 | 5.98 | 0.016 | 2.15 | 0.022 | -4.21 | 0.7 | -4.27 | 0.74 |
| | β3 | 1.5 ± 0.3 | 1.4 ± 0.36 | 5.39 | 0.022 | 1.78 | 0.065 | -1.91 | 0.86 | -2.82 | 0.83 |
| | β4 | 1.4 ± 0.33 | 1.2 ± 0.35 | 2.98 | 0.087 | 2.01 | 0.033 | -18.6 | 0.091 | -16.2 | 0.21 |
| | γ1 | 1.2 ± 0.37 | 1 ± 0.37 | 1.96 | 0.16 | 2.12 | 0.024 | -23.1 | 0.035 | -13.8 | 0.28 |
| | γ2 | 1 ± 0.4 | 0.85 ± 0.38 | 2.41 | 0.12 | 2.11 | 0.024 | -19.5 | 0.075 | -13.7 | 0.29 |
| | γ3 | 0.84 ± 0.46 | 0.66 ± 0.39 | 2.27 | 0.13 | 1.98 | 0.036 | -22 | 0.045 | -16 | 0.22 |
| | γ4 | 0.7 ± 0.46 | 0.53 ± 0.39 | 2.27 | 0.13 | 2.19 | 0.019 | -23.7 | 0.03 | -16.5 | 0.2 |
| Tp7 | θ | 2.7 ± 0.33 | 2.5 ± 0.41 | 9.27 | 0.0029 | 3.11 | 0.001 | -36.7 | 0.00059 | -38.8 | 0.0018 |
| | α1 | 2.7 ± 0.42 | 2.4 ± 0.49 | 6.65 | 0.011 | 2.9 | 0.002 | -23.5 | 0.031 | -8.74 | 0.5 |
| | α2 | 2.5 ± 0.43 | 2.2 ± 0.42 | 17 | 6.8e-05 | 1.8 | 0.062 | 8.27 | 0.45 | 17.1 | 0.18 |
| | β1 | 1.9 ± 0.31 | 1.7 ± 0.35 | 8.25 | 0.0048 | 1.93 | 0.042 | 0.323 | 0.98 | -8.31 | 0.52 |

| | | | | | | | | | | | |
|---|---|---|---|---|---|---|---|---|---|---|---|
| | β2 | 1.7 ± 0.28 | 1.6 ± 0.37 | 5.73 | 0.018 | 2.25 | 0.016 | -4.18 | 0.71 | -11.4 | 0.38 |
| | β3 | 1.6 ± 0.27 | 1.4 ± 0.37 | 6.6 | 0.011 | 1.56 | 0.12 | -1.85 | 0.87 | -11.5 | 0.37 |
| P3 | θ | 2.8 ± 0.36 | 2.5 ± 0.44 | 9.51 | 0.0025 | 2.8 | 0.0028 | -25.5 | 0.019 | -37.7 | 0.0025 |
| | α1 | 2.9 ± 0.45 | 2.6 ± 0.54 | 6.56 | 0.012 | 2.69 | 0.004 | -19.6 | 0.073 | -16.9 | 0.19 |
| | α2 | 2.6 ± 0.48 | 2.3 ± 0.51 | 12.6 | 0.00057 | 1.35 | 0.21 | 13.9 | 0.21 | 6.58 | 0.61 |
| | β1 | 2 ± 0.33 | 1.8 ± 0.41 | 6.35 | 0.013 | 1.48 | 0.15 | 6.72 | 0.54 | -14.3 | 0.27 |
| | β2 | 1.8 ± 0.31 | 1.6 ± 0.41 | 5.32 | 0.023 | 2.16 | 0.021 | 5.87 | 0.6 | -16.1 | 0.21 |
| | β3 | 1.6 ± 0.3 | 1.4 ± 0.42 | 6.07 | 0.015 | 1.58 | 0.11 | 10.2 | 0.36 | -14.9 | 0.25 |
| TP9 | δ | 3.6 ± 0.45 | 3.5 ± 0.47 | 0.222 | 0.64 | 2.41 | 0.0096 | -43.9 | 2.9e-05 | -39.7 | 0.0014 |
| | θ | 2.8 ± 0.39 | 2.6 ± 0.44 | 5.85 | 0.017 | 3.94 | 6.9e-05 | -34.8 | 0.0012 | -34 | 0.0069 |
| | α1 | 2.8 ± 0.49 | 2.6 ± 0.53 | 5.87 | 0.017 | 3.14 | 0.00095 | -20 | 0.067 | -11.8 | 0.36 |
| | α2 | 2.6 ± 0.48 | 2.3 ± 0.51 | 13 | 0.00045 | 1.82 | 0.057 | 14.5 | 0.19 | 10.3 | 0.43 |
| | β1 | 2 ± 0.37 | 1.8 ± 0.4 | 5.89 | 0.017 | 2.03 | 0.031 | -1.03 | 0.93 | -9.06 | 0.48 |
| | β2 | 1.8 ± 0.33 | 1.7 ± 0.41 | 3.56 | 0.062 | 2.85 | 0.0024 | -6.38 | 0.56 | -11.5 | 0.37 |
| PO7 | δ | 3.8 ± 0.36 | 3.5 ± 0.5 | 4.56 | 0.035 | 3.82 | 0.0001 | -43.2 | 4.2e-05 | -54.8 | 4e-06 |
| | θ | 3 ± 0.36 | 2.6 ± 0.52 | 14 | 0.00028 | 4.24 | 2.6e-05 | -37.3 | 0.00047 | -43.2 | 0.00045 |
| | α1 | 3.1 ± 0.49 | 2.7 ± 0.59 | 13.5 | 0.00037 | 3.07 | 0.0012 | -21.9 | 0.045 | -21.7 | 0.09 |
| | α2 | 2.9 ± 0.48 | 2.5 ± 0.56 | 21.1 | 1.1e-05 | 1.48 | 0.15 | 19.6 | 0.074 | -0.297 | 0.98 |
| | β1 | 2.3 ± 0.31 | 2 ± 0.45 | 16.3 | 9.4e-05 | 1.94 | 0.04 | 1.68 | 0.88 | -24.9 | 0.051 |
| | β2 | 2 ± 0.29 | 1.8 ± 0.44 | 11.6 | 0.00089 | 2.88 | 0.0022 | -5.08 | 0.65 | -24.7 | 0.053 |
| | β3 | 1.8 ± 0.27 | 1.5 ± 0.43 | 11.1 | 0.0012 | 2.11 | 0.024 | 2.84 | 0.8 | -27.8 | 0.029 |
| | β4 | 1.6 ± 0.26 | 1.3 ± 0.44 | 7.67 | 0.0065 | 2.1 | 0.025 | -16.1 | 0.14 | -41.6 | 0.00078 |
| | γ1 | 1.4 ± 0.28 | 1.1 ± 0.44 | 6.62 | 0.011 | 2.14 | 0.022 | -20.9 | 0.056 | -41.3 | 0.00085 |
| | γ2 | 1.2 ± 0.33 | 0.95 ± 0.43 | 5.91 | 0.017 | 1.85 | 0.053 | -18 | 0.1 | -39.6 | 0.0015 |
| | γ3 | 0.97 ± 0.38 | 0.74 ± 0.44 | 5.69 | 0.019 | 1.75 | 0.07 | -19.4 | 0.077 | -40.6 | 0.0011 |
| | γ4 | 0.82 ± 0.39 | 0.6 ± 0.45 | 5.08 | 0.026 | 1.83 | 0.057 | -19.2 | 0.08 | -42.5 | 0.00058 |
| O1 | δ | 3.8 ± 0.38 | 3.6 ± 0.59 | 0.783 | 0.38 | 1.97 | 0.038 | -39.7 | 0.00019 | -40.9 | 0.00097 |
| | θ | 3 ± 0.37 | 2.7 ± 0.53 | 10.6 | 0.0015 | 3.7 | 0.00015 | -42.7 | 5e-05 | -40.1 | 0.0012 |
| | α1 | 3.2 ± 0.51 | 2.7 ± 0.6 | 11.4 | 0.00098 | 3.33 | 0.0005 | -27.1 | 0.013 | -26.4 | 0.038 |
| | α2 | 3 ± 0.49 | 2.5 ± 0.58 | 19.5 | 2.3e-05 | 1.95 | 0.039 | 13.6 | 0.22 | -2.87 | 0.82 |
| | β1 | 2.3 ± 0.33 | 2 ± 0.45 | 11.7 | 0.00087 | 1.92 | 0.043 | -6.02 | 0.59 | -22.8 | 0.074 |
| | β2 | 2.1 ± 0.29 | 1.8 ± 0.45 | 8.09 | 0.0052 | 2.63 | 0.0049 | -11.7 | 0.29 | -24.5 | 0.054 |
| | β3 | 1.8 ± 0.27 | 1.6 ± 0.45 | 7.45 | 0.0073 | 1.3 | 0.23 | -3.5 | 0.75 | -21.7 | 0.091 |
| O2 | δ | 3.8 ± 0.35 | 3.5 ± 0.5 | 9.96 | 0.002 | 2.68 | 0.0041 | -37.7 | 0.0004 | -53.8 | 6.5e-06 |
| | θ | 3.1 ± 0.34 | 2.7 ± 0.47 | 27.4 | 7.4e-07 | 4.33 | 1.9e-05 | -36 | 0.0008 | -48.6 | 6.3e-05 |
| | α1 | 3.2 ± 0.51 | 2.7 ± 0.6 | 18.8 | 3.1e-05 | 2.93 | 0.0019 | -19.9 | 0.07 | -23.1 | 0.071 |
| | α2 | 3 ± 0.5 | 2.5 ± 0.55 | 30.3 | 2.1e-07 | 1.86 | 0.051 | 24.4 | 0.025 | -1.82 | 0.89 |
| | β1 | 2.3 ± 0.31 | 2 ± 0.42 | 29.1 | 3.6e-07 | 1.96 | 0.039 | 3.15 | 0.78 | -27.2 | 0.033 |
| | β2 | 2.1 ± 0.27 | 1.8 ± 0.42 | 22.7 | 5.5e-06 | 2.73 | 0.0036 | 0.167 | 0.99 | -27.1 | 0.033 |
| | β3 | 1.9 ± 0.26 | 1.5 ± 0.41 | 22.1 | 7e-06 | 1.55 | 0.12 | 12.8 | 0.25 | -27.6 | 0.03 |
| | β4 | 1.6 ± 0.25 | 1.3 ± 0.4 | 16.5 | 8.6e-05 | 1.32 | 0.22 | 0.399 | 0.97 | -39.7 | 0.0014 |
| | γ1 | 1.4 ± 0.28 | 1.1 ± 0.42 | 14 | 0.00028 | 1.16 | 0.33 | -5.78 | 0.6 | -38.4 | 0.0021 |
| | γ2 | 1.2 ± 0.34 | 0.97 ± 0.43 | 8.27 | 0.0048 | 1.1 | 0.36 | -4.3 | 0.7 | -36.9 | 0.0032 |
| | γ3 | 1 ± 0.4 | 0.77 ± 0.46 | 6.48 | 0.012 | 1.19 | 0.3 | -5.84 | 0.6 | -35.6 | 0.0045 |
| | γ4 | 0.88 ± 0.42 | 0.63 ± 0.46 | 5.84 | 0.017 | 1.25 | 0.26 | -4.85 | 0.66 | -36.5 | 0.0036 |
| PO8 | δ | 3.7 ± 0.35 | 3.4 ± 0.5 | 18.5 | 3.5e-05 | 3.77 | 0.00012 | -47.7 | 4.5e-06 | -57.1 | 1.3e-06 |
| | θ | 3 ± 0.41 | 2.5 ± 0.47 | 30.9 | 1.7e-07 | 5.19 | 1.2e-06 | -43 | 4.5e-05 | -51.8 | 1.6e-05 |
| | α1 | 3.1 ± 0.57 | 2.6 ± 0.59 | 18.6 | 3.3e-05 | 2.69 | 0.004 | -24 | 0.028 | -29.5 | 0.02 |
| | α2 | 2.9 ± 0.53 | 2.4 ± 0.54 | 29.8 | 2.6e-07 | 1.26 | 0.26 | 17.1 | 0.12 | -6.1 | 0.64 |
| | β1 | 2.3 ± 0.36 | 1.9 ± 0.4 | 31.7 | 1.2e-07 | 2.2 | 0.019 | -6.16 | 0.58 | -34.1 | 0.0067 |
| | β2 | 2 ± 0.32 | 1.7 ± 0.4 | 28.1 | 5.3e-07 | 3.14 | 0.00095 | -15.3 | 0.16 | -35.2 | 0.005 |
| | β3 | 1.8 ± 0.28 | 1.4 ± 0.39 | 28.9 | 3.8e-07 | 2.05 | 0.03 | -5.67 | 0.61 | -34.9 | 0.0055 |
| | β4 | 1.5 ± 0.28 | 1.2 ± 0.4 | 23.9 | 3.2e-06 | 2.28 | 0.015 | -21.2 | 0.052 | -47.9 | 8.3e-05 |
| | γ1 | 1.4 ± 0.29 | 1 ± 0.4 | 24.8 | 2.1e-06 | 2.19 | 0.019 | -22.6 | 0.039 | -46.2 | 0.00016 |
| | γ2 | 1.2 ± 0.34 | 0.84 ± 0.43 | 17.3 | 6.1e-05 | 2.01 | 0.033 | -19 | 0.084 | -39.7 | 0.0014 |
| | γ3 | 0.96 ± 0.39 | 0.62 ± 0.46 | 16.4 | 9.1e-05 | 2.21 | 0.018 | -16.4 | 0.13 | -36.1 | 0.0039 |
| | γ4 | 0.83 ± 0.41 | 0.5 ± 0.46 | 15.4 | 0.00015 | 2.34 | 0.012 | -16.2 | 0.14 | -37.3 | 0.0028 |
| P4 | δ | 3.7 ± 0.44 | 3.4 ± 0.47 | 5.81 | 0.017 | 1.62 | 0.1 | -36.2 | 0.00072 | -40.4 | 0.0011 |
| | θ | 2.9 ± 0.34 | 2.5 ± 0.46 | 23.2 | 4.4e-06 | 3.53 | 0.00026 | -37.4 | 0.00045 | -44.2 | 0.00033 |
| | α1 | 2.9 ± 0.48 | 2.5 ± 0.56 | 15.4 | 0.00014 | 2.55 | 0.0063 | -22.4 | 0.04 | -24 | 0.06 |
| | α2 | 2.7 ± 0.46 | 2.3 ± 0.52 | 25.8 | 1.4e-06 | 1.48 | 0.15 | 18.7 | 0.089 | -2.42 | 0.85 |
| | β1 | 2.1 ± 0.29 | 1.8 ± 0.41 | 25.6 | 1.6e-06 | 2.01 | 0.033 | -1.55 | 0.89 | -25.2 | 0.048 |
| | β2 | 1.9 ± 0.28 | 1.6 ± 0.41 | 18 | 4.3e-05 | 2.23 | 0.017 | -5.7 | 0.61 | -27.9 | 0.028 |
| | β3 | 1.7 ± 0.3 | 1.3 ± 0.41 | 19.8 | 2e-05 | 1.39 | 0.19 | 3.51 | 0.75 | -27.5 | 0.03 |
| | β4 | 1.4 ± 0.31 | 1.2 ± 0.41 | 13.5 | 0.00035 | 1.47 | 0.15 | -10.8 | 0.33 | -36.8 | 0.0033 |

| | | | | | | | | | | | |
|---|---|---|---|---|---|---|---|---|---|---|---|
| | γ1 | 1.2 ± 0.33 | 0.98 ± 0.43 | 10.6 | 0.0015 | 1.34 | 0.21 | -16.4 | 0.14 | -33.1 | 0.0086 |
| | γ2 | 1.1 ± 0.38 | 0.82 ± 0.46 | 6.75 | 0.011 | 1.34 | 0.21 | -11.9 | 0.28 | -31.3 | 0.013 |
| CP4 | θ | 2.7 ± 0.36 | 2.4 ± 0.46 | 10.1 | 0.0019 | 2.79 | 0.0029 | -39 | 0.00024 | -41.1 | 0.00092 |
| | α1 | 2.7 ± 0.46 | 2.4 ± 0.53 | 8.05 | 0.0053 | 2.22 | 0.017 | -27.6 | 0.011 | -19.5 | 0.13 |
| | α2 | 2.4 ± 0.42 | 2.1 ± 0.47 | 17.7 | 5e-05 | 1.19 | 0.3 | 12.5 | 0.26 | 6.28 | 0.63 |
| | β1 | 1.9 ± 0.29 | 1.6 ± 0.43 | 10.4 | 0.0016 | 1.61 | 0.1 | -6.47 | 0.56 | -18.3 | 0.16 |
| | β2 | 1.7 ± 0.29 | 1.5 ± 0.44 | 6.69 | 0.011 | 1.79 | 0.062 | -10.1 | 0.36 | -22.2 | 0.083 |
| | β3 | 1.5 ± 0.3 | 1.2 ± 0.43 | 9.14 | 0.0031 | 1.16 | 0.32 | -0.817 | 0.94 | -23.5 | 0.066 |
| | β4 | 1.3 ± 0.33 | 1 ± 0.43 | 6.98 | 0.0094 | 1.07 | 0.39 | -13.2 | 0.23 | -34.5 | 0.006 |
| | γ1 | 1.1 ± 0.35 | 0.85 ± 0.46 | 5.47 | 0.021 | 1.06 | 0.4 | -16.3 | 0.14 | -33.1 | 0.0087 |
| | γ2 | 0.9 ± 0.4 | 0.69 ± 0.48 | 4.92 | 0.028 | 0.966 | 0.48 | -10.8 | 0.33 | -32.8 | 0.0092 |
| TP10 | δ | 3.6 ± 0.34 | 3.4 ± 0.45 | 3.45 | 0.066 | 3.21 | 0.00075 | -46 | 1.1e-05 | -46.8 | 0.00013 |
| | θ | 2.8 ± 0.36 | 2.5 ± 0.43 | 13.8 | 0.00031 | 2.53 | 0.0066 | -34.9 | 0.0011 | -40.3 | 0.0012 |
| | α1 | 2.8 ± 0.51 | 2.5 ± 0.53 | 10.4 | 0.0016 | 1.92 | 0.044 | -18.2 | 0.097 | -18.6 | 0.15 |
| | α2 | 2.6 ± 0.45 | 2.3 ± 0.5 | 19.7 | 2.1e-05 | 1.09 | 0.38 | 25.2 | 0.021 | 5.36 | 0.68 |
| | β1 | 2 ± 0.33 | 1.8 ± 0.36 | 12.8 | 0.0005 | 0.974 | 0.47 | 2.05 | 0.85 | -16.4 | 0.2 |
| | β2 | 1.8 ± 0.31 | 1.6 ± 0.36 | 9.71 | 0.0023 | 1.4 | 0.18 | -4.67 | 0.67 | -17.4 | 0.18 |
| | β3 | 1.6 ± 0.29 | 1.4 ± 0.35 | 10.2 | 0.0018 | 0.829 | 0.61 | 2.55 | 0.82 | -17.6 | 0.17 |
| | β4 | 1.4 ± 0.28 | 1.2 ± 0.35 | 6.63 | 0.011 | 1.1 | 0.36 | -15.1 | 0.17 | -30.2 | 0.017 |
| | γ1 | 1.2 ± 0.3 | 0.99 ± 0.37 | 5.06 | 0.026 | 1.17 | 0.32 | -21.1 | 0.055 | -29.7 | 0.019 |
| | γ2 | 1 ± 0.33 | 0.83 ± 0.41 | 4.69 | 0.032 | 1.34 | 0.21 | -17.5 | 0.11 | -30.1 | 0.017 |
| | γ3 | 0.85 ± 0.37 | 0.65 ± 0.42 | 4.44 | 0.037 | 1.53 | 0.13 | -18.2 | 0.098 | -30.4 | 0.016 |
| | γ4 | 0.71 ± 0.38 | 0.52 ± 0.43 | 4.65 | 0.033 | 1.53 | 0.13 | -18.6 | 0.091 | -30.4 | 0.016 |
| TP8 | θ | 2.8 ± 0.39 | 2.5 ± 0.41 | 7.24 | 0.0081 | 2.04 | 0.03 | -42.8 | 4.8e-05 | -29.8 | 0.019 |
| | α1 | 2.8 ± 0.47 | 2.4 ± 0.5 | 7.45 | 0.0073 | 1.91 | 0.044 | -27.7 | 0.011 | -10.5 | 0.42 |
| | α2 | 2.5 ± 0.41 | 2.2 ± 0.45 | 17.2 | 6.4e-05 | 1.34 | 0.21 | 7.64 | 0.49 | 11.2 | 0.39 |
| | β1 | 2 ± 0.3 | 1.7 ± 0.37 | 10 | 0.002 | 1.23 | 0.27 | -13.6 | 0.22 | -6.33 | 0.63 |
| | β2 | 1.8 ± 0.31 | 1.5 ± 0.36 | 5.25 | 0.024 | 1.54 | 0.13 | -17.4 | 0.11 | -10 | 0.44 |
| | β3 | 1.6 ± 0.3 | 1.4 ± 0.36 | 5.41 | 0.022 | 0.974 | 0.47 | -8.41 | 0.45 | -10.1 | 0.44 |
| T8 | δ | 3.6 ± 0.49 | 3.4 ± 0.47 | 4.86 | 0.029 | 1.66 | 0.091 | -32 | 0.003 | -43.1 | 0.00048 |
| | θ | 2.7 ± 0.4 | 2.4 ± 0.41 | 14.6 | 0.00022 | 2.71 | 0.0038 | -37.2 | 0.00049 | -45.2 | 0.00023 |
| | α1 | 2.7 ± 0.5 | 2.4 ± 0.51 | 9.82 | 0.0022 | 2.5 | 0.0072 | -23.2 | 0.033 | -24.2 | 0.058 |
| | α2 | 2.5 ± 0.44 | 2.1 ± 0.46 | 22.7 | 5.5e-06 | 1.57 | 0.12 | 14.9 | 0.18 | -2.84 | 0.83 |
| | β1 | 1.9 ± 0.34 | 1.7 ± 0.37 | 14.9 | 0.00019 | 2.12 | 0.024 | -3.62 | 0.74 | -21.7 | 0.09 |
| | β2 | 1.7 ± 0.33 | 1.5 ± 0.36 | 10.4 | 0.0016 | 1.9 | 0.046 | -10.8 | 0.33 | -25.4 | 0.046 |
| | β3 | 1.5 ± 0.34 | 1.3 ± 0.34 | 13.3 | 0.0004 | 1.33 | 0.21 | -5.25 | 0.64 | -27.2 | 0.032 |
| | β4 | 1.3 ± 0.36 | 1.1 ± 0.36 | 9.84 | 0.0022 | 1.24 | 0.27 | -19.9 | 0.07 | -37.7 | 0.0025 |
| | γ1 | 1.2 ± 0.39 | 0.92 ± 0.38 | 7.93 | 0.0057 | 0.999 | 0.45 | -20.3 | 0.065 | -33.4 | 0.008 |
| | γ2 | 1 ± 0.42 | 0.78 ± 0.41 | 5.83 | 0.017 | 0.922 | 0.52 | -15.7 | 0.15 | -30.7 | 0.015 |
| | γ3 | 0.82 ± 0.45 | 0.6 ± 0.44 | 5.88 | 0.017 | 0.819 | 0.62 | -14.5 | 0.19 | -29.2 | 0.021 |
| | γ4 | 0.69 ± 0.46 | 0.47 ± 0.43 | 5.97 | 0.016 | 0.702 | 0.73 | -13.9 | 0.21 | -28.2 | 0.026 |
| FC4 | θ | 2.4 ± 0.48 | 2.2 ± 0.47 | 0.0658 | 0.8 | 1.98 | 0.036 | -23 | 0.035 | -42.5 | 0.00058 |
| | α1 | 2.2 ± 0.44 | 2.1 ± 0.47 | 0.0427 | 0.84 | 2.35 | 0.012 | -22.8 | 0.037 | -26.1 | 0.041 |
| T10 | α2 | 2.2 ± 0.56 | 2 ± 0.56 | 7.2 | 0.0083 | 1.13 | 0.35 | 7.76 | 0.48 | -1.01 | 0.94 |
| Ft10 | α2 | 1.9 ± 0.44 | 1.7 ± 0.43 | 5.19 | 0.024 | 0.891 | 0.55 | -1.81 | 0.87 | 9.33 | 0.47 |
| | β3 | 1.1 ± 0.39 | 0.95 ± 0.38 | 2.64 | 0.11 | 2.11 | 0.024 | -14.3 | 0.19 | -7.94 | 0.54 |
| | β4 | 0.92 ± 0.4 | 0.79 ± 0.4 | 1.78 | 0.18 | 2.61 | 0.0051 | -24.9 | 0.023 | -16.2 | 0.21 |
| | γ1 | 0.76 ± 0.41 | 0.65 ± 0.41 | 0.901 | 0.34 | 2.66 | 0.0045 | -28 | 0.0098 | -15.3 | 0.23 |
| | γ2 | 0.63 ± 0.43 | 0.52 ± 0.42 | 1.04 | 0.31 | 2.29 | 0.014 | -25 | 0.022 | -15.4 | 0.23 |
| F8 | θ | 2.4 ± 0.46 | 2.1 ± 0.39 | 6.96 | 0.0094 | 1.62 | 0.1 | -32.7 | 0.0024 | -41.4 | 0.00082 |
| | α1 | 2.3 ± 0.47 | 2 ± 0.44 | 4.86 | 0.029 | 2.34 | 0.012 | -25.8 | 0.018 | -20.9 | 0.1 |
| | α2 | 2 ± 0.42 | 1.8 ± 0.38 | 11.6 | 0.00091 | 1.86 | 0.052 | 2.17 | 0.84 | -0.515 | 0.97 |
| | β1 | 1.6 ± 0.41 | 1.4 ± 0.34 | 5.47 | 0.021 | 1.41 | 0.18 | -8.51 | 0.44 | -18.3 | 0.15 |
| | β2 | 1.4 ± 0.39 | 1.2 ± 0.33 | 5.02 | 0.027 | 1.42 | 0.17 | -9.62 | 0.38 | -17.8 | 0.17 |
| | β3 | 1.2 ± 0.41 | 1 ± 0.33 | 6.13 | 0.015 | 1.23 | 0.27 | -5.59 | 0.61 | -17.9 | 0.16 |
| | β4 | 1.1 ± 0.43 | 0.85 ± 0.35 | 5.31 | 0.023 | 1.28 | 0.25 | -13.5 | 0.22 | -24.8 | 0.052 |
| F4 | α2 | 2 ± 0.51 | 1.8 ± 0.37 | 5.58 | 0.02 | 0.529 | 0.88 | -1.43 | 0.9 | -9.16 | 0.48 |

**Table S2.** Comparison of **Absolute Spectral Power** (log10) between Boys and Girls during **Eyes-Open** Condition

Significant Sex Factor (green) — Significant Age Factor (red)

| Eyes Open | Absolute Spectral Power (log10) | | | ANOVA | | | | Pearson Correlation with Age (%) | | | |
|---|---|---|---|---|---|---|---|---|---|---|---|
| | | Boys | Girls | Factor: SEX | | Factor: AGE | | Boys | | Girls | |
| EEG | Freq. | Mean ± Std | Mean ± Std | F | p | F | p | Corr. Coeff | p | Corr. Coeff | p |
| AF4 | θ | 2.3 ± 0.42 | 2.1 ± 0.4 | 1.27 | 0.26 | 2.2 | 0.019 | -34.3 | 0.0014 | -42.7 | 0.00054 |
| | α1 | 2 ± 0.4 | 1.8 ± 0.36 | 2.36 | 0.13 | 2.82 | 0.0026 | -34.8 | 0.0012 | -38.3 | 0.0021 |
| | β2 | 1.3 ± 0.37 | 1.2 ± 0.36 | 0.702 | 0.4 | 2.21 | 0.018 | -20.8 | 0.057 | -26.8 | 0.035 |
| | β3 | 1.2 ± 0.39 | 1 ± 0.37 | 1.11 | 0.29 | 2.03 | 0.031 | -18.3 | 0.095 | -24.1 | 0.06 |
| FP2 | β4 | 0.97 ± 0.44 | 0.88 ± 0.44 | 0.0912 | 0.76 | 2.21 | 0.018 | -26.3 | 0.015 | -12.7 | 0.33 |
| | γ1 | 0.8 ± 0.48 | 0.73 ± 0.45 | 0.0006 | 0.98 | 2.22 | 0.018 | -26.1 | 0.016 | -10.1 | 0.43 |
| | γ2 | 0.65 ± 0.5 | 0.58 ± 0.46 | 0.0111 | 0.92 | 2.17 | 0.02 | -23.8 | 0.029 | -10.5 | 0.42 |
| AF3 | β2 | 1.4 ± 0.49 | 1.3 ± 0.38 | 0.993 | 0.32 | 2.2 | 0.019 | -14.2 | 0.2 | -7.24 | 0.58 |
| | β3 | 1.3 ± 0.52 | 1.1 ± 0.38 | 1.65 | 0.2 | 2.04 | 0.031 | -13.3 | 0.23 | -5.26 | 0.68 |
| F3 | θ | 2.4 ± 0.56 | 2.1 ± 0.46 | 6.99 | 0.0093 | 0.82 | 0.62 | -19.7 | 0.072 | -27.9 | 0.028 |
| | α1 | 2.1 ± 0.56 | 1.9 ± 0.49 | 5.16 | 0.025 | 0.919 | 0.52 | -17.7 | 0.11 | -20.6 | 0.11 |
| | α2 | 1.9 ± 0.56 | 1.7 ± 0.45 | 5.22 | 0.024 | 0.847 | 0.59 | -8.44 | 0.45 | -12.4 | 0.34 |
| | β1 | 1.6 ± 0.53 | 1.4 ± 0.41 | 5.17 | 0.025 | 0.75 | 0.69 | -6.25 | 0.57 | -14.1 | 0.27 |
| | β3 | 1.3 ± 0.56 | 1 ± 0.41 | 6.18 | 0.014 | 1.11 | 0.36 | -2.28 | 0.84 | -14.5 | 0.26 |
| | β4 | 1.1 ± 0.58 | 0.88 ± 0.4 | 5.61 | 0.019 | 1.2 | 0.29 | -7.83 | 0.48 | -19.3 | 0.13 |
| FC3 | β2 | 1.4 ± 0.39 | 1.3 ± 0.37 | 0.0259 | 0.87 | 2.29 | 0.014 | -6.01 | 0.59 | -4.93 | 0.7 |
| | β3 | 1.3 ± 0.41 | 1.2 ± 0.38 | 0.602 | 0.44 | 2.13 | 0.023 | -2.1 | 0.85 | -6.93 | 0.59 |
| | β4 | 1.1 ± 0.44 | 0.99 ± 0.38 | 0.458 | 0.5 | 1.97 | 0.038 | -11.7 | 0.29 | -12.4 | 0.34 |
| CP3 | θ | 2.7 ± 0.38 | 2.4 ± 0.39 | 9.77 | 0.0022 | 2.26 | 0.016 | -16.9 | 0.12 | -27.3 | 0.032 |
| | α1 | 2.5 ± 0.4 | 2.2 ± 0.41 | 8.43 | 0.0044 | 3.85 | 9.2e-05 | -13.3 | 0.23 | -15.4 | 0.23 |
| | α2 | 2.2 ± 0.43 | 2 ± 0.41 | 8.08 | 0.0053 | 3.16 | 0.00088 | 4.81 | 0.66 | 1.99 | 0.88 |
| | β1 | 1.9 ± 0.36 | 1.7 ± 0.4 | 3.73 | 0.056 | 2.32 | 0.013 | 6.78 | 0.54 | -5.7 | 0.66 |
| | β2 | 1.7 ± 0.35 | 1.5 ± 0.4 | 3.79 | 0.054 | 2.83 | 0.0026 | 5.53 | 0.62 | -9 | 0.49 |
| | β3 | 1.6 ± 0.38 | 1.4 ± 0.4 | 5.16 | 0.025 | 2.23 | 0.017 | 8.11 | 0.46 | -8.35 | 0.52 |
| T9 | α1 | 2.1 ± 0.46 | 2 ± 0.52 | 2.76 | 0.099 | 2.39 | 0.01 | -25.2 | 0.021 | -20.9 | 0.1 |
| | α2 | 1.9 ± 0.42 | 1.8 ± 0.5 | 1.99 | 0.16 | 2.22 | 0.018 | -15.8 | 0.15 | -8.24 | 0.52 |
| T7 | θ | 2.7 ± 0.34 | 2.4 ± 0.36 | 11.5 | 0.00095 | 2.87 | 0.0022 | -35.5 | 0.00092 | -24.3 | 0.057 |
| | α1 | 2.4 ± 0.36 | 2.2 ± 0.38 | 9.87 | 0.0021 | 3.04 | 0.0013 | -25.9 | 0.017 | -15 | 0.25 |
| | α2 | 2.2 ± 0.35 | 2 ± 0.37 | 10.8 | 0.0014 | 1.9 | 0.046 | -4.01 | 0.72 | 7.74 | 0.55 |
| | β1 | 1.9 ± 0.31 | 1.7 ± 0.34 | 5.6 | 0.02 | 1.54 | 0.13 | -2.34 | 0.83 | -0.195 | 0.99 |
| | β2 | 1.7 ± 0.3 | 1.5 ± 0.34 | 3.76 | 0.055 | 2.2 | 0.019 | -7.24 | 0.51 | -6.19 | 0.63 |
| | β3 | 1.5 ± 0.34 | 1.4 ± 0.35 | 4.46 | 0.037 | 1.79 | 0.064 | -5.33 | 0.63 | -5.92 | 0.65 |
| Tp7 | θ | 2.7 ± 0.33 | 2.4 ± 0.38 | 12.5 | 0.00059 | 2.67 | 0.0043 | -33.2 | 0.002 | -36.2 | 0.0038 |
| | α1 | 2.5 ± 0.37 | 2.2 ± 0.41 | 10.3 | 0.0017 | 3.63 | 0.00019 | -21.9 | 0.045 | -21 | 0.1 |
| | α2 | 2.2 ± 0.39 | 2 ± 0.38 | 10.5 | 0.0016 | 2.49 | 0.0076 | -1.87 | 0.87 | -1.27 | 0.92 |
| | β1 | 1.9 ± 0.3 | 1.7 ± 0.35 | 5.79 | 0.018 | 1.92 | 0.044 | -1.84 | 0.87 | -9.84 | 0.45 |
| | β2 | 1.7 ± 0.29 | 1.5 ± 0.36 | 4.45 | 0.037 | 2.37 | 0.011 | -6.71 | 0.54 | -14 | 0.28 |
| | β3 | 1.5 ± 0.3 | 1.4 ± 0.36 | 5.72 | 0.018 | 1.9 | 0.046 | -4.38 | 0.69 | -13.8 | 0.28 |
| P3 | θ | 2.8 ± 0.34 | 2.5 ± 0.41 | 13.9 | 0.0003 | 2.3 | 0.014 | -21.4 | 0.05 | -34.8 | 0.0056 |
| | α1 | 2.6 ± 0.4 | 2.3 ± 0.45 | 10.1 | 0.0019 | 3.33 | 0.00051 | -18.2 | 0.097 | -25.8 | 0.043 |
| | α2 | 2.3 ± 0.41 | 2.1 ± 0.46 | 9.37 | 0.0027 | 2.05 | 0.029 | 3.04 | 0.78 | -8.07 | 0.53 |
| | β1 | 2 ± 0.33 | 1.8 ± 0.4 | 4.48 | 0.036 | 1.77 | 0.068 | 2.99 | 0.79 | -15.5 | 0.23 |
| | β2 | 1.8 ± 0.32 | 1.6 ± 0.41 | 3.65 | 0.058 | 2.15 | 0.021 | 1.96 | 0.86 | -17.9 | 0.16 |
| | β3 | 1.6 ± 0.33 | 1.4 ± 0.41 | 5.41 | 0.022 | 1.7 | 0.081 | 5.91 | 0.59 | -19.4 | 0.13 |
| TP9 | θ | 2.8 ± 0.37 | 2.5 ± 0.41 | 7.86 | 0.0059 | 3.26 | 0.00063 | -34.3 | 0.0014 | -30.9 | 0.014 |
| | α1 | 2.5 ± 0.41 | 2.3 ± 0.42 | 7.85 | 0.0059 | 3.65 | 0.00018 | -25.4 | 0.02 | -19.8 | 0.12 |
| | α2 | 2.3 ± 0.4 | 2.1 ± 0.43 | 7.63 | 0.0067 | 2.82 | 0.0026 | -4.15 | 0.71 | -1.96 | 0.88 |
| | β1 | 1.9 ± 0.35 | 1.8 ± 0.39 | 3.61 | 0.06 | 2.43 | 0.0091 | -3.46 | 0.75 | -11 | 0.39 |
| | β2 | 1.8 ± 0.32 | 1.6 ± 0.4 | 2.45 | 0.12 | 2.96 | 0.0017 | -9.45 | 0.39 | -14 | 0.28 |
| | β3 | 1.6 ± 0.35 | 1.4 ± 0.41 | 2.6 | 0.11 | 2.22 | 0.017 | -7.83 | 0.48 | -15 | 0.24 |
| PO7 | δ | 3.8 ± 0.38 | 3.5 ± 0.51 | 4.16 | 0.044 | 2.87 | 0.0022 | -38.1 | 0.00035 | -50.9 | 2.4e-05 |
| | θ | 3 ± 0.33 | 2.6 ± 0.48 | 18.4 | 3.6e-05 | 4.46 | 1.3e-05 | -35.9 | 0.0008 | -44.4 | 0.0003 |
| | α1 | 2.8 ± 0.39 | 2.4 ± 0.49 | 19.2 | 2.5e-05 | 4.93 | 2.8e-06 | -25.9 | 0.017 | -35.6 | 0.0045 |
| | α2 | 2.6 ± 0.36 | 2.2 ± 0.48 | 18.3 | 3.9e-05 | 2.98 | 0.0016 | -3.14 | 0.78 | -18.1 | 0.16 |
| | β1 | 2.2 ± 0.3 | 1.9 ± 0.44 | 11.6 | 0.00092 | 2.61 | 0.0052 | -3.38 | 0.76 | -26.3 | 0.039 |
| | β2 | 2 ± 0.28 | 1.7 ± 0.44 | 8.7 | 0.0038 | 3.11 | 0.001 | -6.59 | 0.55 | -29.6 | 0.019 |
| | β3 | 1.8 ± 0.29 | 1.5 ± 0.44 | 8.06 | 0.0053 | 2.64 | 0.0048 | -7.36 | 0.51 | -33.6 | 0.0075 |

| | | | | | | | | | | | |
|---|---|---|---|---|---|---|---|---|---|---|---|
| | β4 | 1.6 ± 0.29 | 1.3 ± 0.44 | `6.02 | 0.016 | `2.47 | 0.008 | -13 | 0.24 | -42.8 | 0.00052 |
| | γ1 | 1.4 ± 0.33 | 1.1 ± 0.44 | 5.95 | 0.016 | 2.36 | 0.011 | -18.6 | 0.091 | -42.3 | 0.00062 |
| | γ2 | 1.2 ± 0.37 | 0.92 ± 0.43 | 5.91 | 0.017 | 1.79 | 0.063 | -15 | 0.17 | -43.2 | 0.00046 |
| | γ3 | 0.96 ± 0.41 | 0.73 ± 0.43 | 5.39 | 0.022 | 1.44 | 0.16 | -10.6 | 0.34 | -41.6 | 0.00076 |
| | γ4 | 0.84 ± 0.42 | 0.62 ± 0.44 | 4.8 | 0.03 | 1.45 | 0.16 | -12.2 | 0.27 | -40.8 | 0.00099 |
| O1 | θ | 3 ± 0.32 | 2.6 ± 0.49 | 13.1 | 0.00045 | 3.64 | 0.00018 | -45 | 1.8e-05 | -39.4 | 0.0015 |
| | α1 | 2.8 ± 0.41 | 2.4 ± 0.49 | 15.4 | 0.00015 | 4.58 | 8.7e-06 | -33.7 | 0.0017 | -33.9 | 0.007 |
| | α2 | 2.6 ± 0.38 | 2.3 ± 0.48 | 15.5 | 0.00014 | 2.85 | 0.0024 | -8.91 | 0.42 | -17.5 | 0.17 |
| | β1 | 2.2 ± 0.31 | 2 ± 0.45 | 8.06 | 0.0053 | 2.32 | 0.013 | -11.9 | 0.28 | -23.1 | 0.071 |
| | β2 | 2 ± 0.28 | 1.8 ± 0.44 | 6.2 | 0.014 | 2.39 | 0.01 | -14.1 | 0.2 | -26.9 | 0.035 |
| | β3 | 1.8 ± 0.28 | 1.6 ± 0.45 | 5.66 | 0.019 | 1.63 | 0.098 | -11.1 | 0.31 | -28 | 0.027 |
| O2 | δ | 3.8 ± 0.36 | 3.5 ± 0.5 | 10.5 | 0.0016 | 1.94 | 0.041 | -33 | 0.0022 | -48.2 | 7.2e-05 |
| | θ | 3 ± 0.3 | 2.6 ± 0.41 | 38.2 | 9.2e-09 | 4.25 | 2.6e-05 | -38.5 | 0.0003 | -48.2 | 7.2e-05 |
| | α1 | 2.9 ± 0.38 | 2.4 ± 0.44 | 34.3 | 4.3e-08 | 4.51 | 1.1e-05 | -24.1 | 0.027 | -37.5 | 0.0026 |
| | α2 | 2.6 ± 0.37 | 2.2 ± 0.43 | 34.2 | 4.5e-08 | 2.9 | 0.002 | -0.749 | 0.95 | -20 | 0.12 |
| | β1 | 2.3 ± 0.29 | 1.9 ± 0.41 | 22.3 | 6.5e-06 | 2.31 | 0.013 | 1.01 | 0.93 | -28.6 | 0.024 |
| | β2 | 2 ± 0.27 | 1.7 ± 0.4 | 23 | 4.8e-06 | 2.58 | 0.0057 | 1.14 | 0.92 | -32.7 | 0.0095 |
| | β3 | 1.8 ± 0.27 | 1.5 ± 0.4 | 19.8 | 1.9e-05 | 1.82 | 0.058 | 6.12 | 0.58 | -33.9 | 0.007 |
| | β4 | 1.6 ± 0.29 | 1.3 ± 0.4 | 16.4 | 9.1e-05 | 1.57 | 0.12 | -1.44 | 0.9 | -42.3 | 0.00062 |
| | γ1 | 1.4 ± 0.32 | 1.1 ± 0.4 | 15.6 | 0.00013 | 1.2 | 0.29 | -4.5 | 0.68 | -41.8 | 0.00073 |
| | γ2 | 1.2 ± 0.4 | 0.93 ± 0.4 | 11.7 | 0.00085 | 0.945 | 0.5 | -2.68 | 0.81 | -40 | 0.0013 |
| | γ3 | 1 ± 0.44 | 0.74 ± 0.43 | 8.87 | 0.0035 | 1.01 | 0.44 | -2.62 | 0.81 | -40.1 | 0.0012 |
| | γ4 | 0.9 ± 0.46 | 0.64 ± 0.44 | 6.58 | 0.012 | 1.07 | 0.39 | -3.18 | 0.77 | -38.8 | 0.0018 |
| PO8 | δ | 3.7 ± 0.35 | 3.4 ± 0.51 | 16 | 0.00011 | 2.49 | 0.0076 | -43.6 | 3.4e-05 | -48.8 | 5.8e-05 |
| | θ | 2.9 ± 0.35 | 2.5 ± 0.44 | 39.4 | 5.8e-09 | 5.35 | 7.6e-07 | -45.3 | 1.5e-05 | -52.8 | 1e-05 |
| | α1 | 2.8 ± 0.43 | 2.3 ± 0.47 | 34.2 | 4.5e-08 | 4.85 | 3.7e-06 | -30.6 | 0.0047 | -41.1 | 0.0009 |
| | α2 | 2.6 ± 0.4 | 2.1 ± 0.44 | 35.2 | 3e-08 | 2.47 | 0.0081 | -9.31 | 0.4 | -22.5 | 0.078 |
| | β1 | 2.2 ± 0.33 | 1.8 ± 0.41 | 25.1 | 1.9e-06 | 2.74 | 0.0034 | -10 | 0.36 | -35.5 | 0.0047 |
| | β2 | 1.9 ± 0.3 | 1.6 ± 0.4 | 24.9 | 2.1e-06 | 3.42 | 0.00037 | -18.1 | 0.099 | -41.9 | 0.00069 |
| | β3 | 1.7 ± 0.28 | 1.4 ± 0.41 | 23.2 | 4.4e-06 | 2.63 | 0.0048 | -15.9 | 0.15 | -39.7 | 0.0014 |
| | β4 | 1.5 ± 0.29 | 1.2 ± 0.41 | 19.1 | 2.6e-05 | 2.51 | 0.0071 | -22.4 | 0.041 | -51.1 | 2.2e-05 |
| | γ1 | 1.3 ± 0.32 | 1 ± 0.43 | 18.3 | 3.8e-05 | 2.39 | 0.01 | -22.7 | 0.038 | -49 | 5.4e-05 |
| | γ2 | 1.1 ± 0.36 | 0.82 ± 0.45 | 16.6 | 8.5e-05 | 1.78 | 0.066 | -15.7 | 0.15 | -44 | 0.00035 |
| | γ3 | 0.97 ± 0.41 | 0.63 ± 0.47 | 14.9 | 0.00018 | 1.82 | 0.058 | -14 | 0.2 | -39.6 | 0.0014 |
| | γ4 | 0.84 ± 0.43 | 0.52 ± 0.47 | 13 | 0.00047 | 1.99 | 0.035 | -13.7 | 0.21 | -39.2 | 0.0016 |
| P4 | δ | 3.7 ± 0.45 | 3.4 ± 0.48 | 5.93 | 0.016 | 1.12 | 0.35 | -33.4 | 0.0019 | -32.9 | 0.009 |
| | θ | 2.8 ± 0.3 | 2.4 ± 0.43 | 29.9 | 2.5e-07 | 3.18 | 0.00083 | -38.8 | 0.00026 | -39.6 | 0.0014 |
| | α1 | 2.6 ± 0.36 | 2.2 ± 0.45 | 30.4 | 2.1e-07 | 3.86 | 9e-05 | -25.6 | 0.019 | -31.1 | 0.014 |
| | α2 | 2.4 ± 0.35 | 2 ± 0.43 | 30.6 | 1.9e-07 | 2.38 | 0.011 | -3.61 | 0.74 | -16.6 | 0.2 |
| | β1 | 2 ± 0.27 | 1.7 ± 0.4 | 22.1 | 7.1e-06 | 2.24 | 0.017 | -4.91 | 0.66 | -26.5 | 0.038 |
| | β2 | 1.8 ± 0.27 | 1.5 ± 0.4 | 20.1 | 1.7e-05 | 2.44 | 0.0089 | -9.66 | 0.38 | -29.9 | 0.018 |
| | β3 | 1.6 ± 0.3 | 1.3 ± 0.4 | 19.2 | 2.5e-05 | 1.93 | 0.042 | -5 | 0.65 | -31.3 | 0.013 |
| | β4 | 1.4 ± 0.33 | 1.2 ± 0.4 | 13.6 | 0.00034 | 1.35 | 0.21 | -13.7 | 0.21 | -38.4 | 0.0021 |
| | γ1 | 1.3 ± 0.36 | 0.96 ± 0.42 | 12.8 | 0.0005 | 1.24 | 0.27 | -15.2 | 0.17 | -35.9 | 0.0041 |
| | γ2 | 1.1 ± 0.41 | 0.78 ± 0.44 | 9.18 | 0.003 | 1.12 | 0.35 | -10.7 | 0.33 | -33.2 | 0.0084 |
| | γ3 | 0.84 ± 0.45 | 0.62 ± 0.45 | 5.88 | 0.017 | 1.11 | 0.36 | -9.39 | 0.4 | -30.4 | 0.016 |
| CP4 | θ | 2.6 ± 0.34 | 2.3 ± 0.46 | 12.5 | 0.00058 | 2.64 | 0.0047 | -40.9 | 0.00011 | -37.8 | 0.0024 |
| | α1 | 2.4 ± 0.38 | 2.1 ± 0.49 | 13.1 | 0.00044 | 3.37 | 0.00045 | -31.2 | 0.0039 | -27.4 | 0.031 |
| | α2 | 2.2 ± 0.34 | 1.9 ± 0.46 | 16.3 | 9.5e-05 | 2.37 | 0.011 | -10.2 | 0.36 | -10.5 | 0.42 |
| | β1 | 1.8 ± 0.3 | 1.6 ± 0.43 | 7.45 | 0.0073 | 2.15 | 0.022 | -11.3 | 0.31 | -20 | 0.12 |
| | β2 | 1.6 ± 0.3 | 1.4 ± 0.44 | 6.75 | 0.011 | 2.21 | 0.018 | -16.1 | 0.14 | -23.5 | 0.066 |
| | β3 | 1.5 ± 0.31 | 1.2 ± 0.44 | 8.6 | 0.004 | 1.79 | 0.062 | -9.11 | 0.41 | -27.3 | 0.031 |
| | β4 | 1.3 ± 0.35 | 1 ± 0.43 | 7.28 | 0.008 | 1.29 | 0.24 | -16.2 | 0.14 | -37.5 | 0.0026 |
| | γ1 | 1.1 ± 0.39 | 0.84 ± 0.44 | 6.58 | 0.012 | 1.06 | 0.4 | -16.9 | 0.12 | -34.3 | 0.0064 |
| | γ2 | 0.89 ± 0.44 | 0.66 ± 0.45 | 5.48 | 0.021 | 0.786 | 0.65 | -8.7 | 0.43 | -34.1 | 0.0066 |
| TP10 | δ | 3.6 ± 0.35 | 3.4 ± 0.46 | 3.24 | 0.074 | 2.02 | 0.032 | -41.3 | 9.5e-05 | -39.6 | 0.0015 |
| | θ | 2.7 ± 0.32 | 2.4 ± 0.38 | 19.1 | 2.7e-05 | 2.53 | 0.0066 | -39.1 | 0.00024 | -39 | 0.0017 |
| | α1 | 2.5 ± 0.37 | 2.2 ± 0.42 | 16.9 | 7.1e-05 | 2.81 | 0.0028 | -23.8 | 0.029 | -25.1 | 0.049 |
| | α2 | 2.3 ± 0.35 | 2 ± 0.4 | 17.2 | 6.4e-05 | 1.84 | 0.054 | -1.44 | 0.9 | -5.92 | 0.65 |
| | β1 | 1.9 ± 0.31 | 1.7 ± 0.35 | 10.1 | 0.0019 | 1.29 | 0.24 | -3.45 | 0.76 | -17.8 | 0.17 |
| | β2 | 1.7 ± 0.29 | 1.5 ± 0.35 | 8.01 | 0.0055 | 1.39 | 0.19 | -7.53 | 0.5 | -21.5 | 0.093 |
| | β3 | 1.6 ± 0.3 | 1.3 ± 0.35 | 6.53 | 0.012 | 1.11 | 0.36 | -5.56 | 0.62 | -20.2 | 0.12 |
| | β4 | 1.4 ± 0.3 | 1.2 ± 0.36 | 5.05 | 0.026 | 1.39 | 0.19 | -17.6 | 0.11 | -30 | 0.018 |
| | γ2 | 1 ± 0.35 | 0.8 ± 0.39 | `4.61 | 0.034 | 1.35 | 0.21 | -16.5 | 0.13 | -27.2 | 0.033 |
| TP8 | θ | 2.7 ± 0.36 | 2.5 ± 0.4 | 7.52 | 0.007 | 1.8 | 0.061 | -44.4 | 2.3e-05 | -26.4 | 0.038 |

| | | | | | | | | | | | |
|---|---|---|---|---|---|---|---|---|---|---|---|
| | α1 | 2.5 ± 0.37 | 2.2 ± 0.42 | 10 | 0.0019 | 2.41 | 0.0097 | -35.1 | 0.0011 | -15 | 0.24 |
| | α2 | 2.3 ± 0.33 | 2 ± 0.4 | 13.2 | 0.00042 | 2.07 | 0.028 | -17.6 | 0.11 | -0.431 | 0.97 |
| | β1 | 1.9 ± 0.29 | 1.7 ± 0.36 | 7.51 | 0.0071 | 1.56 | 0.12 | -20.4 | 0.063 | -8 | 0.54 |
| | β2 | 1.7 ± 0.29 | 1.5 ± 0.37 | 4.61 | 0.034 | 1.65 | 0.092 | -24.8 | 0.023 | -11.6 | 0.37 |
| C4 | α1 | 2.2 ± 0.4 | 2.1 ± 0.48 | 2.05 | 0.15 | 2.32 | 0.013 | -36.5 | 0.00063 | -15.4 | 0.23 |
| T8 | δ | 3.6 ± 0.49 | 3.4 ± 0.47 | 5.04 | 0.027 | 1.34 | 0.21 | -31.4 | 0.0037 | -34.5 | 0.0061 |
| | θ | 2.7 ± 0.38 | 2.4 ± 0.39 | 17.2 | 6.3e-05 | 2.32 | 0.013 | -33.5 | 0.0018 | -39.6 | 0.0015 |
| | α1 | 2.4 ± 0.41 | 2.1 ± 0.42 | 15 | 0.00017 | 3.38 | 0.00043 | -26.4 | 0.015 | -31.8 | 0.012 |
| | α2 | 2.2 ± 0.38 | 1.9 ± 0.4 | 19.5 | 2.2e-05 | 2.99 | 0.0015 | -7.52 | 0.5 | -12.6 | 0.33 |
| | β1 | 1.9 ± 0.33 | 1.6 ± 0.35 | 13.6 | 0.00035 | 2.16 | 0.021 | -8.49 | 0.44 | -22.1 | 0.085 |
| | β2 | 1.7 ± 0.33 | 1.4 ± 0.35 | 12 | 0.00073 | 2.15 | 0.022 | -14.4 | 0.19 | -24.6 | 0.054 |
| | β3 | 1.5 ± 0.35 | 1.3 ± 0.34 | 12.9 | 0.00049 | 1.6 | 0.11 | -12.7 | 0.25 | -26.7 | 0.036 |
| | β4 | 1.3 ± 0.38 | 1.1 ± 0.35 | 10.7 | 0.0014 | 1.23 | 0.28 | -19.8 | 0.071 | -35 | 0.0053 |
| | γ1 | 1.2 ± 0.43 | 0.91 ± 0.36 | 10.2 | 0.0018 | 0.885 | 0.56 | -20.1 | 0.067 | -30.8 | 0.015 |
| | γ2 | 1 ± 0.46 | 0.74 ± 0.38 | 8.25 | 0.0048 | 0.663 | 0.77 | -14.7 | 0.18 | -28.5 | 0.025 |
| | γ3 | 0.82 ± 0.49 | 0.59 ± 0.4 | 6.87 | 0.0099 | 0.51 | 0.89 | -10.7 | 0.33 | -26.8 | 0.035 |
| | γ4 | 0.7 ± 0.49 | 0.48 ± 0.41 | 6.2 | 0.014 | 0.523 | 0.88 | -10.9 | 0.32 | -22.7 | 0.076 |
| FC4 | α1 | 2.1 ± 0.42 | 1.9 ± 0.47 | 0.481 | 0.49 | 2.68 | 0.0042 | -18.4 | 0.093 | -31.4 | 0.013 |
| Ft10 | β3 | 1.1 ± 0.38 | 0.94 ± 0.38 | 3.41 | 0.067 | 2.11 | 0.024 | -19.9 | 0.069 | -8.43 | 0.51 |
| | β4 | 0.93 ± 0.4 | 0.79 ± 0.38 | 2.36 | 0.13 | 2.32 | 0.013 | -28 | 0.0099 | -17 | 0.19 |
| | γ1 | 0.78 ± 0.42 | 0.62 ± 0.4 | 2.64 | 0.11 | 2.22 | 0.017 | -30.2 | 0.0053 | -12.9 | 0.32 |
| F8 | θ | 2.4 ± 0.45 | 2.1 ± 0.4 | 6.77 | 0.01 | 1.56 | 0.12 | -28.7 | 0.0081 | -34 | 0.0068 |
| | α1 | 2.1 ± 0.43 | 1.8 ± 0.4 | 6.72 | 0.011 | 2.39 | 0.01 | -22.8 | 0.037 | -24.8 | 0.052 |
| | α2 | 1.9 ± 0.42 | 1.6 ± 0.36 | 7.02 | 0.0091 | 1.87 | 0.05 | -11.4 | 0.3 | -12.3 | 0.34 |
| | β1 | 1.6 ± 0.41 | 1.4 ± 0.34 | 4.4 | 0.038 | 1.5 | 0.14 | -10.4 | 0.35 | -16.3 | 0.21 |
| | β3 | 1.3 ± 0.42 | 1 ± 0.34 | 5.22 | 0.024 | 1.37 | 0.2 | -9.84 | 0.37 | -20.4 | 0.11 |
| | β4 | 1.1 ± 0.45 | 0.87 ± 0.34 | 6.53 | 0.012 | 1.3 | 0.23 | -15.5 | 0.16 | -23.5 | 0.066 |
| | γ1 | 0.94 ± 0.49 | 0.7 ± 0.35 | 5.53 | 0.02 | 1.2 | 0.29 | -15.3 | 0.16 | -15.9 | 0.22 |
| | γ2 | 0.78 ± 0.52 | 0.55 ± 0.37 | 4.58 | 0.034 | 1.2 | 0.29 | -13.5 | 0.22 | -15.3 | 0.23 |
| F4 | α1 | 2.1 ± 0.53 | 1.8 ± 0.41 | 4.57 | 0.035 | 1.51 | 0.14 | -21.6 | 0.049 | -29.1 | 0.022 |
| | α2 | 1.8 ± 0.5 | 1.6 ± 0.37 | 4.73 | 0.032 | 0.989 | 0.46 | -13.6 | 0.22 | -16.5 | 0.2 |
| | β4 | 1.1 ± 0.49 | 0.87 ± 0.37 | 4.6 | 0.034 | 0.547 | 0.87 | -20.6 | 0.06 | -30 | 0.018 |

**Table S3.** Comparison of **Relative Spectral Power** (log10) between Boys and Girls during **Eyes-Closed** Condition

(green) Significant Sex Factor — (red) Significant Age Factor

| Eyes Closed | Relative Spectral Power (log10) | | | ANOVA | | | | Pearson Correlation with Age (%) | | | |
|---|---|---|---|---|---|---|---|---|---|---|---|
| | | Boys | Girls | Factor: SEX | | Factor: AGE | | Boys | | Girls | |
| EEG | Freq. | Mean ± Std | Mean ± Std | F | p | F | p | Corr. Coeff | p | Corr. Coeff | p |
| AF4 | θ | -2.6 ± 0.31 | -2.7 ± 0.28 | 5.4 | 0.022 | 1.06 | 0.4 | 6.72 | 0.54 | -17.5 | 0.17 |
| AF4 | α2 | -3 ± 0.41 | -3.2 ± 0.36 | 6.5 | 0.012 | 1.29 | 0.24 | 33.7 | 0.0017 | 20.8 | 0.11 |
| F3 | δ | -1.6 ± 0.06 | -1.6 ± 0.064 | 4.24 | 0.042 | 2.32 | 0.013 | -39.6 | 0.00019 | -27.2 | 0.033 |
| Ft9 | α2 | -3 ± 0.45 | -3.1 ± 0.42 | 2.67 | 0.1 | 2.27 | 0.015 | 45.8 | 1.2e-05 | 34.2 | 0.0065 |
| CP3 | α2 | -2.7 ± 0.55 | -2.9 ± 0.51 | 4.78 | 0.031 | 1.09 | 0.38 | 30.2 | 0.0052 | 38.5 | 0.002 |
| T7 | θ | -2.5 ± 0.31 | -2.7 ± 0.31 | 8.52 | 0.0042 | 0.848 | 0.59 | 13.5 | 0.22 | -7.59 | 0.56 |
| | α1 | -2.5 ± 0.44 | -2.7 ± 0.49 | 5.92 | 0.016 | 1.17 | 0.32 | 14.3 | 0.2 | 10.8 | 0.4 |
| | α2 | -2.8 ± 0.54 | -2.9 ± 0.5 | 10.9 | 0.0012 | 1.75 | 0.071 | 41.2 | 9.9e-05 | 33.6 | 0.0075 |
| | β1 | -3.3 ± 0.41 | -3.4 ± 0.37 | 5.48 | 0.021 | 1.4 | 0.18 | 41.8 | 7.7e-05 | 24.3 | 0.057 |
| Tp7 | α2 | -2.8 ± 0.57 | -2.9 ± 0.5 | 8.17 | 0.005 | 1.74 | 0.073 | 33.1 | 0.0021 | 51.1 | 2.2e-05 |
| P3 | δ | -1.6 ± 0.092 | -1.7 ± 0.099 | 0.399 | 0.53 | 2.18 | 0.019 | -40.2 | 0.00015 | -38 | 0.0023 |
| | α2 | -2.6 ± 0.53 | -2.8 ± 0.49 | 5.23 | 0.024 | 1.48 | 0.15 | 28.1 | 0.0096 | 34 | 0.0069 |
| TP9 | δ | -1.6 ± 0.091 | -1.7 ± 0.089 | 0.0229 | 0.88 | 2.3 | 0.014 | -42.2 | 6.2e-05 | -27 | 0.034 |
| | θ | -2.5 ± 0.31 | -2.6 ± 0.32 | 4.76 | 0.031 | 0.648 | 0.78 | 10.3 | 0.35 | 2.96 | 0.82 |
| | α2 | -2.7 ± 0.55 | -2.8 ± 0.53 | 8.67 | 0.0039 | 1.7 | 0.081 | 42 | 6.9e-05 | 40.4 | 0.0011 |
| PO7 | δ | -1.6 ± 0.099 | -1.6 ± 0.1 | 0.112 | 0.74 | 2.39 | 0.01 | -46.1 | 1e-05 | -35.8 | 0.0043 |
| | θ | -2.4 ± 0.29 | -2.6 ± 0.32 | 5.35 | 0.022 | 0.89 | 0.55 | -6.09 | 0.58 | 4.55 | 0.73 |
| | α1 | -2.3 ± 0.47 | -2.5 ± 0.52 | 5.05 | 0.026 | 0.845 | 0.6 | 1.6 | 0.89 | 23.9 | 0.062 |
| | α2 | -2.5 ± 0.56 | -2.7 ± 0.55 | 10.1 | 0.0019 | 1.47 | 0.15 | 36.7 | 0.0006 | 44.9 | 0.00025 |
| | γ1 | -4.1 ± 0.29 | -4.1 ± 0.33 | 0.124 | 0.73 | 2.39 | 0.01 | 19.3 | 0.079 | 18.8 | 0.14 |
| | γ2 | -4.2 ± 0.31 | -4.2 ± 0.34 | 0.128 | 0.72 | 2.71 | 0.0037 | 16.2 | 0.14 | 20.8 | 0.11 |
| | γ3 | -4.5 ± 0.34 | -4.5 ± 0.35 | 0.308 | 0.58 | 2.65 | 0.0045 | 12.7 | 0.25 | 17.5 | 0.17 |
| | γ4 | -4.6 ± 0.36 | -4.6 ± 0.35 | 0.185 | 0.67 | 2.53 | 0.0066 | 11.2 | 0.31 | 16.2 | 0.21 |
| O1 | δ | -1.6 ± 0.11 | -1.7 ± 0.086 | 0.0165 | 0.9 | 2.97 | 0.0017 | -44.3 | 2.4e-05 | -39.3 | 0.0016 |
| | θ | -2.4 ± 0.3 | -2.6 ± 0.32 | 8.32 | 0.0046 | 1.75 | 0.07 | -18.6 | 0.09 | -2.15 | 0.87 |
| | α1 | -2.3 ± 0.48 | -2.5 ± 0.56 | 7.99 | 0.0055 | 1.89 | 0.047 | -8.12 | 0.46 | 10.9 | 0.4 |
| | α2 | -2.5 ± 0.54 | -2.7 ± 0.58 | 14.1 | 0.00027 | 1.92 | 0.044 | 30.9 | 0.0042 | 34 | 0.0069 |
| | β1 | -3.1 ± 0.38 | -3.3 ± 0.4 | 5.52 | 0.02 | 1.7 | 0.082 | 21.9 | 0.046 | 26.3 | 0.039 |
| | γ1 | -4.1 ± 0.29 | -4.1 ± 0.31 | 0.978 | 0.32 | 2.08 | 0.027 | 17.6 | 0.11 | 23.3 | 0.069 |
| | γ2 | -4.2 ± 0.31 | -4.3 ± 0.32 | 0.717 | 0.4 | 2.4 | 0.01 | 16.7 | 0.13 | 24.1 | 0.059 |
| | γ3 | -4.5 ± 0.35 | -4.5 ± 0.32 | 0.668 | 0.42 | 2.49 | 0.0076 | 14.9 | 0.18 | 19.3 | 0.13 |
| | γ4 | -4.6 ± 0.37 | -4.6 ± 0.33 | 0.498 | 0.48 | 2.58 | 0.0058 | 13.8 | 0.21 | 18.4 | 0.15 |
| O2 | δ | -1.6 ± 0.11 | -1.7 ± 0.097 | 0.0046 | 0.95 | 3.08 | 0.0011 | -44.6 | 2.1e-05 | -49.7 | 4e-05 |
| | θ | -2.4 ± 0.3 | -2.5 ± 0.32 | 5.52 | 0.02 | 1.69 | 0.083 | -14.2 | 0.2 | -0.121 | 0.99 |
| | α1 | -2.3 ± 0.48 | -2.5 ± 0.54 | 4.89 | 0.029 | 1.52 | 0.13 | -3.47 | 0.75 | 19 | 0.14 |
| | α2 | -2.5 ± 0.55 | -2.7 ± 0.57 | 11.2 | 0.0011 | 1.99 | 0.035 | 35.5 | 0.00092 | 38.2 | 0.0022 |
| | β1 | -3.2 ± 0.38 | -3.2 ± 0.36 | 5.57 | 0.02 | 2.4 | 0.01 | 25.9 | 0.017 | 31.3 | 0.013 |
| | β2 | -3.4 ± 0.33 | -3.5 ± 0.37 | 2.46 | 0.12 | 2.36 | 0.011 | 26.4 | 0.015 | 31.7 | 0.012 |
| | β3 | -3.6 ± 0.34 | -3.7 ± 0.36 | 2.31 | 0.13 | 2.5 | 0.0072 | 35 | 0.0011 | 33.1 | 0.0086 |
| | β4 | -3.9 ± 0.31 | -3.9 ± 0.33 | 0.438 | 0.51 | 2.08 | 0.027 | 27.1 | 0.013 | 22.7 | 0.077 |
| | γ1 | -4.1 ± 0.3 | -4.1 ± 0.31 | 0.252 | 0.62 | 2.18 | 0.02 | 22.7 | 0.038 | 24.9 | 0.051 |
| | γ2 | -4.3 ± 0.32 | -4.2 ± 0.33 | 0.0048 | 0.94 | 2.21 | 0.018 | 21.5 | 0.049 | 23.6 | 0.065 |
| | γ3 | -4.5 ± 0.37 | -4.4 ± 0.34 | 0.0601 | 0.81 | 1.97 | 0.038 | 17.2 | 0.12 | 20.1 | 0.12 |
| | γ4 | -4.6 ± 0.39 | -4.6 ± 0.35 | 0.121 | 0.73 | 2.23 | 0.017 | 16.7 | 0.13 | 19 | 0.14 |
| PO8 | δ | -1.7 ± 0.12 | -1.6 ± 0.091 | 0.987 | 0.32 | 2.13 | 0.023 | -44.8 | 1.9e-05 | -52.4 | 1.2e-05 |
| | α2 | -2.5 ± 0.53 | -2.6 ± 0.54 | 7.54 | 0.007 | 1.48 | 0.15 | 37.4 | 0.00045 | 38.2 | 0.0022 |
| | γ1 | -4 ± 0.28 | -4 ± 0.31 | 0.0201 | 0.89 | 1.97 | 0.037 | 17.1 | 0.12 | 17.7 | 0.17 |
| | γ2 | -4.2 ± 0.32 | -4.2 ± 0.31 | 0.11 | 0.74 | 2.04 | 0.03 | 15.6 | 0.16 | 20.5 | 0.11 |
| | γ3 | -4.4 ± 0.37 | -4.4 ± 0.32 | 0.0026 | 0.96 | 2.27 | 0.015 | 14.8 | 0.18 | 20.6 | 0.11 |
| | γ4 | -4.6 ± 0.38 | -4.5 ± 0.33 | 0.033 | 0.86 | 2.26 | 0.015 | 12.1 | 0.27 | 19.6 | 0.13 |
| P4 | θ | -2.4 ± 0.35 | -2.6 ± 0.32 | 5.79 | 0.018 | 1.57 | 0.12 | -2.36 | 0.83 | -10.1 | 0.44 |
| | α2 | -2.6 ± 0.59 | -2.8 ± 0.55 | 7.76 | 0.0062 | 1.57 | 0.11 | 35.4 | 0.00095 | 26.5 | 0.037 |
| CP4 | α2 | -2.7 ± 0.5 | -2.8 ± 0.55 | 4.91 | 0.029 | 1.77 | 0.067 | 30 | 0.0056 | 45.4 | 0.00021 |
| | γ3 | -4.4 ± 0.38 | -4.4 ± 0.37 | 0.337 | 0.56 | 1.99 | 0.035 | 16.2 | 0.14 | 15.3 | 0.23 |
| | γ4 | -4.6 ± 0.38 | -4.6 ± 0.37 | 0.0924 | 0.76 | 2.2 | 0.019 | 18.6 | 0.09 | 15.4 | 0.23 |
| TP10 | θ | -2.5 ± 0.31 | -2.6 ± 0.32 | 5.19 | 0.024 | 0.776 | 0.66 | -1.74 | 0.87 | 1.42 | 0.91 |
| | α1 | -2.4 ± 0.46 | -2.6 ± 0.5 | 4.42 | 0.038 | 1.16 | 0.32 | 6.49 | 0.56 | 16.3 | 0.2 |

| | | | | | | | | | | | |
|---|---|---|---|---|---|---|---|---|---|---|---|
| | α2 | -2.6 ± 0.5 | -2.8 ± 0.55 | 9.07 | 0.0032 | 2.27 | 0.015 | 44.3 | 2.4e-05 | 39 | 0.0017 |
| | β3 | -3.7 ± 0.33 | -3.7 ± 0.37 | 1.67 | 0.2 | 2.17 | 0.021 | 40.1 | 0.00016 | 30.1 | 0.018 |
| | β4 | -3.9 ± 0.3 | -3.9 ± 0.34 | 0.506 | 0.48 | 2.16 | 0.021 | 28 | 0.01 | 21 | 0.1 |
| | γ2 | -4.2 ± 0.32 | -4.2 ± 0.36 | 0.047 | 0.83 | 1.98 | 0.037 | 19 | 0.084 | 15.7 | 0.22 |
| TP8 | θ | -2.5 ± 0.35 | -2.7 ± 0.37 | 6.64 | 0.011 | 1 | 0.45 | -5.71 | 0.61 | -4.28 | 0.74 |
| | α1 | -2.5 ± 0.51 | -2.7 ± 0.54 | 5.07 | 0.026 | 1.18 | 0.31 | 3.68 | 0.74 | 11.1 | 0.39 |
| | α2 | -2.7 ± 0.56 | -2.9 ± 0.54 | 10.3 | 0.0017 | 1.66 | 0.091 | 31.7 | 0.0033 | 30.3 | 0.017 |
| | β1 | -3.3 ± 0.42 | -3.4 ± 0.44 | 4.59 | 0.034 | 1.7 | 0.082 | 25.5 | 0.019 | 17 | 0.19 |
| C4 | θ | -2.5 ± 0.31 | -2.7 ± 0.35 | 5.78 | 0.018 | 1.79 | 0.064 | 0.629 | 0.95 | -9.4 | 0.47 |
| | α2 | -2.9 ± 0.48 | -3 ± 0.51 | 5.69 | 0.019 | 1.37 | 0.2 | 32.3 | 0.0027 | 25.2 | 0.049 |
| T8 | δ | -1.6 ± 0.083 | -1.7 ± 0.066 | 0.303 | 0.58 | 2.04 | 0.031 | -44.6 | 2.2e-05 | -39.5 | 0.0015 |
| | α2 | -2.8 ± 0.52 | -3 ± 0.5 | 5.78 | 0.018 | 1.45 | 0.16 | 35.7 | 0.00085 | 31.8 | 0.012 |
| T10 | θ | -2.6 ± 0.27 | -2.7 ± 0.31 | 2.07 | 0.15 | 2.04 | 0.03 | 6.65 | 0.55 | -11.8 | 0.36 |
| | α2 | -2.9 ± 0.45 | -3 ± 0.47 | 5.77 | 0.018 | 1.75 | 0.07 | 43.8 | 3.1e-05 | 30.2 | 0.017 |
| Ft10 | δ | -1.6 ± 0.065 | -1.6 ± 0.06 | 0.586 | 0.45 | 1.99 | 0.035 | -33.9 | 0.0016 | -37.8 | 0.0025 |
| F4 | δ | -1.6 ± 0.067 | -1.6 ± 0.067 | 0.0435 | 0.84 | 1.97 | 0.037 | -48.1 | 3.6e-06 | -38.1 | 0.0023 |

**Table S4.** Comparison of **Relative Spectral Power** (log10) between Boys and Girls during **Eyes-Open** Condition

Significant Sex Factor  Significant Age Factor

| Eyes Open | Relative Spectral Power (log10) | | | ANOVA | | | | Pearson Correlation with Age (%) | | | |
|---|---|---|---|---|---|---|---|---|---|---|---|
| | | Boys | Girls | Factor: SEX | | Factor: AGE | | Boys | | Girls | |
| EEG | Freq. | Mean ± Std | Mean ± Std | F | p | F | p | Corr. Coeff | p | Corr. Coeff | p |
| AF4 | θ | -2.6 ± 0.29 | -2.7 ± 0.26 | 4.89 | 0.029 | 0.811 | 0.63 | 9.96 | 0.37 | -15.3 | 0.24 |
| | α1 | -2.9 ± 0.38 | -3.1 ± 0.29 | 4.46 | 0.037 | 0.809 | 0.63 | 8.81 | 0.43 | -1.64 | 0.9 |
| F7 | θ | -2.6 ± 0.32 | -2.7 ± 0.26 | 4.74 | 0.032 | 1.01 | 0.45 | 18 | 0.1 | 1.93 | 0.88 |
| C3 | θ | -2.5 ± 0.32 | -2.6 ± 0.32 | 4.85 | 0.03 | 1.18 | 0.31 | 0.924 | 0.93 | 5.36 | 0.68 |
| CP3 | θ | -2.5 ± 0.32 | -2.6 ± 0.28 | 5.03 | 0.027 | 1.02 | 0.43 | 4.09 | 0.71 | 2.57 | 0.84 |
| T7 | θ | -2.5 ± 0.32 | -2.7 ± 0.28 | 9.76 | 0.0022 | 0.686 | 0.75 | 10.7 | 0.33 | -1.39 | 0.91 |
| | α1 | -2.7 ± 0.4 | -2.9 ± 0.39 | 7.73 | 0.0063 | 1.2 | 0.3 | 16.4 | 0.14 | 6.13 | 0.64 |
| | α2 | -3 ± 0.47 | -3.1 ± 0.41 | 5.78 | 0.018 | 1.24 | 0.27 | 30.1 | 0.0055 | 24.4 | 0.056 |
| Tp7 | θ | -2.5 ± 0.35 | -2.7 ± 0.28 | 6.69 | 0.011 | 0.461 | 0.92 | 7.39 | 0.5 | 8.3 | 0.52 |
| | α1 | -2.7 ± 0.45 | -2.9 ± 0.4 | 4.7 | 0.032 | 0.944 | 0.5 | 10.4 | 0.35 | 18.9 | 0.14 |
| | α2 | -3 ± 0.51 | -3.1 ± 0.43 | 4.37 | 0.039 | 1.21 | 0.29 | 23.7 | 0.03 | 33.4 | 0.008 |
| P3 | θ | -2.4 ± 0.31 | -2.6 ± 0.28 | 5.42 | 0.022 | 1.35 | 0.2 | -5.27 | 0.63 | -9.94 | 0.44 |
| TP9 | θ | -2.4 ± 0.3 | -2.6 ± 0.28 | 6.9 | 0.0098 | 0.605 | 0.82 | 9.21 | 0.4 | 7.59 | 0.56 |
| | α1 | -2.7 ± 0.42 | -2.8 ± 0.39 | 5.48 | 0.021 | 1.04 | 0.42 | 13 | 0.24 | 16.7 | 0.19 |
| | α2 | -2.9 ± 0.48 | -3 ± 0.42 | 4.75 | 0.031 | 1.2 | 0.3 | 30.5 | 0.0047 | 29.9 | 0.018 |
| PO7 | θ | -2.4 ± 0.3 | -2.5 ± 0.3 | 9.24 | 0.0029 | 0.88 | 0.56 | -1.84 | 0.87 | 5.82 | 0.65 |
| | α1 | -2.6 ± 0.41 | -2.7 ± 0.4 | 7.59 | 0.0068 | 0.976 | 0.47 | 4.23 | 0.7 | 17 | 0.19 |
| | α2 | -2.8 ± 0.46 | -2.9 ± 0.42 | 5.41 | 0.022 | 0.855 | 0.59 | 24.6 | 0.024 | 32 | 0.011 |
| O1 | δ | -1.6 ± 0.082 | -1.6 ± 0.074 | 4.95 | 0.028 | 1.84 | 0.054 | -46.9 | 6.7e-0 | -29 | 0.022 |
| | θ | -2.4 ± 0.29 | -2.6 ± 0.33 | 12.4 | 0.00061 | 1.6 | 0.11 | -15.9 | 0.15 | 0.154 | 0.99 |
| | α1 | -2.5 ± 0.43 | -2.8 ± 0.44 | 12.3 | 0.00064 | 2.54 | 0.0064 | -7.49 | 0.5 | 4.86 | 0.71 |
| | α2 | -2.8 ± 0.47 | -2.9 ± 0.44 | 10.4 | 0.0016 | 2.27 | 0.015 | 15.1 | 0.17 | 24.1 | 0.059 |
| O2 | θ | -2.4 ± 0.3 | -2.5 ± 0.3 | 8.97 | 0.0033 | 1.8 | 0.061 | -9.81 | 0.37 | 8.78 | 0.5 |
| | α1 | -2.6 ± 0.43 | -2.7 ± 0.4 | 7.89 | 0.0058 | 1.97 | 0.038 | -0.73 | 0.95 | 14.8 | 0.25 |
| | α2 | -2.8 ± 0.47 | -2.9 ± 0.43 | 7.9 | 0.0058 | 2.06 | 0.028 | 18.5 | 0.092 | 30 | 0.018 |
| | β1 | -3.2 ± 0.4 | -3.2 ± 0.39 | 2.26 | 0.14 | 2.17 | 0.02 | 24.3 | 0.026 | 28.9 | 0.023 |
| | β2 | -3.4 ± 0.37 | -3.4 ± 0.38 | 1.34 | 0.25 | 2.01 | 0.033 | 23.3 | 0.033 | 23.2 | 0.07 |
| | β3 | -3.6 ± 0.36 | -3.6 ± 0.38 | 1.36 | 0.25 | 1.96 | 0.038 | 26.6 | 0.014 | 24.1 | 0.059 |
| PO8 | θ | -2.4 ± 0.27 | -2.5 ± 0.28 | 5.3 | 0.023 | 1.11 | 0.36 | -15.6 | 0.16 | -3.56 | 0.78 |
| | α1 | -2.5 ± 0.41 | -2.7 ± 0.37 | 4.41 | 0.038 | 1.29 | 0.24 | -0.998 | 0.93 | 5.67 | 0.66 |
| | α2 | -2.7 ± 0.43 | -2.9 ± 0.41 | 5.6 | 0.02 | 0.993 | 0.46 | 21.6 | 0.049 | 27.9 | 0.028 |
| P4 | θ | -2.4 ± 0.35 | -2.6 ± 0.29 | 7.26 | 0.0081 | 1.65 | 0.094 | 0.661 | 0.95 | -10.8 | 0.4 |
| | α1 | -2.6 ± 0.48 | -2.8 ± 0.41 | 5.65 | 0.019 | 1.55 | 0.12 | 6.83 | 0.54 | 1.14 | 0.93 |
| | α2 | -2.9 ± 0.51 | -3 ± 0.44 | 5.77 | 0.018 | 1.29 | 0.24 | 23 | 0.035 | 15.5 | 0.23 |
| TP10 | δ | -1.6 ± 0.071 | -1.6 ± 0.075 | 5.49 | 0.021 | 1.18 | 0.31 | -26.9 | 0.013 | -26.3 | 0.039 |
| | θ | -2.4 ± 0.3 | -2.6 ± 0.3 | 7.92 | 0.0057 | 0.907 | 0.54 | -2.93 | 0.79 | 3.04 | 0.81 |
| | α1 | -2.7 ± 0.41 | -2.8 ± 0.4 | 6.26 | 0.014 | 1.33 | 0.22 | 9.11 | 0.41 | 10.4 | 0.42 |
| | α2 | -2.9 ± 0.42 | -3 ± 0.45 | 5.92 | 0.016 | 1.5 | 0.14 | 27.5 | 0.011 | 30.3 | 0.016 |
| TP8 | θ | -2.5 ± 0.33 | -2.6 ± 0.35 | 6.98 | 0.0094 | 1.07 | 0.39 | -6.25 | 0.57 | -6.45 | 0.62 |
| | α1 | -2.7 ± 0.46 | -2.9 ± 0.46 | 7.04 | 0.0091 | 1.36 | 0.2 | 3.28 | 0.77 | 3.91 | 0.76 |
| | α2 | -2.9 ± 0.5 | -3.1 ± 0.47 | 6.1 | 0.015 | 1.4 | 0.18 | 16.7 | 0.13 | 16.4 | 0.2 |
| C4 | θ | -2.5 ± 0.31 | -2.7 ± 0.34 | 5.78 | 0.018 | 1.72 | 0.076 | -1.43 | 0.9 | -6.02 | 0.64 |
| | α1 | -2.8 ± 0.43 | -2.9 ± 0.45 | 4.52 | 0.036 | 1.56 | 0.12 | 5.83 | 0.6 | -0.092 | 0.99 |
| | α2 | -3 ± 0.46 | -3.1 ± 0.46 | 4.75 | 0.031 | 1.43 | 0.17 | 17.7 | 0.11 | 16.3 | 0.21 |
| T8 | θ | -2.5 ± 0.34 | -2.7 ± 0.28 | 4.4 | 0.038 | 1.4 | 0.18 | -3.43 | 0.76 | -3.69 | 0.78 |

**Table S5.** Comparison of boys and girls absolute power (top row) and relative power (bottom row) asymmetry during eyes closed (left column) and eyes open (right column) conditions.

EYES CLOSED ABSOLUTE SPECTA

| Hemispheric EEG Pairs | Frq Band | Boys Log (Left - Right EEG) | | Girls Log (Left - Right EEG) | | ANOVA | |
|---|---|---|---|---|---|---|---|
| | | Mean | Std | Mean | Std | F | p |
| P3 - P4 | α1 | -0.067 ± | 0.31 | 0.058 | 0.31 | 6.32 | 0.013 |
| | α2 | -0.069 ± | 0.31 | 0.053 | 0.32 | 5.9 | 0.016 |
| | β1 | -0.062 ± | 0.27 | 0.069 | 0.32 | 7.83 | 0.006 |

EYES OPEN ABSOLUTE SPECTA

| Hemispheric EEG Pairs | Frq Band | Boys Log (Left - Right EEG) | | Girls Log (Left - Right EEG) | | ANOVA | |
|---|---|---|---|---|---|---|---|
| | | Mean | Std | Mean | Std | F | p |
| P3 - P4 | α1 | -0.063 ± | 0.29 | 0.061 ± | 0.31 | 6.6 | 0.011 |
| | α2 | -0.058 ± | 0.29 | 0.056 ± | 0.31 | 5.5 | 0.02 |
| | β1 | -0.06 ± | 0.27 | 0.06 ± | 0.34 | 6.1 | 0.015 |
| | β2 | -0.052 ± | 0.27 | 0.071 ± | 0.32 | 6.8 | 0.01 |

EYES CLOSED RELATIVE SPECTRA ASYMMETRY

| Hemispheric EEG Pairs | Frq Band | Boys Log (Left - Right EEG) | | Girls Log (Left - Right EEG) | | ANOVA | |
|---|---|---|---|---|---|---|---|
| | | Mean | Std | Mean | Std | F | p |
| AF3 - AF4 | α2 | -0.05 ± | 0.28 | 0.067 ± | 0.23 | 8.2 | 0.0048 |
| | γ2 | -0.03 ± | 0.21 | 0.04 ± | 0.17 | 5.06 | 0.026 |
| T7 - T8 | γ2 | 0.066 ± | 0.20 | -0.009± | 0.18 | 5.96 | 0.016 |
| | γ3 | 0.054± | 0.18 | -0.014 ± | 0.18 | 5.56 | 0.020 |
| | γ4 | 0.048 ± | 0.18 | -0.022 ± | 0.17 | 6.17 | 0.014 |

EYES OPEN RELATIVE SPECTRA ASYMMETRY

| Hemispheric EEG Pairs | Frq Band | Boys Log (Left - Right EEG) | | Girls Log (Left - Right EEG) | | ANOVA | |
|---|---|---|---|---|---|---|---|
| | | Mean | Std | Mean | Std | F | p |
| AF3 - AF4 | α1 | 0.044 ± | 0.29 | 0.058 ± | 0.23 | 5.9 | 0.017 |
| | α2 | -0.054 ± | 0.28 | 0.043 ± | 0.22 | 5.6 | 0.019 |
| | β4 | -0.053± | 0.26 | 0.042 ± | 0.19 | 6.5 | 0.012 |
| TP9 - TP10 | δ | -0.005 ± | 0.06 | 0.016 ± | 0.056 | 5.2 | 0.023 |
| O1 - O2 | γ3 | 0.023 ± | 0.16 | -0.042 ± | 0.18 | 5.7 | 0.018 |

**Table S6.** Comparison of boys and girls **magnitude coherence** during **Eyes Closed** condition.

Significant Sex Factor — Significant Age Factor

| Eyes Closed | Magnitude Coherence | | | ANOVA | | | | Correlation with Age | | | |
|---|---|---|---|---|---|---|---|---|---|---|---|
| | | Boys | Girls | Factor: SEX | | Factor: AGE | | Boys | | Girls | |
| Lobes | Freq. | Mean ± Std | Mean ± Std | F | p | F | p | Corr. Coeff | p | Corr. Coeff | p |
| L Front - L Temp | δ | 0.465 ± 0.16 | 0.53 ± 0.15 | 4.94 | 0.028 | 0.565 | 0.85 | -23.3 | 0.033 | -7.8 | 0.54 |
| | θ | 0.414 ± 0.133 | 0.48 ± 0.14 | 4.96 | 0.028 | 0.434 | 0.94 | -10.2 | 0.36 | -0.066 | 1 |
| | α1 | 0.426 ± 0.134 | 0.5 ± 0.15 | 6.07 | 0.015 | 0.407 | 0.95 | -8.3 | 0.45 | 11 | 0.39 |
| | α2 | 0.413 ± 0.134 | 0.49 ± 0.15 | 5.62 | 0.019 | 0.672 | 0.76 | -2.34 | 0.83 | 20 | 0.12 |
| | β1 | 0.374 ± 0.121 | 0.46 ± 0.14 | 10.4 | 0.0016 | 0.808 | 0.63 | -5.22 | 0.64 | 16 | 0.22 |
| | β2 | 0.384 ± 0.125 | 0.47 ± 0.14 | 8.76 | 0.0037 | 0.736 | 0.7 | -7.27 | 0.51 | 9.2 | 0.48 |
| | β3 | 0.399 ± 0.136 | 0.48 ± 0.15 | 8.2 | 0.005 | 0.872 | 0.57 | -9.87 | 0.37 | 7.8 | 0.55 |
| | β4 | 0.419 ± 0.141 | 0.5 ± 0.15 | 7.62 | 0.0067 | 0.644 | 0.79 | -15.8 | 0.15 | 6.2 | 0.63 |
| | γ1 | 0.446 ± 0.162 | 0.52 ± 0.15 | 5.78 | 0.018 | 0.677 | 0.76 | -19.2 | 0.08 | 1.4 | 0.91 |
| | γ2 | 0.473 ± 0.176 | 0.55 ± 0.16 | 4.63 | 0.033 | 0.615 | 0.81 | -17.2 | 0.12 | -4.3 | 0.74 |
| L Front - L Par/Occ | δ | 0.363 ± 0.183 | 0.44 ± 0.17 | 5.87 | 0.017 | 0.675 | 0.76 | -8.19 | 0.46 | 2.9 | 0.82 |
| | θ | 0.329 ± 0.147 | 0.39 ± 0.15 | 4.72 | 0.032 | 0.902 | 0.54 | -0.185 | 0.99 | 14 | 0.29 |
| | α1 | 0.352 ± 0.144 | 0.42 ± 0.17 | 6.12 | 0.015 | 0.849 | 0.59 | 2.85 | 0.8 | 18 | 0.15 |
| | α2 | 0.342 ± 0.15 | 0.41 ± 0.16 | 5.4 | 0.022 | 1.37 | 0.2 | 11.5 | 0.3 | 30 | 0.019 |
| | β1 | 0.288 ± 0.129 | 0.37 ± 0.15 | 11.5 | 0.00096 | 1.59 | 0.11 | 5.03 | 0.65 | 26 | 0.042 |
| | β2 | 0.297 ± 0.133 | 0.38 ± 0.15 | 10.5 | 0.0015 | 1.57 | 0.12 | 6.61 | 0.55 | 20 | 0.13 |
| | β3 | 0.306 ± 0.144 | 0.39 ± 0.16 | 8.5 | 0.0042 | 1.59 | 0.11 | 2.89 | 0.79 | 18 | 0.15 |
| | β4 | 0.319 ± 0.155 | 0.41 ± 0.16 | 9.96 | 0.002 | 1.29 | 0.24 | -0.784 | 0.94 | 14 | 0.26 |
| | γ1 | 0.345 ± 0.179 | 0.43 ± 0.17 | 7.26 | 0.0081 | 1.04 | 0.42 | -7.04 | 0.52 | 7.1 | 0.58 |
| | γ2 | 0.376 ± 0.198 | 0.46 ± 0.18 | 5.87 | 0.017 | 0.785 | 0.65 | -7.44 | 0.5 | 1.5 | 0.91 |
| | γ3 | 0.403 ± 0.215 | 0.48 ± 0.18 | 4.36 | 0.039 | 0.579 | 0.84 | -10.2 | 0.36 | -3.3 | 0.8 |
| L Cent - L Temp | δ | 0.55 ± 0.152 | 0.62 ± 0.14 | 7.97 | 0.0056 | 0.633 | 0.8 | -6.74 | 0.54 | -19 | 0.14 |
| | θ | 0.553 ± 0.13 | 0.62 ± 0.15 | 7.08 | 0.0089 | 0.69 | 0.75 | 7.18 | 0.52 | -10 | 0.42 |
| | α1 | 0.578 ± 0.132 | 0.65 ± 0.15 | 10 | 0.002 | 0.731 | 0.71 | -2.53 | 0.82 | 0.6 | 0.96 |
| | α2 | 0.561 ± 0.13 | 0.65 ± 0.15 | 9.9 | 0.0021 | 0.631 | 0.8 | 12.1 | 0.27 | 14 | 0.28 |
| | β1 | 0.509 ± 0.12 | 0.61 ± 0.14 | 19.1 | 2.7e-05 | 0.592 | 0.83 | 14.2 | 0.2 | 7.2 | 0.58 |
| | β2 | 0.517 ± 0.121 | 0.62 ± 0.14 | 18.4 | 3.7e-05 | 0.651 | 0.78 | 11.7 | 0.29 | 2.6 | 0.84 |
| | β3 | 0.516 ± 0.129 | 0.63 ± 0.14 | 18.6 | 3.4e-05 | 0.785 | 0.65 | 9.9 | 0.37 | 1.8 | 0.89 |
| | β4 | 0.521 ± 0.134 | 0.63 ± 0.14 | 16.7 | 8.1e-05 | 0.582 | 0.84 | 1.78 | 0.87 | -1.6 | 0.9 |
| | γ1 | 0.526 ± 0.154 | 0.64 ± 0.14 | 16 | 0.00011 | 0.501 | 0.9 | -3 | 0.79 | -4.2 | 0.75 |
| | γ2 | 0.538 ± 0.17 | 0.65 ± 0.15 | 13.1 | 0.00043 | 0.467 | 0.92 | -2.83 | 0.8 | -10 | 0.42 |
| | γ3 | 0.545 ± 0.186 | 0.65 ± 0.14 | 11.6 | 0.0009 | 0.474 | 0.92 | -6.33 | 0.57 | -16 | 0.2 |
| | γ4 | 0.53 ± 0.183 | 0.63 ± 0.15 | 10.9 | 0.0013 | 0.52 | 0.89 | -7.47 | 0.5 | -18 | 0.16 |
| L Cent - L Par/Occ | δ | 0.52 ± 0.157 | 0.58 ± 0.17 | 5.49 | 0.021 | 0.434 | 0.94 | -8.87 | 0.42 | 2.9 | 0.83 |
| | θ | 0.528 ± 0.13 | 0.58 ± 0.18 | 4.88 | 0.029 | 0.596 | 0.83 | -3.13 | 0.78 | 17 | 0.18 |
| | α1 | 0.545 ± 0.132 | 0.61 ± 0.19 | 7.47 | 0.0072 | 0.978 | 0.47 | -5.48 | 0.62 | 25 | 0.052 |
| | α2 | 0.532 ± 0.137 | 0.6 ± 0.19 | 6.23 | 0.014 | 1.19 | 0.3 | 5.34 | 0.63 | 31 | 0.013 |
| | β1 | 0.475 ± 0.116 | 0.57 ± 0.18 | 15.1 | 0.00017 | 1.14 | 0.34 | 6.75 | 0.54 | 29 | 0.025 |
| | β2 | 0.48 ± 0.119 | 0.57 ± 0.18 | 14.7 | 0.0002 | 1.03 | 0.43 | 5.96 | 0.59 | 24 | 0.057 |
| | β3 | 0.48 ± 0.129 | 0.58 ± 0.19 | 13.9 | 0.0003 | 1.07 | 0.39 | 6.01 | 0.59 | 23 | 0.067 |
| | β4 | 0.482 ± 0.137 | 0.59 ± 0.18 | 15.1 | 0.00017 | 0.865 | 0.58 | 0.124 | 0.99 | 18 | 0.17 |
| | γ1 | 0.494 ± 0.156 | 0.6 ± 0.18 | 13.6 | 0.00034 | 0.68 | 0.75 | -5.84 | 0.6 | 14 | 0.29 |
| | γ2 | 0.513 ± 0.172 | 0.62 ± 0.18 | 11.2 | 0.0011 | 0.551 | 0.86 | -7.38 | 0.5 | 8.3 | 0.52 |
| | γ3 | 0.524 ± 0.187 | 0.63 ± 0.17 | 10.8 | 0.0013 | 0.567 | 0.85 | -6.53 | 0.56 | 0.62 | 0.96 |
| | γ4 | 0.512 ± 0.186 | 0.61 ± 0.17 | 9.05 | 0.0032 | 0.67 | 0.76 | -7.95 | 0.47 | 1 | 0.94 |
| L Temp - L Par/Occ | γ1 | 0.564 ± 0.152 | 0.64 ± 0.15 | 4.77 | 0.031 | 0.443 | 0.93 | -21 | 0.055 | -8.8 | 0.49 |
| | γ2 | 0.572 ± 0.167 | 0.65 ± 0.15 | 4.57 | 0.035 | 0.381 | 0.96 | -22.9 | 0.036 | -13 | 0.3 |
| R Front - R Temp | δ | 0.457 ± 0.151 | 0.52 ± 0.14 | 5.15 | 0.025 | 0.731 | 0.71 | -18.3 | 0.096 | -0.17 | 0.99 |
| | θ | 0.394 ± 0.127 | 0.47 ± 0.13 | 7.69 | 0.0065 | 0.773 | 0.67 | -9.53 | 0.39 | 3.4 | 0.79 |
| | α1 | 0.41 ± 0.127 | 0.49 ± 0.14 | 7.15 | 0.0085 | 0.951 | 0.49 | -6.58 | 0.55 | 13 | 0.32 |
| | α2 | 0.396 ± 0.128 | 0.47 ± 0.13 | 6.39 | 0.013 | 1.37 | 0.19 | 4.84 | 0.66 | 24 | 0.058 |
| | β1 | 0.355 ± 0.11 | 0.45 ± 0.13 | 13.6 | 0.00034 | 1.55 | 0.12 | -3.92 | 0.72 | 21 | 0.1 |
| | β2 | 0.363 ± 0.11 | 0.45 ± 0.13 | 13.2 | 0.00042 | 1.25 | 0.26 | -7.8 | 0.48 | 11 | 0.38 |
| | β3 | 0.373 ± 0.116 | 0.47 ± 0.13 | 12.8 | 0.00051 | 1.1 | 0.37 | -9.61 | 0.38 | 12 | 0.35 |
| | β4 | 0.396 ± 0.128 | 0.48 ± 0.14 | 9.63 | 0.0024 | 1.16 | 0.32 | -13.1 | 0.24 | 11 | 0.38 |
| | γ1 | 0.428 ± 0.145 | 0.51 ± 0.15 | 7.09 | 0.0088 | 0.845 | 0.6 | -15.7 | 0.15 | 7.3 | 0.57 |
| | γ2 | 0.459 ± 0.164 | 0.54 ± 0.15 | 6.17 | 0.014 | 0.726 | 0.71 | -17.8 | 0.11 | 2.9 | 0.83 |

| | | | | | | | | | | | |
|---|---|---|---|---|---|---|---|---|---|---|---|
| | γ3 | 0.485 ± 0.171 | 0.56 ± 0.16 | 4.63 | 0.033 | 0.552 | 0.86 | -18.6 | 0.09 | -1.9 | 0.88 |
| | γ4 | 0.473 ± 0.168 | 0.55 ± 0.16 | 4.95 | 0.028 | 0.677 | 0.76 | -19.5 | 0.076 | -0.72 | 0.96 |
| R Front - R Par/Occ | β1 | 0.271 ± 0.123 | 0.33 ± 0.14 | 4.7 | 0.032 | 1.38 | 0.19 | 12.9 | 0.24 | 16 | 0.21 |
| | β2 | 0.274 ± 0.126 | 0.34 ± 0.14 | 5.75 | 0.018 | 1.14 | 0.34 | 10.1 | 0.36 | 5.3 | 0.68 |
| | β3 | 0.285 ± 0.133 | 0.35 ± 0.14 | 5.48 | 0.021 | 1.18 | 0.31 | 8.52 | 0.44 | 3.3 | 0.8 |
| | β4 | 0.301 ± 0.147 | 0.37 ± 0.15 | 5.36 | 0.022 | 1.08 | 0.38 | 1.8 | 0.87 | 5.3 | 0.68 |
| | γ1 | 0.332 ± 0.17 | 0.41 ± 0.16 | 4.58 | 0.034 | 1.03 | 0.42 | -2.68 | 0.81 | 2.4 | 0.86 |
| R Cent - R Temp | β1 | 0.508 ± 0.11 | 0.59 ± 0.15 | 9.24 | 0.0029 | 0.639 | 0.79 | 1.82 | 0.87 | 13 | 0.33 |
| | β2 | 0.51 ± 0.113 | 0.59 ± 0.15 | 9.59 | 0.0024 | 0.513 | 0.89 | -3.2 | 0.77 | 7.1 | 0.59 |
| | β3 | 0.509 ± 0.118 | 0.59 ± 0.15 | 8.21 | 0.0049 | 0.606 | 0.82 | 0.566 | 0.96 | 8.5 | 0.51 |
| | β4 | 0.515 ± 0.125 | 0.6 ± 0.15 | 8.44 | 0.0044 | 0.527 | 0.88 | -7.89 | 0.48 | 2.9 | 0.82 |
| | γ1 | 0.527 ± 0.142 | 0.61 ± 0.16 | 7.18 | 0.0084 | 0.488 | 0.91 | -10.4 | 0.35 | -0.22 | 0.99 |
| | γ2 | 0.545 ± 0.158 | 0.62 ± 0.17 | 6.46 | 0.012 | 0.474 | 0.92 | -11.7 | 0.29 | -3.5 | 0.79 |
| | γ3 | 0.558 ± 0.169 | 0.63 ± 0.17 | 4.9 | 0.029 | 0.364 | 0.97 | -13.8 | 0.21 | -8 | 0.54 |
| | γ4 | 0.546 ± 0.167 | 0.61 ± 0.17 | 4.41 | 0.038 | 0.439 | 0.94 | -13.9 | 0.21 | -9.5 | 0.46 |
| R Cent - R Par/Occ | β2 | 0.451 ± 0.112 | 0.52 ± 0.18 | 4.54 | 0.035 | 0.475 | 0.92 | -10.3 | 0.35 | 3.8 | 0.77 |
| L Front - R Front | β3 | 0.334 ± 0.144 | 0.35 ± 0.14 | 0.0066 | 0.94 | 1.91 | 0.045 | 0.187 | 0.99 | 23 | 0.079 |
| | β4 | 0.357 ± 0.154 | 0.38 ± 0.15 | 0.0801 | 0.78 | 1.95 | 0.039 | -2.79 | 0.8 | 24 | 0.063 |
| | γ2 | 0.425 ± 0.181 | 0.46 ± 0.17 | 0.398 | 0.53 | 1.41 | 0.18 | -9.98 | 0.37 | 14 | 0.28 |
| L Cent - R Cent | α2 | 0.343 ± 0.171 | 0.39 ± 0.2 | 1.74 | 0.19 | 2.37 | 0.011 | 14.2 | 0.2 | 41 | 0.0011 |
| | β1 | 0.3 ± 0.155 | 0.37 ± 0.19 | 4.49 | 0.036 | 2.18 | 0.02 | 7.61 | 0.49 | 35 | 0.005 |
| | β2 | 0.306 ± 0.159 | 0.37 ± 0.2 | 4.26 | 0.041 | 2.28 | 0.015 | 10.7 | 0.33 | 32 | 0.011 |
| | β3 | 0.322 ± 0.174 | 0.39 ± 0.2 | 3.86 | 0.052 | 2.31 | 0.013 | 8.19 | 0.46 | 30 | 0.019 |
| | β4 | 0.339 ± 0.186 | 0.4 ± 0.2 | 3.51 | 0.063 | 2.26 | 0.016 | 6.6 | 0.55 | 25 | 0.05 |
| | γ1 | 0.375 ± 0.203 | 0.44 ± 0.2 | 3.68 | 0.057 | 2.09 | 0.026 | 5.68 | 0.61 | 17 | 0.18 |
| L Par/Occ - R Par/Occ | β4 | 0.54 ± 0.149 | 0.61 ± 0.19 | 4.86 | 0.029 | 0.499 | 0.9 | -1.69 | 0.88 | -5.5 | 0.67 |
| | γ1 | 0.549 ± 0.165 | 0.62 ± 0.18 | 4.43 | 0.037 | 0.477 | 0.91 | -7.14 | 0.52 | -10 | 0.43 |
| L Front - R Cent | α2 | 0.319 ± 0.145 | 0.34 ± 0.15 | 0.43 | 0.51 | 1.83 | 0.056 | 7.87 | 0.48 | 41 | 0.00099 |
| | β4 | 0.315 ± 0.154 | 0.34 ± 0.16 | 1.2 | 0.28 | 1.85 | 0.053 | -5.83 | 0.6 | 23 | 0.073 |
| L Front - R Temp | α2 | 0.316 ± 0.129 | 0.35 ± 0.14 | 0.5 | 0.48 | 2.46 | 0.0083 | 12 | 0.28 | 44 | 0.00034 |
| | β1 | 0.273 ± 0.115 | 0.31 ± 0.13 | 2.22 | 0.14 | 2.38 | 0.011 | 0.657 | 0.95 | 36 | 0.0038 |
| | β3 | 0.293 ± 0.13 | 0.33 ± 0.14 | 1.54 | 0.22 | 2.1 | 0.025 | -2.1 | 0.85 | 30 | 0.017 |
| | β4 | 0.315 ± 0.145 | 0.36 ± 0.15 | 1.85 | 0.18 | 1.81 | 0.06 | -7.93 | 0.47 | 26 | 0.045 |
| L Front - R Par/Occ | δ | 0.337 ± 0.18 | 0.39 ± 0.16 | 4.48 | 0.036 | 0.998 | 0.45 | -10.2 | 0.36 | 6.3 | 0.63 |
| | α1 | 0.304 ± 0.145 | 0.37 ± 0.15 | 4.9 | 0.029 | 1 | 0.45 | 7.54 | 0.5 | 21 | 0.1 |
| | β1 | 0.246 ± 0.123 | 0.31 ± 0.13 | 6.52 | 0.012 | 1.76 | 0.069 | 5.41 | 0.62 | 2 | 0.091 |
| | β2 | 0.252 ± 0.126 | 0.31 ± 0.14 | 6.07 | 0.015 | 1.38 | 0.19 | 4.48 | 0.69 | 13 | 0.32 |
| | β3 | 0.264 ± 0.136 | 0.32 ± 0.15 | 4.74 | 0.031 | 1.65 | 0.094 | 2.07 | 0.85 | 16 | 0.21 |
| | β4 | 0.278 ± 0.149 | 0.34 ± 0.16 | 5.46 | 0.021 | 1.5 | 0.14 | -2.38 | 0.83 | 14 | 0.29 |
| | γ1 | 0.309 ± 0.171 | 0.37 ± 0.17 | 4.69 | 0.032 | 1.31 | 0.23 | -10.1 | 0.36 | 7.9 | 0.54 |
| L Cent - R Temp | α2 | 0.335 ± 0.163 | 0.4 ± 0.18 | 2.74 | 0.1 | 2.44 | 0.0088 | 17.8 | 0.11 | 43 | 0.00044 |
| | β1 | 0.29 ± 0.139 | 0.37 ± 0.17 | 6.03 | 0.016 | 2.16 | 0.021 | 8.74 | 0.43 | 37 | 0.0028 |
| | β2 | 0.295 ± 0.145 | 0.37 ± 0.16 | 6.27 | 0.014 | 1.86 | 0.052 | 11.3 | 0.31 | 33 | 0.0081 |
| | β3 | 0.312 ± 0.154 | 0.39 ± 0.17 | 5.82 | 0.017 | 2.12 | 0.024 | 7.75 | 0.48 | 32 | 0.012 |
| | β4 | 0.332 ± 0.166 | 0.41 ± 0.17 | 5.6 | 0.02 | 1.95 | 0.04 | 3.03 | 0.78 | 26 | 0.043 |
| | γ1 | 0.365 ± 0.184 | 0.44 ± 0.17 | 5.08 | 0.026 | 1.57 | 0.12 | 0.291 | 0.98 | 17 | 0.18 |
| | γ2 | 0.403 ± 0.202 | 0.48 ± 0.18 | 4.38 | 0.038 | 1.4 | 0.18 | -1.53 | 0.89 | 8.4 | 0.51 |
| L Cent - R Par/Occ | θ | 0.349 ± 0.173 | 0.41 ± 0.18 | 4.75 | 0.031 | 0.785 | 0.65 | 3.72 | 0.74 | 22 | 0.088 |
| | α1 | 0.353 ± 0.18 | 0.44 ± 0.21 | 6.32 | 0.013 | 1.19 | 0.3 | 8.53 | 0.44 | 27 | 0.031 |
| | α2 | 0.349 ± 0.185 | 0.43 ± 0.2 | 5.4 | 0.022 | 1.66 | 0.091 | 16.4 | 0.14 | 33 | 0.0098 |
| | β1 | 0.295 ± 0.156 | 0.4 ± 0.19 | 12.7 | 0.00053 | 1.33 | 0.21 | 8.32 | 0.45 | 27 | 0.033 |
| | β2 | 0.299 ± 0.161 | 0.41 ± 0.19 | 12.2 | 0.00067 | 1.04 | 0.41 | 9.42 | 0.39 | 23 | 0.075 |
| | β3 | 0.316 ± 0.171 | 0.43 ± 0.19 | 11.8 | 0.00083 | 1.15 | 0.33 | 6.82 | 0.54 | 22 | 0.08 |
| | β4 | 0.329 ± 0.181 | 0.44 ± 0.19 | 11.8 | 0.00081 | 1.08 | 0.38 | 2.39 | 0.83 | 21 | 0.11 |
| | γ1 | 0.359 ± 0.198 | 0.47 ± 0.18 | 10.4 | 0.0016 | 0.914 | 0.53 | -1.99 | 0.86 | 13 | 0.3 |
| | γ2 | 0.398 ± 0.214 | 0.5 ± 0.18 | 8.44 | 0.0044 | 0.867 | 0.57 | -4.03 | 0.72 | 7.8 | 0.55 |
| | γ3 | 0.422 ± 0.227 | 0.52 ± 0.19 | 6.15 | 0.015 | 0.807 | 0.63 | -3.84 | 0.73 | -0.033 | 1 |
| | γ4 | 0.414 ± 0.225 | 0.5 ± 0.18 | 5.43 | 0.022 | 0.928 | 0.52 | -4.16 | 0.71 | -1.4 | 0.92 |
| L Temp - R Par/Occ | α2 | 0.46 ± 0.181 | 0.5 ± 0.17 | 0.291 | 0.59 | 1.93 | 0.043 | 14.5 | 0.19 | 25 | 0.047 |
| | β1 | 0.389 ± 0.145 | 0.46 ± 0.16 | 5.21 | 0.024 | 0.984 | 0.46 | -5.55 | 0.62 | 14 | 0.27 |
| | β2 | 0.388 ± 0.153 | 0.46 ± 0.17 | 5.54 | 0.02 | 0.824 | 0.62 | -2.39 | 0.83 | 10 | 0.43 |
| | β3 | 0.403 ± 0.156 | 0.48 ± 0.17 | 5.33 | 0.023 | 0.896 | 0.55 | -3.77 | 0.73 | 7.3 | 0.57 |
| | β4 | 0.402 ± 0.162 | 0.49 ± 0.17 | 7.64 | 0.0066 | 0.683 | 0.75 | -7.74 | 0.48 | 3.6 | 0.78 |
| | γ1 | 0.421 ± 0.181 | 0.51 ± 0.17 | 7.66 | 0.0066 | 0.684 | 0.75 | -13.7 | 0.21 | -0.33 | 0.98 |
| | γ2 | 0.453 ± 0.194 | 0.54 ± 0.17 | 6.26 | 0.014 | 0.636 | 0.79 | -14.6 | 0.19 | -5.6 | 0.67 |

| | | | | | | | | | | | |
|---|---|---|---|---|---|---|---|---|---|---|---|
| R Cent - L Temp | α2 | 0.343 ± 0.163 | 0.38 ± 0.18 | 1.48 | 0.23 | 1.84 | 0.055 | 7.79 | 0.48 | 28 | 0.03 |
| | β1 | 0.293 ± 0.136 | 0.36 ± 0.17 | 6.15 | 0.015 | 1.44 | 0.16 | -3.89 | 0.73 | 17 | 0.2 |
| | β2 | 0.297 ± 0.143 | 0.37 ± 0.18 | 6.15 | 0.015 | 1.49 | 0.14 | -1.07 | 0.92 | 14 | 0.29 |
| | β3 | 0.316 ± 0.153 | 0.38 ± 0.18 | 5.1 | 0.026 | 1.82 | 0.057 | -2.46 | 0.82 | 13 | 0.32 |
| | β4 | 0.334 ± 0.163 | 0.4 ± 0.18 | 5.57 | 0.02 | 1.74 | 0.074 | -0.657 | 0.95 | 9.1 | 0.48 |
| | γ1 | 0.372 ± 0.185 | 0.44 ± 0.19 | 4.76 | 0.031 | 1.65 | 0.093 | -5.56 | 0.62 | 3.9 | 0.77 |
| R Cent - L Par/Occ | β1 | 0.299 ± 0.147 | 0.38 ± 0.21 | 6.89 | 0.0098 | 1.38 | 0.19 | 2.33 | 0.83 | 16 | 0.2 |
| | β2 | 0.298 ± 0.155 | 0.39 ± 0.22 | 6.82 | 0.01 | 1.07 | 0.39 | 2.19 | 0.84 | 15 | 0.25 |
| | β3 | 0.313 ± 0.166 | 0.4 ± 0.22 | 5.44 | 0.021 | 1.23 | 0.28 | 0.509 | 0.96 | 15 | 0.23 |
| | β4 | 0.324 ± 0.176 | 0.41 ± 0.22 | 5.47 | 0.021 | 1.07 | 0.39 | -2.27 | 0.84 | 11 | 0.39 |
| | γ1 | 0.352 ± 0.193 | 0.44 ± 0.22 | 5.29 | 0.023 | 0.998 | 0.45 | -5.38 | 0.63 | 6.6 | 0.61 |
| | γ2 | 0.393 ± 0.207 | 0.48 ± 0.23 | 4.48 | 0.036 | 0.876 | 0.57 | -7.24 | 0.51 | 1.1 | 0.93 |
| R Temp - L Par/Occ | β2 | 0.373 ± 0.151 | 0.45 ± 0.18 | 4.38 | 0.039 | 0.929 | 0.52 | 6.91 | 0.53 | 15 | 0.26 |
| | β4 | 0.389 ± 0.166 | 0.47 ± 0.18 | 5.14 | 0.025 | 0.9 | 0.54 | 1.89 | 0.86 | 9.6 | 0.46 |
| | γ1 | 0.411 ± 0.183 | 0.5 ± 0.18 | 5.22 | 0.024 | 0.795 | 0.64 | -1.54 | 0.89 | 3.1 | 0.81 |
| | γ2 | 0.446 ± 0.197 | 0.53 ± 0.18 | 4.75 | 0.031 | 0.657 | 0.78 | -3.3 | 0.77 | -2.4 | 0.85 |

**Table S7.** Comparison of boys and girls **Magnitude Coherence during Eyes Open** condition.

Significant Sex Factor    Significant Age Factor

| **Eyes Open** | | **Magnitude Coherence** | | **ANOVA** | | | | **Correlation with Age** | | | |
|---|---|---|---|---|---|---|---|---|---|---|---|
| | | **Boys** | **Girls** | **Factor: SEX** | | **Factor: AGE** | | **Boys** | | **Girls** | |
| **Lobes** | **Freq.** | **Mean ± Std** | **Mean ± Std** | **F** | **p** | **F** | **p** | **Corr. Coeff** | **p** | **Corr. Coeff** | **p** |
| L Front - L Temp | δ | 0.47 ± 0.154 | 0.54 ± 0.15 | 5.46 | 0.021 | 0.451 | 0.93 | -19 | 0.083 | 2.1 | 0.87 |
| | θ | 0.42 ± 0.13 | 0.48 ± 0.15 | 5.62 | 0.019 | 0.457 | 0.93 | -9.22 | 0.4 | 1.4 | 0.91 |
| | α1 | 0.417 ± 0.123 | 0.49 ± 0.15 | 6.17 | 0.014 | 0.439 | 0.94 | -3.6 | 0.74 | 9.6 | 0.46 |
| | α2 | 0.403 ± 0.127 | 0.48 ± 0.15 | 6.51 | 0.012 | 0.494 | 0.9 | -4.91 | 0.66 | 17 | 0.19 |
| | β1 | 0.38 ± 0.122 | 0.46 ± 0.14 | 9.84 | 0.0022 | 0.827 | 0.61 | -6.78 | 0.54 | 11 | 0.37 |
| | β2 | 0.391 ± 0.127 | 0.47 ± 0.14 | 8.71 | 0.0038 | 0.759 | 0.68 | -10 | 0.37 | 9.2 | 0.48 |
| | β3 | 0.407 ± 0.136 | 0.48 ± 0.15 | 7.02 | 0.0091 | 0.772 | 0.67 | -8.3 | 0.45 | 9.6 | 0.46 |
| | β4 | 0.428 ± 0.148 | 0.5 ± 0.15 | 4.78 | 0.031 | 0.538 | 0.87 | -15.2 | 0.17 | 8.5 | 0.51 |
| L Front - L Par/Occ | δ | 0.368 ± 0.181 | 0.45 ± 0.17 | 6.66 | 0.011 | 0.583 | 0.84 | -6.78 | 0.54 | 7.2 | 0.58 |
| | θ | 0.332 ± 0.149 | 0.39 ± 0.15 | 5.02 | 0.027 | 0.91 | 0.53 | -0.181 | 0.99 | 16 | 0.22 |
| | α1 | 0.324 ± 0.139 | 0.4 ± 0.16 | 6.52 | 0.012 | 0.945 | 0.5 | 2.77 | 0.8 | 22 | 0.081 |
| | α2 | 0.309 ± 0.137 | 0.38 ± 0.15 | 7.73 | 0.0063 | 1.09 | 0.38 | 2.44 | 0.83 | 29 | 0.024 |
| | β1 | 0.29 ± 0.134 | 0.38 ± 0.15 | 11.8 | 0.0008 | 1.56 | 0.12 | 5.22 | 0.64 | 23 | 0.069 |
| | β2 | 0.296 ± 0.137 | 0.39 ± 0.16 | 11.4 | 0.001 | 1.46 | 0.16 | 4.42 | 0.69 | 20 | 0.12 |
| | β3 | 0.313 ± 0.146 | 0.4 ± 0.16 | 9.66 | 0.0024 | 1.48 | 0.15 | 4.62 | 0.68 | 17 | 0.18 |
| | β4 | 0.329 ± 0.157 | 0.41 ± 0.17 | 7.45 | 0.0073 | 1.22 | 0.28 | -0.458 | 0.97 | 15 | 0.24 |
| | γ1 | 0.34 ± 0.171 | 0.42 ± 0.17 | 6.87 | 0.0099 | 1.17 | 0.32 | -4.3 | 0.7 | 13 | 0.3 |
| | γ2 | 0.361 ± 0.185 | 0.44 ± 0.18 | 5.77 | 0.018 | 0.854 | 0.59 | -5.48 | 0.62 | 4.4 | 0.73 |
| L Cent - L Temp | δ | 0.554 ± 0.158 | 0.63 ± 0.14 | 7.12 | 0.0087 | 0.462 | 0.92 | -3.9 | 0.72 | -11 | 0.4 |
| | θ | 0.547 ± 0.134 | 0.61 ± 0.14 | 7.3 | 0.0079 | 0.648 | 0.78 | 6.53 | 0.56 | -8.8 | 0.5 |
| | α1 | 0.554 ± 0.124 | 0.64 ± 0.15 | 11.5 | 0.00092 | 0.671 | 0.76 | 8.91 | 0.42 | -1.5 | 0.91 |
| | α2 | 0.538 ± 0.127 | 0.63 ± 0.15 | 14 | 0.00028 | 0.551 | 0.86 | 16 | 0.15 | 7.6 | 0.56 |
| | β1 | 0.51 ± 0.12 | 0.61 ± 0.14 | 19.4 | 2.3e-05 | 0.498 | 0.9 | 14.8 | 0.18 | 5.2 | 0.69 |
| | β2 | 0.514 ± 0.124 | 0.62 ± 0.14 | 20.4 | 1.5e-05 | 0.635 | 0.8 | 9.04 | 0.41 | 3.8 | 0.77 |
| | β3 | 0.525 ± 0.131 | 0.63 ± 0.13 | 16.9 | 7.2e-05 | 0.667 | 0.77 | 9.77 | 0.38 | 2.1 | 0.87 |
| | β4 | 0.527 ± 0.139 | 0.63 ± 0.14 | 15 | 0.00018 | 0.632 | 0.8 | 3.3 | 0.77 | 4.8 | 0.71 |
| | γ1 | 0.526 ± 0.152 | 0.63 ± 0.14 | 13.5 | 0.00035 | 0.556 | 0.86 | -1.62 | 0.88 | 0.099 | 0.99 |
| | γ2 | 0.525 ± 0.162 | 0.64 ± 0.14 | 14.7 | 0.0002 | 0.355 | 0.97 | -0.263 | 0.98 | -7.2 | 0.58 |
| | γ3 | 0.533 ± 0.179 | 0.64 ± 0.14 | 12 | 0.00074 | 0.312 | 0.98 | -1.85 | 0.87 | -11 | 0.38 |
| | γ4 | 0.52 ± 0.176 | 0.63 ± 0.14 | 11.3 | 0.0011 | 0.26 | 0.99 | -5.93 | 0.59 | -14 | 0.29 |
| L Cent - L Par/Occ | δ | 0.52 ± 0.166 | 0.58 ± 0.17 | 4.89 | 0.029 | 0.368 | 0.97 | -6.88 | 0.53 | 7.5 | 0.56 |
| | θ | 0.518 ± 0.133 | 0.57 ± 0.18 | 5.63 | 0.019 | 0.423 | 0.94 | -1.89 | 0.86 | 17 | 0.19 |
| | α1 | 0.513 ± 0.125 | 0.59 ± 0.18 | 10.4 | 0.0016 | 0.78 | 0.66 | 1.7 | 0.88 | 23 | 0.07 |
| | α2 | 0.498 ± 0.124 | 0.58 ± 0.19 | 11.3 | 0.001 | 0.902 | 0.54 | 5.27 | 0.63 | 30 | 0.018 |
| | β1 | 0.47 ± 0.123 | 0.57 ± 0.18 | 15.8 | 0.00012 | 0.894 | 0.55 | 7.15 | 0.52 | 27 | 0.036 |
| | β2 | 0.474 ± 0.128 | 0.57 ± 0.18 | 15.1 | 0.00017 | 0.885 | 0.56 | 3.41 | 0.76 | 25 | 0.053 |
| | β3 | 0.484 ± 0.136 | 0.58 ± 0.18 | 13.1 | 0.00043 | 0.886 | 0.56 | 4.39 | 0.69 | 24 | 0.065 |
| | β4 | 0.485 ± 0.141 | 0.59 ± 0.18 | 14.1 | 0.00027 | 0.749 | 0.69 | 2.4 | 0.83 | 23 | 0.07 |
| | γ1 | 0.489 ± 0.152 | 0.59 ± 0.18 | 13.8 | 0.00031 | 0.705 | 0.73 | -2.9 | 0.79 | 18 | 0.16 |
| | γ2 | 0.5 ± 0.164 | 0.61 ± 0.17 | 13 | 0.00046 | 0.59 | 0.83 | -1.01 | 0.93 | 11 | 0.41 |
| | γ3 | 0.512 ± 0.183 | 0.62 ± 0.18 | 10.1 | 0.0019 | 0.506 | 0.9 | -2.08 | 0.85 | 7.4 | 0.57 |
| | γ4 | 0.501 ± 0.179 | 0.6 ± 0.18 | 9.65 | 0.0024 | 0.527 | 0.88 | -4.62 | 0.68 | 4.1 | 0.75 |
| | γ1 | 0.563 ± 0.147 | 0.63 ± 0.14 | 4.55 | 0.035 | 0.355 | 0.97 | -17.7 | 0.11 | -3.8 | 0.77 |
| R Front - R Temp | δ | 0.466 ± 0.158 | 0.53 ± 0.13 | 4.49 | 0.036 | 0.631 | 0.8 | -14.1 | 0.2 | 11 | 0.42 |
| | θ | 0.399 ± 0.129 | 0.47 ± 0.12 | 8.56 | 0.0041 | 0.729 | 0.71 | -8.35 | 0.45 | 11 | 0.41 |
| | α1 | 0.398 ± 0.127 | 0.48 ± 0.13 | 8.3 | 0.0047 | 1.08 | 0.38 | -3.78 | 0.73 | 21 | 0.11 |
| | α2 | 0.381 ± 0.116 | 0.46 ± 0.12 | 9.06 | 0.0032 | 1.08 | 0.39 | -5.2 | 0.64 | 26 | 0.04 |
| | β1 | 0.361 ± 0.113 | 0.45 ± 0.13 | 13.8 | 0.00031 | 1.33 | 0.22 | -8.24 | 0.46 | 25 | 0.053 |
| | β2 | 0.366 ± 0.114 | 0.46 ± 0.13 | 13.2 | 0.00042 | 1.1 | 0.37 | -9.59 | 0.39 | 16 | 0.23 |
| | β3 | 0.384 ± 0.124 | 0.47 ± 0.13 | 9.93 | 0.0021 | 0.822 | 0.62 | -12.7 | 0.25 | 14 | 0.29 |
| | β4 | 0.401 ± 0.133 | 0.48 ± 0.13 | 8.38 | 0.0045 | 0.925 | 0.52 | -14.2 | 0.2 | 14 | 0.28 |
| | γ1 | 0.423 ± 0.144 | 0.5 ± 0.14 | 6.8 | 0.01 | 0.819 | 0.62 | -14.8 | 0.18 | 13 | 0.32 |
| | γ2 | 0.44 ± 0.152 | 0.52 ± 0.15 | 5.74 | 0.018 | 0.566 | 0.85 | -13.4 | 0.22 | 5.4 | 0.68 |
| | γ3 | 0.461 ± 0.166 | 0.54 ± 0.15 | 4.56 | 0.035 | 0.412 | 0.95 | -12.6 | 0.25 | 2.8 | 0.83 |
| R Front - R Par/Occ | β1 | 0.273 ± 0.124 | 0.34 ± 0.14 | 5.52 | 0.02 | 1.2 | 0.29 | 6.76 | 0.54 | 16 | 0.21 |
| | β2 | 0.279 ± 0.132 | 0.34 ± 0.15 | 4.9 | 0.029 | 0.916 | 0.53 | 3.68 | 0.74 | 4.6 | 0.72 |
| R Cent - R Temp | α1 | 0.553 ± 0.13 | 0.62 ± 0.15 | 5.25 | 0.024 | 0.625 | 0.8 | -0.711 | 0.95 | 11 | 0.39 |

| | | | | | | | | | | | |
|---|---|---|---|---|---|---|---|---|---|---|---|
| | α2 | 0.54 ± 0.12 | 0.61 ± 0.15 | 5.22 | 0.024 | 0.481 | 0.91 | 4.73 | 0.67 | 14 | 0.27 |
| | β1 | 0.506 ± 0.113 | 0.59 ± 0.15 | 9.6 | 0.0024 | 0.513 | 0.89 | -1.33 | 0.9 | 14 | 0.28 |
| | β2 | 0.509 ± 0.116 | 0.59 ± 0.15 | 9.62 | 0.0024 | 0.531 | 0.88 | -2.14 | 0.85 | 9 | 0.49 |
| | β3 | 0.519 ± 0.124 | 0.6 ± 0.15 | 8.27 | 0.0048 | 0.466 | 0.92 | -2.37 | 0.83 | 5.3 | 0.68 |
| | β4 | 0.518 ± 0.129 | 0.6 ± 0.15 | 8.02 | 0.0054 | 0.369 | 0.97 | -8.52 | 0.44 | 3.9 | 0.77 |
| | γ1 | 0.526 ± 0.141 | 0.6 ± 0.15 | 6.64 | 0.011 | 0.431 | 0.94 | -11.1 | 0.32 | 2.5 | 0.85 |
| | γ2 | 0.532 ± 0.147 | 0.61 ± 0.16 | 6.92 | 0.0097 | 0.378 | 0.96 | -9 | 0.42 | -1.1 | 0.93 |
| | γ3 | 0.541 ± 0.161 | 0.62 ± 0.16 | 6.07 | 0.015 | 0.372 | 0.96 | -9.76 | 0.38 | -5 | 0.7 |
| | γ4 | 0.531 ± 0.162 | 0.6 ± 0.16 | 4.83 | 0.03 | 0.407 | 0.95 | -9.81 | 0.37 | -6.3 | 0.62 |
| L Cent - R Cent | α2 | 0.32 ± 0.163 | 0.38 ± 0.19 | 2.46 | 0.12 | 1.61 | 0.11 | 7.88 | 0.48 | 39 | 0.0016 |
| | β1 | 0.305 ± 0.165 | 0.37 ± 0.19 | 4.3 | 0.04 | 2.06 | 0.029 | 5.02 | 0.65 | 35 | 0.0057 |
| | β2 | 0.312 ± 0.17 | 0.38 ± 0.19 | 4.54 | 0.035 | 1.86 | 0.052 | 7.18 | 0.52 | 32 | 0.012 |
| L Par/Occ - R Par/Occ | β4 | 0.55 ± 0.148 | 0.62 ± 0.19 | 4.86 | 0.029 | 0.72 | 0.72 | 2.05 | 0.85 | -4.6 | 0.72 |
| | γ1 | 0.547 ± 0.159 | 0.61 ± 0.18 | 4.88 | 0.029 | 0.548 | 0.87 | -2.89 | 0.79 | -8.9 | 0.49 |
| L Front - R Par/Occ | δ | 0.348 ± 0.18 | 0.41 ± 0.17 | 5.02 | 0.027 | 0.771 | 0.67 | -9.94 | 0.37 | 12 | 0.37 |
| | β1 | 0.248 ± 0.123 | 0.31 ± 0.14 | 7.12 | 0.0087 | 1.41 | 0.18 | 1.18 | 0.92 | 18 | 0.16 |
| | β2 | 0.257 ± 0.128 | 0.32 ± 0.14 | 6.27 | 0.014 | 1.28 | 0.25 | -1.31 | 0.91 | 13 | 0.32 |
| | β3 | 0.273 ± 0.139 | 0.33 ± 0.15 | 4.97 | 0.028 | 1.36 | 0.2 | -0.616 | 0.96 | 15 | 0.24 |
| | β4 | 0.283 ± 0.152 | 0.35 ± 0.17 | 5.32 | 0.023 | 1.14 | 0.34 | -5.96 | 0.59 | 13 | 0.32 |
| L Cent - R Temp | α1 | 0.329 ± 0.151 | 0.39 ± 0.17 | 4.49 | 0.036 | 1.56 | 0.12 | 12.5 | 0.26 | 43 | 0.00043 |
| | α2 | 0.307 ± 0.139 | 0.38 ± 0.17 | 5.22 | 0.024 | 1.75 | 0.07 | 7.97 | 0.47 | 46 | 0.00019 |
| | β1 | 0.295 ± 0.146 | 0.37 ± 0.17 | 5.45 | 0.021 | 1.84 | 0.055 | 6.51 | 0.56 | 38 | 0.0025 |
| | β2 | 0.305 ± 0.145 | 0.38 ± 0.16 | 5.47 | 0.021 | 1.65 | 0.093 | 8.82 | 0.42 | 35 | 0.0047 |
| | β3 | 0.326 ± 0.158 | 0.4 ± 0.17 | 5.25 | 0.024 | 1.58 | 0.11 | 7.07 | 0.52 | 32 | 0.012 |
| | β4 | 0.338 ± 0.163 | 0.41 ± 0.17 | 4.4 | 0.038 | 1.58 | 0.11 | 4.85 | 0.66 | 29 | 0.021 |
| | γ2 | 0.387 ± 0.185 | 0.46 ± 0.17 | 4.39 | 0.038 | 1.3 | 0.23 | 3.71 | 0.74 | 13 | 0.32 |
| L Cent - R Par/Occ | θ | 0.365 ± 0.176 | 0.43 ± 0.17 | 5.2 | 0.024 | 0.504 | 0.9 | -2.24 | 0.84 | 22 | 0.092 |
| | α1 | 0.334 ± 0.167 | 0.42 ± 0.19 | 9.03 | 0.0032 | 0.862 | 0.58 | 7.72 | 0.49 | 30 | 0.02 |
| | α2 | 0.315 ± 0.165 | 0.41 ± 0.19 | 9.46 | 0.0026 | 0.949 | 0.5 | 4.25 | 0.7 | 30 | 0.018 |
| | β1 | 0.3 ± 0.159 | 0.41 ± 0.19 | 12.2 | 0.00067 | 0.94 | 0.5 | 4.22 | 0.7 | 26 | 0.045 |
| | β2 | 0.31 ± 0.164 | 0.41 ± 0.19 | 11 | 0.0012 | 0.942 | 0.5 | 4.98 | 0.65 | 22 | 0.082 |
| | β3 | 0.329 ± 0.176 | 0.43 ± 0.19 | 10.7 | 0.0014 | 0.908 | 0.54 | 5.94 | 0.59 | 21 | 0.099 |
| | β4 | 0.336 ± 0.182 | 0.45 ± 0.19 | 10.5 | 0.0016 | 0.899 | 0.54 | 3.26 | 0.77 | 21 | 0.1 |
| | γ1 | 0.355 ± 0.195 | 0.46 ± 0.18 | 9.21 | 0.003 | 0.923 | 0.52 | 0.0962 | 0.99 | 16 | 0.22 |
| | γ2 | 0.377 ± 0.204 | 0.48 ± 0.18 | 8.97 | 0.0033 | 0.832 | 0.61 | 1.78 | 0.87 | 6.6 | 0.61 |
| | γ3 | 0.401 ± 0.22 | 0.5 ± 0.18 | 6.99 | 0.0093 | 0.714 | 0.72 | 0.793 | 0.94 | 0.31 | 0.98 |
| | γ4 | 0.399 ± 0.218 | 0.49 ± 0.18 | 5.44 | 0.021 | 0.673 | 0.76 | -1.32 | 0.91 | -1.1 | 0.93 |
| | β1 | 0.389 ± 0.148 | 0.46 ± 0.16 | 5.66 | 0.019 | 0.772 | 0.67 | -7.94 | 0.47 | 14 | 0.28 |
| | β2 | 0.398 ± 0.153 | 0.47 ± 0.17 | 4.9 | 0.029 | 0.67 | 0.76 | -6.41 | 0.56 | 7.6 | 0.56 |
| | β4 | 0.411 ± 0.166 | 0.49 ± 0.18 | 6.86 | 0.01 | 0.634 | 0.8 | -7.29 | 0.51 | 6.4 | 0.62 |
| | γ1 | 0.417 ± 0.181 | 0.5 ± 0.17 | 6.35 | 0.013 | 0.768 | 0.67 | -10.8 | 0.33 | 1.9 | 0.88 |
| | γ2 | 0.437 ± 0.185 | 0.52 ± 0.17 | 5.64 | 0.019 | 0.654 | 0.78 | -12 | 0.28 | -4.5 | 0.73 |
| | γ3 | 0.448 ± 0.202 | 0.53 ± 0.18 | 4.53 | 0.035 | 0.47 | 0.92 | -13.1 | 0.24 | -8.8 | 0.5 |
| R Cent - L Temp | β1 | 0.297 ± 0.145 | 0.36 ± 0.17 | 5.97 | 0.016 | 1.23 | 0.28 | -8.02 | 0.47 | 19 | 0.14 |
| | β2 | 0.307 ± 0.154 | 0.37 ± 0.18 | 4.97 | 0.028 | 1.12 | 0.36 | -3.86 | 0.73 | 13 | 0.3 |
| | β3 | 0.329 ± 0.164 | 0.38 ± 0.19 | 3.64 | 0.059 | 1.24 | 0.27 | -2.05 | 0.85 | 11 | 0.4 |
| R Cent - L Par/Occ | α1 | 0.337 ± 0.163 | 0.41 ± 0.22 | 4.48 | 0.036 | 1.01 | 0.44 | 5.75 | 0.6 | 19 | 0.15 |
| | α2 | 0.318 ± 0.162 | 0.39 ± 0.22 | 4.76 | 0.031 | 1.03 | 0.42 | 0.67 | 0.95 | 22 | 0.092 |
| | β1 | 0.301 ± 0.156 | 0.39 ± 0.21 | 7.29 | 0.008 | 1.06 | 0.4 | -1.18 | 0.92 | 20 | 0.13 |
| | β2 | 0.307 ± 0.162 | 0.4 ± 0.22 | 6.27 | 0.014 | 0.9 | 0.54 | -0.535 | 0.96 | 15 | 0.24 |
| | β3 | 0.326 ± 0.17 | 0.41 ± 0.22 | 5.38 | 0.022 | 1.01 | 0.44 | 1.11 | 0.92 | 14 | 0.29 |
| | β4 | 0.333 ± 0.182 | 0.42 ± 0.22 | 5.44 | 0.021 | 1.02 | 0.43 | -0.0756 | 0.99 | 12 | 0.36 |
| R Temp - L Par/Occ | β4 | 0.4 ± 0.165 | 0.47 ± 0.18 | 4.48 | 0.036 | 0.85 | 0.59 | 4.79 | 0.67 | 11 | 0.4 |

**Table S8.** Comparison of boys and girls magnitude of **Phase Synchrony** during **Eyes Closed** condition.

Significant Sex Factor    Significant Age Factor

| Eyes Closed | | Phase Coherency | | ANOVA | | | | Correlation with Age | | | |
|---|---|---|---|---|---|---|---|---|---|---|---|
| | | Boys | Girls | Factor: Sex | | Factor: AGE | | Boys | | Girls | |
| **Lobes** | **Freq.** | **Mean ± Std** | **Mean ± Std** | **F** | **p** | **F** | **p** | **Corr. Coeff** | **p** | **Corr. Coeff** | **p** |
| L Front - L Temp | δ | 0.3 ± 0.0889 | 0.27 ± 0.083 | 4.44 | 0.037 | 0.652 | 0.78 | 21 | 0.055 | 6.6 | 0.61 |
| | θ | 0.321 ± 0.0757 | 0.29 ± 0.08 | 4.68 | 0.033 | 0.414 | 0.95 | 7.61 | 0.49 | 0.56 | 0.97 |
| | α1 | 0.325 ± 0.0772 | 0.28 ± 0.085 | 6.41 | 0.013 | 0.527 | 0.88 | 5.7 | 0.61 | -8.3 | 0.52 |
| | α2 | 0.337 ± 0.0766 | 0.29 ± 0.081 | 8.47 | 0.0043 | 0.693 | 0.74 | 5.38 | 0.63 | -15 | 0.24 |
| | β1 | 0.346 ± 0.0704 | 0.3 ± 0.078 | 9.76 | 0.0022 | 0.7 | 0.74 | 3.77 | 0.73 | -15 | 0.26 |
| | β2 | 0.339 ± 0.0733 | 0.3 ± 0.08 | 7.84 | 0.006 | 0.602 | 0.82 | 6.22 | 0.57 | -7 | 0.59 |
| | β3 | 0.331 ± 0.0787 | 0.28 ± 0.084 | 7.8 | 0.0061 | 0.816 | 0.62 | 8.7 | 0.43 | -10 | 0.44 |
| | β4 | 0.322 ± 0.0832 | 0.28 ± 0.085 | 7.39 | 0.0075 | 0.627 | 0.8 | 18.6 | 0.09 | -5.6 | 0.67 |
| | γ1 | 0.308 ± 0.0942 | 0.26 ± 0.087 | 5.67 | 0.019 | 0.618 | 0.81 | 20.9 | 0.057 | -2.7 | 0.83 |
| | γ2 | 0.293 ± 0.103 | 0.25 ± 0.094 | 4.68 | 0.033 | 0.555 | 0.86 | 18 | 0.1 | 2.1 | 0.87 |
| L Front - L Par/Occ | δ | 0.356 ± 0.0996 | 0.32 ± 0.091 | 4.46 | 0.037 | 0.789 | 0.65 | 4.8 | 0.66 | -4.4 | 0.73 |
| | α1 | 0.371 ± 0.0823 | 0.33 ± 0.098 | 6.73 | 0.011 | 1.05 | 0.41 | -8.21 | 0.46 | -17 | 0.2 |
| | α2 | 0.384 ± 0.0841 | 0.33 ± 0.096 | 8.58 | 0.0041 | 1.32 | 0.22 | -5.48 | 0.62 | -31 | 0.013 |
| | β1 | 0.394 ± 0.0738 | 0.35 ± 0.083 | 11.2 | 0.0011 | 1.83 | 0.057 | -10.5 | 0.34 | -25 | 0.05 |
| | β2 | 0.39 ± 0.0773 | 0.34 ± 0.087 | 11 | 0.0012 | 1.48 | 0.15 | -11.5 | 0.3 | -19 | 0.14 |
| | β3 | 0.386 ± 0.0823 | 0.34 ± 0.09 | 10.3 | 0.0017 | 1.72 | 0.078 | -7.47 | 0.5 | -21 | 0.1 |
| | β4 | 0.376 ± 0.089 | 0.33 ± 0.09 | 9.37 | 0.0027 | 1.49 | 0.15 | -1.24 | 0.91 | -13 | 0.3 |
| | γ1 | 0.362 ± 0.101 | 0.32 ± 0.097 | 7.29 | 0.008 | 1.18 | 0.31 | 2.91 | 0.79 | -7.2 | 0.58 |
| | γ2 | 0.345 ± 0.114 | 0.3 ± 0.1 | 6.14 | 0.015 | 0.852 | 0.59 | 5.43 | 0.62 | -2.6 | 0.84 |
| L Cent - L Temp | δ | 0.247 ± 0.0839 | 0.21 ± 0.079 | 7.14 | 0.0086 | 0.573 | 0.85 | 4.53 | 0.68 | 18 | 0.15 |
| | θ | 0.243 ± 0.0764 | 0.21 ± 0.085 | 6.69 | 0.011 | 0.606 | 0.82 | -7.85 | 0.48 | 10 | 0.43 |
| | α1 | 0.237 ± 0.0755 | 0.19 ± 0.088 | 10.6 | 0.0015 | 0.674 | 0.76 | 0.458 | 0.97 | 4 | 0.76 |
| | α2 | 0.249 ± 0.077 | 0.2 ± 0.086 | 9.73 | 0.0023 | 0.697 | 0.74 | -11.9 | 0.28 | -13 | 0.3 |
| | β1 | 0.269 ± 0.068 | 0.21 ± 0.079 | 18.4 | 3.7e-05 | 0.655 | 0.78 | -14.4 | 0.19 | -5.7 | 0.66 |
| | β2 | 0.264 ± 0.0714 | 0.21 ± 0.079 | 18.7 | 3.3e-05 | 0.707 | 0.73 | -13.8 | 0.21 | -3 | 0.81 |
| | β3 | 0.264 ± 0.0749 | 0.2 ± 0.082 | 17.2 | 6.5e-05 | 0.748 | 0.69 | -10.2 | 0.35 | -3.1 | 0.81 |
| | β4 | 0.264 ± 0.0799 | 0.2 ± 0.083 | 15.3 | 0.00016 | 0.651 | 0.78 | 0.443 | 0.97 | -0.079 | 1 |
| | γ1 | 0.26 ± 0.0892 | 0.2 ± 0.081 | 15 | 0.00017 | 0.548 | 0.87 | 3.34 | 0.76 | 2.9 | 0.82 |
| | γ2 | 0.255 ± 0.0999 | 0.19 ± 0.084 | 12.9 | 0.00048 | 0.489 | 0.91 | 2.88 | 0.79 | 8.2 | 0.53 |
| | γ3 | 0.251 ± 0.108 | 0.19 ± 0.083 | 11 | 0.0012 | 0.464 | 0.92 | 6.54 | 0.55 | 14 | 0.29 |
| | γ4 | 0.26 ± 0.105 | 0.2 ± 0.083 | 11.1 | 0.0011 | 0.52 | 0.89 | 6.15 | 0.58 | 15 | 0.24 |
| L Cent - L Par/Occ | δ | 0.267 ± 0.0881 | 0.24 ± 0.098 | 5.04 | 0.027 | 0.435 | 0.94 | 9.52 | 0.39 | -5.3 | 0.68 |
| | α1 | 0.26 ± 0.0739 | 0.22 ± 0.11 | 10.3 | 0.0017 | 1.12 | 0.35 | -0.0996 | 0.99 | -23 | 0.074 |
| | α2 | 0.268 ± 0.0756 | 0.23 ± 0.11 | 8.04 | 0.0054 | 1.06 | 0.4 | -5.03 | 0.65 | -29 | 0.024 |
| | β1 | 0.289 ± 0.0675 | 0.24 ± 0.11 | 14.9 | 0.00018 | 1.18 | 0.31 | -6.93 | 0.53 | -27 | 0.034 |
| | β2 | 0.285 ± 0.0687 | 0.23 ± 0.11 | 15.4 | 0.00014 | 1.25 | 0.26 | -9.47 | 0.39 | -26 | 0.045 |
| | β3 | 0.285 ± 0.0739 | 0.23 ± 0.11 | 14 | 0.00029 | 1.22 | 0.28 | -9.64 | 0.38 | -23 | 0.068 |
| | β4 | 0.283 ± 0.0777 | 0.22 ± 0.1 | 14.8 | 0.00019 | 1.06 | 0.4 | -0.78 | 0.94 | -21 | 0.1 |
| | γ1 | 0.278 ± 0.0896 | 0.22 ± 0.1 | 13.6 | 0.00034 | 0.75 | 0.69 | 3.35 | 0.76 | -13 | 0.31 |
| | γ2 | 0.268 ± 0.099 | 0.21 ± 0.1 | 11.2 | 0.0011 | 0.664 | 0.77 | 4.35 | 0.69 | -8.7 | 0.5 |
| | γ3 | 0.262 ± 0.108 | 0.2 ± 0.098 | 10 | 0.002 | 0.694 | 0.74 | 5.69 | 0.61 | -2.9 | 0.82 |
| | γ4 | 0.271 ± 0.108 | 0.21 ± 0.097 | 9.8 | 0.0022 | 0.757 | 0.68 | 7.26 | 0.51 | -3.3 | 0.8 |
| L Temp - L Par/Occ | γ1 | 0.237 ± 0.0886 | 0.2 ± 0.083 | 4.75 | 0.031 | 0.481 | 0.91 | 18.4 | 0.094 | 9.3 | 0.47 |
| R Front - R Temp | δ | 0.306 ± 0.0833 | 0.27 ± 0.075 | 4.71 | 0.032 | 0.725 | 0.71 | 16.4 | 0.13 | 3.9 | 0.77 |
| | θ | 0.334 ± 0.0731 | 0.29 ± 0.074 | 7.82 | 0.006 | 1.05 | 0.41 | 6.98 | 0.53 | -0.74 | 0.95 |
| | α1 | 0.339 ± 0.0751 | 0.29 ± 0.082 | 8.88 | 0.0035 | 1.02 | 0.44 | 6.72 | 0.54 | -9 | 0.49 |
| | α2 | 0.35 ± 0.0768 | 0.3 ± 0.076 | 8.39 | 0.0045 | 1.19 | 0.3 | 0.958 | 0.93 | -17 | 0.2 |
| | β1 | 0.357 ± 0.0636 | 0.3 ± 0.072 | 13.2 | 0.00041 | 1.37 | 0.2 | 3.27 | 0.77 | -18 | 0.15 |
| | β2 | 0.352 ± 0.0655 | 0.3 ± 0.075 | 12.4 | 0.0006 | 1.21 | 0.29 | 7.81 | 0.48 | -9.8 | 0.45 |
| | β3 | 0.346 ± 0.0686 | 0.3 ± 0.078 | 10.9 | 0.0012 | 1.21 | 0.29 | 9.51 | 0.39 | -11 | 0.38 |
| | β4 | 0.332 ± 0.0722 | 0.28 ± 0.08 | 8.94 | 0.0034 | 1.11 | 0.36 | 12.5 | 0.26 | -10 | 0.42 |
| | γ1 | 0.315 ± 0.0836 | 0.27 ± 0.085 | 6.33 | 0.013 | 0.865 | 0.58 | 16.3 | 0.14 | -7.8 | 0.55 |
| | γ2 | 0.299 ± 0.0936 | 0.25 ± 0.09 | 6.39 | 0.013 | 0.758 | 0.68 | 17.1 | 0.12 | -4.7 | 0.72 |
| | γ3 | 0.284 ± 0.0964 | 0.24 ± 0.094 | 4.65 | 0.033 | 0.562 | 0.86 | 15.2 | 0.17 | -1.9 | 0.89 |
| | γ4 | 0.292 ± 0.0961 | 0.25 ± 0.091 | 4.86 | 0.029 | 0.713 | 0.72 | 17.1 | 0.12 | -1.6 | 0.9 |
| R Front - R Par/Occ | β1 | 0.407 ± 0.0705 | 0.37 ± 0.078 | 4.89 | 0.029 | 1.16 | 0.32 | -12.1 | 0.27 | -12 | 0.35 |

| | | | | | | | | | | | |
|---|---|---|---|---|---|---|---|---|---|---|---|
| | β2 | 0.401 ± 0.0735 | 0.37 ± 0.081 | 5.65 | 0.019 | 1.01 | 0.44 | -8.25 | 0.46 | -2.6 | 0.84 |
| | β3 | 0.396 ± 0.077 | 0.36 ± 0.082 | 5.27 | 0.023 | 1.25 | 0.26 | -7.97 | 0.47 | -6.7 | 0.6 |
| | β4 | 0.388 ± 0.0837 | 0.35 ± 0.084 | 5.12 | 0.025 | 1.09 | 0.38 | -3.2 | 0.77 | -7.4 | 0.57 |
| R Cent - R Temp | α1 | 0.235 ± 0.0712 | 0.2 ± 0.089 | 5.25 | 0.024 | 0.769 | 0.67 | 0.511 | 0.96 | -9.1 | 0.48 |
| | α2 | 0.25 ± 0.0704 | 0.21 ± 0.086 | 6.01 | 0.016 | 0.818 | 0.62 | -11.2 | 0.31 | -21 | 0.1 |
| | β1 | 0.27 ± 0.0642 | 0.22 ± 0.084 | 8.83 | 0.0036 | 0.731 | 0.71 | -3.61 | 0.74 | -14 | 0.28 |
| | β2 | 0.268 ± 0.0665 | 0.22 ± 0.086 | 8.43 | 0.0044 | 0.598 | 0.83 | -2.58 | 0.82 | -7.3 | 0.57 |
| | β3 | 0.267 ± 0.0705 | 0.23 ± 0.088 | 6.65 | 0.011 | 0.699 | 0.74 | 1.12 | 0.92 | -8.2 | 0.52 |
| | β4 | 0.264 ± 0.0738 | 0.22 ± 0.09 | 6.53 | 0.012 | 0.611 | 0.82 | 7.85 | 0.48 | -3.7 | 0.78 |
| | γ1 | 0.258 ± 0.0826 | 0.22 ± 0.094 | 5.72 | 0.018 | 0.512 | 0.89 | 10.9 | 0.32 | -1.3 | 0.92 |
| | γ2 | 0.248 ± 0.0906 | 0.2 ± 0.097 | 6.09 | 0.015 | 0.516 | 0.89 | 11.1 | 0.31 | 2.5 | 0.85 |
| | γ3 | 0.241 ± 0.0966 | 0.2 ± 0.097 | 4.83 | 0.03 | 0.394 | 0.96 | 12.4 | 0.26 | 5.8 | 0.65 |
| | γ4 | 0.25 ± 0.0961 | 0.21 ± 0.097 | 4.31 | 0.04 | 0.491 | 0.91 | 10.8 | 0.33 | 7.9 | 0.54 |
| L Front - R Front | β3 | 0.367 ± 0.0834 | 0.36 ± 0.083 | 0.0008 | 0.98 | 2.08 | 0.027 | -5.52 | 0.62 | -21 | 0.1 |
| L Cent - R Cent | α1 | 0.367 ± 0.103 | 0.33 ± 0.12 | 2.63 | 0.11 | 2.17 | 0.021 | -12 | 0.28 | -35 | 0.0053 |
| | α2 | 0.375 ± 0.0979 | 0.34 ± 0.12 | 2.95 | 0.089 | 2.61 | 0.0052 | -9.55 | 0.39 | -39 | 0.0018 |
| | β1 | 0.386 ± 0.0896 | 0.35 ± 0.11 | 4.19 | 0.043 | 2.56 | 0.006 | -13.5 | 0.22 | -34 | 0.0073 |
| | β2 | 0.383 ± 0.0932 | 0.35 ± 0.11 | 3.52 | 0.063 | 2.53 | 0.0067 | -12.4 | 0.26 | -29 | 0.023 |
| | β3 | 0.374 ± 0.104 | 0.34 ± 0.11 | 3.57 | 0.061 | 2.75 | 0.0033 | -13.5 | 0.22 | -28 | 0.026 |
| | β4 | 0.366 ± 0.109 | 0.33 ± 0.12 | 3.45 | 0.066 | 2.43 | 0.0091 | -10.4 | 0.34 | -24 | 0.056 |
| | γ1 | 0.344 ± 0.118 | 0.31 ± 0.12 | 3.14 | 0.079 | 2.17 | 0.021 | -6.15 | 0.58 | -17 | 0.18 |
| L Par/Occ - R Par/Occ | β4 | 0.252 ± 0.0875 | 0.21 ± 0.11 | 4.8 | 0.03 | 0.7 | 0.74 | -2.28 | 0.84 | 4.6 | 0.72 |
| | γ1 | 0.246 ± 0.096 | 0.21 ± 0.1 | 4.68 | 0.033 | 0.667 | 0.77 | 2.69 | 0.81 | 8.7 | 0.5 |
| | γ2 | 0.234 ± 0.1 | 0.19 ± 0.1 | 4.71 | 0.032 | 0.723 | 0.72 | 4.24 | 0.7 | 14 | 0.28 |
| L Front - R Cent | β3 | 0.388 ± 0.086 | 0.38 ± 0.085 | 0.637 | 0.43 | 2.21 | 0.018 | 0.432 | 0.97 | -27 | 0.033 |
| L Front - R Temp | α2 | 0.396 ± 0.0734 | 0.37 ± 0.08 | 1.93 | 0.17 | 2.06 | 0.029 | -0.796 | 0.94 | -37 | 0.0034 |
| | β1 | 0.404 ± 0.0679 | 0.38 ± 0.071 | 2.21 | 0.14 | 2.14 | 0.022 | -5.5 | 0.62 | -33 | 0.0085 |
| | β3 | 0.39 ± 0.0751 | 0.37 ± 0.082 | 1.26 | 0.26 | 2.37 | 0.011 | 0.359 | 0.97 | -29 | 0.021 |
| L Front - R Par/Occ | α1 | 0.396 ± 0.0819 | 0.36 ± 0.09 | 4.63 | 0.033 | 0.965 | 0.48 | -9.92 | 0.37 | -17 | 0.18 |
| | α2 | 0.407 ± 0.0819 | 0.37 ± 0.08 | 5.99 | 0.016 | 1.35 | 0.21 | -8.02 | 0.47 | -21 | 0.11 |
| | β1 | 0.419 ± 0.0706 | 0.39 ± 0.076 | 6.71 | 0.011 | 1.56 | 0.12 | -7.57 | 0.49 | -18 | 0.15 |
| | β2 | 0.417 ± 0.073 | 0.38 ± 0.08 | 6.78 | 0.01 | 1.45 | 0.16 | -9.2 | 0.41 | -12 | 0.35 |
| | β3 | 0.41 ± 0.0783 | 0.38 ± 0.084 | 5.49 | 0.021 | 1.71 | 0.078 | -4.81 | 0.66 | -17 | 0.19 |
| | β4 | 0.401 ± 0.0864 | 0.37 ± 0.09 | 5.3 | 0.023 | 1.61 | 0.1 | 0.399 | 0.97 | -15 | 0.23 |
| L Cent - R Temp | α2 | 0.382 ± 0.0878 | 0.34 ± 0.1 | 4.53 | 0.035 | 2.19 | 0.019 | -10.8 | 0.33 | -40 | 0.0014 |
| | β1 | 0.394 ± 0.0803 | 0.35 ± 0.092 | 7.62 | 0.0067 | 2.15 | 0.022 | -11.7 | 0.29 | -36 | 0.0041 |
| | β2 | 0.391 ± 0.0835 | 0.35 ± 0.091 | 6.1 | 0.015 | 2.16 | 0.021 | -12.3 | 0.27 | -32 | 0.01 |
| | β3 | 0.38 ± 0.0908 | 0.34 ± 0.097 | 5.28 | 0.023 | 2.58 | 0.0057 | -11.8 | 0.29 | -30 | 0.019 |
| | β4 | 0.369 ± 0.0948 | 0.33 ± 0.1 | 4.5 | 0.036 | 2.1 | 0.025 | -5.49 | 0.62 | -27 | 0.033 |
| | γ1 | 0.352 ± 0.105 | 0.31 ± 0.1 | 4.89 | 0.029 | 1.76 | 0.07 | -2.83 | 0.8 | -18 | 0.16 |
| | γ2 | 0.331 ± 0.118 | 0.28 ± 0.1 | 4.74 | 0.031 | 1.53 | 0.13 | -1.17 | 0.92 | -9.7 | 0.45 |
| L Cent - R Par/Occ | δ | 0.331 ± 0.115 | 0.3 ± 0.097 | 4.86 | 0.029 | 0.808 | 0.63 | 5.2 | 0.64 | -5.8 | 0.66 |
| | θ | 0.36 ± 0.104 | 0.32 ± 0.1 | 5.06 | 0.026 | 1.15 | 0.33 | -9.14 | 0.41 | -21 | 0.1 |
| | α1 | 0.373 ± 0.103 | 0.32 ± 0.12 | 8.66 | 0.0039 | 1.55 | 0.12 | -9.21 | 0.4 | -22 | 0.083 |
| | α2 | 0.38 ± 0.102 | 0.32 ± 0.11 | 9.2 | 0.003 | 1.23 | 0.27 | -9.77 | 0.38 | -29 | 0.023 |
| | β1 | 0.39 ± 0.0893 | 0.33 ± 0.11 | 13.3 | 0.00039 | 1.57 | 0.11 | -11.8 | 0.28 | -24 | 0.066 |
| | β2 | 0.388 ± 0.0935 | 0.33 ± 0.11 | 11.5 | 0.00096 | 1.39 | 0.19 | -13.5 | 0.22 | -23 | 0.068 |
| | β3 | 0.38 ± 0.0989 | 0.32 ± 0.11 | 11.9 | 0.00079 | 1.5 | 0.14 | -10.8 | 0.33 | -22 | 0.088 |
| | β4 | 0.37 ± 0.102 | 0.31 ± 0.11 | 11.4 | 0.001 | 1.25 | 0.26 | -6.76 | 0.54 | -22 | 0.092 |
| | γ1 | 0.353 ± 0.113 | 0.29 ± 0.1 | 9.95 | 0.002 | 1.1 | 0.37 | -0.18 | 0.99 | -16 | 0.23 |
| | γ2 | 0.333 ± 0.122 | 0.27 ± 0.11 | 8.68 | 0.0039 | 0.896 | 0.55 | 1.16 | 0.92 | -9.3 | 0.47 |
| | γ3 | 0.323 ± 0.131 | 0.27 ± 0.11 | 6.26 | 0.014 | 0.917 | 0.53 | -0.307 | 0.98 | -1.9 | 0.88 |
| | γ4 | 0.327 ± 0.13 | 0.28 ± 0.11 | 5.62 | 0.019 | 0.991 | 0.46 | 2.1 | 0.85 | -2 | 0.88 |
| L Temp - R Par/Occ | β1 | 0.336 ± 0.0823 | 0.3 ± 0.092 | 5.39 | 0.022 | 1.1 | 0.37 | -1.13 | 0.92 | -10 | 0.42 |
| | β2 | 0.338 ± 0.0882 | 0.3 ± 0.097 | 5.51 | 0.021 | 0.944 | 0.5 | -0.492 | 0.96 | -8.2 | 0.53 |
| | β3 | 0.329 ± 0.0909 | 0.29 ± 0.094 | 5.83 | 0.017 | 0.984 | 0.46 | -0.194 | 0.99 | -7.7 | 0.55 |
| | β4 | 0.33 ± 0.0922 | 0.28 ± 0.095 | 7.99 | 0.0055 | 0.855 | 0.59 | 5.82 | 0.6 | -6.8 | 0.6 |
| | γ1 | 0.319 ± 0.104 | 0.27 ± 0.094 | 7.34 | 0.0078 | 0.81 | 0.63 | 12.2 | 0.27 | -0.68 | 0.96 |
| | γ2 | 0.301 ± 0.111 | 0.25 ± 0.097 | 6.83 | 0.01 | 0.645 | 0.79 | 11.6 | 0.29 | 4 | 0.76 |
| | γ3 | 0.294 ± 0.122 | 0.24 ± 0.1 | 4.96 | 0.028 | 0.633 | 0.8 | 13.1 | 0.23 | 8.9 | 0.49 |
| R Cent - L Temp | β1 | 0.392 ± 0.08 | 0.36 ± 0.095 | 5.85 | 0.017 | 1.43 | 0.17 | 1.44 | 0.9 | -16 | 0.23 |
| | β2 | 0.391 ± 0.0841 | 0.35 ± 0.1 | 5.5 | 0.021 | 1.52 | 0.13 | 3.21 | 0.77 | -10 | 0.44 |
| | β3 | 0.379 ± 0.0912 | 0.35 ± 0.1 | 3.94 | 0.049 | 1.96 | 0.038 | 1.94 | 0.86 | -13 | 0.32 |
| | β4 | 0.369 ± 0.0949 | 0.34 ± 0.1 | 4.85 | 0.03 | 1.79 | 0.063 | 2.86 | 0.8 | -9.5 | 0.46 |

| | | | | | | | | | | | |
|---|---|---|---|---|---|---|---|---|---|---|---|
| R Cent - L Par/Occ | β1 | 0.39 ± 0.0848 | 0.34 ± 0.12 | 6.99 | 0.0093 | 1.6 | 0.11 | -6.13 | 0.58 | -16 | 0.21 |
| | β2 | 0.389 ± 0.0891 | 0.34 ± 0.12 | 6.78 | 0.01 | 1.22 | 0.28 | -2.77 | 0.8 | -13 | 0.31 |
| | β3 | 0.381 ± 0.0973 | 0.34 ± 0.13 | 4.65 | 0.033 | 1.46 | 0.15 | -2.48 | 0.82 | -15 | 0.24 |
| | β4 | 0.375 ± 0.103 | 0.33 ± 0.13 | 5.33 | 0.023 | 1.26 | 0.26 | -0.0193 | 1 | -13 | 0.33 |
| | γ1 | 0.357 ± 0.112 | 0.31 ± 0.13 | 4.62 | 0.034 | 1.18 | 0.31 | 4.19 | 0.71 | -8.4 | 0.51 |
| R Temp - L Par/Occ | β2 | 0.346 ± 0.089 | 0.31 ± 0.098 | 4.36 | 0.039 | 1.19 | 0.3 | -10.8 | 0.33 | -14 | 0.29 |
| | β4 | 0.338 ± 0.0954 | 0.3 ± 0.1 | 4.61 | 0.034 | 1.17 | 0.32 | -5.1 | 0.65 | -10 | 0.42 |
| | γ1 | 0.326 ± 0.106 | 0.28 ± 0.1 | 4.74 | 0.031 | 0.974 | 0.47 | -0.92 | 0.93 | -4.7 | 0.72 |
| | γ2 | 0.306 ± 0.114 | 0.26 ± 0.1 | 4.89 | 0.029 | 0.896 | 0.55 | 0.788 | 0.94 | 2 | 0.88_ |

**Table S9.** Comparison of boys and girls magnitude of **Phase Synchrony** during **Eyes Open** condition.

Significant Sex Factor    Significant Age Factor

| Eyes Open | | Phase Coherency | | ANOVA | | | | Correlation with Age | | | |
|---|---|---|---|---|---|---|---|---|---|---|---|
| | | Male | Female | SEX | | AGE | | Boys | | Girls | |
| Lobes | Freq. | Mean ± Std | Mean ± Std | F | p | F | p | Corr. Coeff | p | Corr. Coeff | p |
| L Front - L Temp | δ | 0.302 ± 0.0848 | 0.27 ± 0.088 | 4.89 | 0.029 | 0.502 | 0.9 | 17 | 0.12 | -3.4 | 0.79 |
| | θ | 0.324 ± 0.072 | 0.29 ± 0.082 | 4.63 | 0.033 | 0.395 | 0.96 | 3.25 | 0.77 | -1.2 | 0.93 |
| | α1 | 0.331 ± 0.0718 | 0.29 ± 0.085 | 5.73 | 0.018 | 0.462 | 0.92 | 1.81 | 0.87 | -11 | 0.38 |
| | α2 | 0.338 ± 0.0711 | 0.3 ± 0.085 | 7.08 | 0.0089 | 0.609 | 0.82 | 9.85 | 0.37 | -16 | 0.21 |
| | β1 | 0.345 ± 0.07 | 0.3 ± 0.081 | 8.82 | 0.0036 | 0.811 | 0.63 | 4.32 | 0.7 | -16 | 0.2 |
| | β2 | 0.341 ± 0.0745 | 0.3 ± 0.086 | 7.56 | 0.0069 | 0.722 | 0.72 | 6.34 | 0.57 | -9.1 | 0.48 |
| | β3 | 0.329 ± 0.0781 | 0.29 ± 0.086 | 5.68 | 0.019 | 0.684 | 0.75 | 6.53 | 0.56 | -12 | 0.36 |
| | β4 | 0.323 ± 0.0831 | 0.28 ± 0.087 | 5.29 | 0.023 | 0.598 | 0.83 | 16.6 | 0.13 | -12 | 0.37 |
| | γ1 | 0.317 ± 0.0892 | 0.28 ± 0.089 | 4.36 | 0.039 | 0.596 | 0.83 | 20.7 | 0.059 | -8.4 | 0.52 |
| L Front - L Par/Occ | δ | 0.355 ± 0.097 | 0.32 ± 0.091 | 6.11 | 0.015 | 0.653 | 0.78 | 2.33 | 0.83 | -8.7 | 0.5 |
| | θ | 0.375 ± 0.0844 | 0.35 ± 0.087 | 4.4 | 0.038 | 1.12 | 0.35 | -8.74 | 0.43 | -21 | 0.11 |
| | α1 | 0.386 ± 0.0796 | 0.35 ± 0.098 | 6.48 | 0.012 | 1.21 | 0.29 | -5.53 | 0.62 | -21 | 0.096 |
| | α2 | 0.39 ± 0.0781 | 0.35 ± 0.091 | 8.77 | 0.0037 | 1.42 | 0.17 | -1.3 | 0.91 | -29 | 0.025 |
| | β1 | 0.396 ± 0.0756 | 0.35 ± 0.086 | 11.5 | 0.00096 | 1.65 | 0.094 | -9.81 | 0.37 | -25 | 0.051 |
| | β2 | 0.393 ± 0.0784 | 0.35 ± 0.09 | 10.7 | 0.0014 | 1.61 | 0.1 | -10 | 0.37 | -19 | 0.14 |
| | β3 | 0.382 ± 0.0832 | 0.34 ± 0.093 | 8.16 | 0.0051 | 1.68 | 0.087 | -9.65 | 0.38 | -23 | 0.077 |
| | β4 | 0.378 ± 0.089 | 0.33 ± 0.096 | 8.23 | 0.0049 | 1.37 | 0.19 | -1.87 | 0.87 | -18 | 0.15 |
| | γ1 | 0.369 ± 0.0943 | 0.33 ± 0.097 | 5.82 | 0.017 | 1.3 | 0.23 | 3.1 | 0.78 | -16 | 0.21 |
| | γ2 | 0.358 ± 0.102 | 0.32 ± 0.1 | 4.99 | 0.027 | 0.937 | 0.51 | 4.66 | 0.67 | -8.2 | 0.52 |
| L Cent - L Temp | δ | 0.25 ± 0.0889 | 0.21 ± 0.081 | 6.15 | 0.015 | 0.52 | 0.89 | 3.44 | 0.76 | 13 | 0.33 |
| | θ | 0.252 ± 0.0766 | 0.21 ± 0.083 | 6.95 | 0.0095 | 0.617 | 0.81 | -9.55 | 0.39 | 6.9 | 0.59 |
| | α1 | 0.251 ± 0.0725 | 0.2 ± 0.084 | 10.5 | 0.0015 | 0.684 | 0.75 | -7.81 | 0.48 | -2.1 | 0.87 |
| | α2 | 0.263 ± 0.0723 | 0.21 ± 0.085 | 16.7 | 8e-05 | 0.629 | 0.8 | -15 | 0.17 | -7 | 0.59 |
| | β1 | 0.273 ± 0.0683 | 0.22 ± 0.08 | 18.3 | 3.9e-05 | 0.488 | 0.91 | -15 | 0.17 | -7.4 | 0.57 |
| | β2 | 0.269 ± 0.0727 | 0.21 ± 0.081 | 17.8 | 4.8e-05 | 0.826 | 0.61 | -13.5 | 0.22 | -5.9 | 0.65 |
| | β3 | 0.263 ± 0.0752 | 0.21 ± 0.08 | 14.5 | 0.00022 | 0.647 | 0.79 | -12 | 0.28 | -5.8 | 0.65 |
| | β4 | 0.266 ± 0.0811 | 0.21 ± 0.081 | 14.1 | 0.00027 | 0.579 | 0.84 | -4.04 | 0.71 | -8 | 0.54 |
| | γ1 | 0.266 ± 0.0888 | 0.21 ± 0.078 | 13.3 | 0.00039 | 0.601 | 0.82 | 2.89 | 0.79 | -4.1 | 0.75 |
| | γ2 | 0.267 ± 0.0939 | 0.2 ± 0.08 | 14.4 | 0.00024 | 0.462 | 0.92 | 2.29 | 0.84 | 2.3 | 0.86 |
| | γ3 | 0.264 ± 0.102 | 0.2 ± 0.083 | 11.9 | 0.00076 | 0.366 | 0.97 | 1.08 | 0.92 | 8 | 0.54 |
| | γ4 | 0.272 ± 0.102 | 0.21 ± 0.081 | 11.8 | 0.00082 | 0.276 | 0.99 | 5.78 | 0.6 | 11 | 0.41 |
| L Cent - L Par/Occ | α1 | 0.277 ± 0.0754 | 0.23 ± 0.11 | 11.4 | 0.001 | 0.851 | 0.59 | -5.12 | 0.64 | -23 | 0.076 |
| | α2 | 0.286 ± 0.0741 | 0.24 ± 0.11 | 13.2 | 0.00041 | 1.16 | 0.32 | -9.28 | 0.4 | -31 | 0.013 |
| | β1 | 0.298 ± 0.0695 | 0.24 ± 0.1 | 17.3 | 6e-05 | 1.14 | 0.34 | -8.27 | 0.45 | -30 | 0.017 |
| | β2 | 0.292 ± 0.0732 | 0.24 ± 0.11 | 13.1 | 0.00043 | 1.2 | 0.3 | -9.23 | 0.4 | -27 | 0.035 |
| | β3 | 0.287 ± 0.0775 | 0.24 ± 0.1 | 12.8 | 0.00051 | 1.24 | 0.27 | -9.15 | 0.41 | -27 | 0.034 |
| | β4 | 0.288 ± 0.0813 | 0.23 ± 0.11 | 13.8 | 0.0003 | 0.927 | 0.52 | -2.83 | 0.8 | -26 | 0.041 |
| | γ1 | 0.286 ± 0.0893 | 0.23 ± 0.1 | 12.4 | 0.00061 | 0.832 | 0.61 | 2.99 | 0.79 | -22 | 0.093 |
| | γ2 | 0.281 ± 0.093 | 0.22 ± 0.099 | 12.4 | 0.00061 | 0.663 | 0.77 | 1.59 | 0.89 | -15 | 0.25 |
| | γ3 | 0.276 ± 0.104 | 0.22 ± 0.1 | 8.87 | 0.0035 | 0.736 | 0.7 | -1.23 | 0.91 | -12 | 0.36 |
| | γ4 | 0.282 ± 0.104 | 0.22 ± 0.1 | 10.1 | 0.0019 | 0.633 | 0.8 | 2.58 | 0.82 | -6.9 | 0.59 |
| L Temp - L Par/Occ | β4 | 0.239 ± 0.0785 | 0.2 ± 0.081 | 4.43 | 0.037 | 0.495 | 0.9 | 9.08 | 0.41 | 0.63 | 0.96 |
| | γ1 | 0.244 ± 0.0858 | 0.21 ± 0.082 | 4.68 | 0.033 | 0.367 | 0.97 | 13.8 | 0.21 | 4.3 | 0.74 |
| | γ2 | 0.244 ± 0.0888 | 0.2 ± 0.085 | 4.92 | 0.028 | 0.281 | 0.99 | 16 | 0.15 | 10 | 0.43 |
| R Front - R Temp | θ | 0.336 ± 0.0738 | 0.3 ± 0.074 | 6.68 | 0.011 | 0.747 | 0.69 | 7.77 | 0.48 | -12 | 0.35 |
| | α1 | 0.341 ± 0.0697 | 0.29 ± 0.075 | 10.5 | 0.0016 | 1.29 | 0.24 | 0.056 | 1 | -18 | 0.17 |
| | α2 | 0.347 ± 0.0649 | 0.31 ± 0.074 | 7.31 | 0.0078 | 0.993 | 0.46 | 4.04 | 0.72 | -27 | 0.035 |
| | β1 | 0.357 ± 0.0637 | 0.31 ± 0.075 | 11.8 | 0.00083 | 1.56 | 0.12 | 8.68 | 0.43 | -25 | 0.049 |
| | β2 | 0.355 ± 0.066 | 0.31 ± 0.077 | 11.4 | 0.001 | 1.28 | 0.25 | 9.45 | 0.39 | -16 | 0.21 |
| | β3 | 0.346 ± 0.0701 | 0.3 ± 0.078 | 10.2 | 0.0018 | 0.947 | 0.5 | 9.48 | 0.39 | -15 | 0.24 |
| | β4 | 0.335 ± 0.0739 | 0.29 ± 0.077 | 6.98 | 0.0093 | 0.879 | 0.56 | 10 | 0.36 | -17 | 0.19 |
| | γ1 | 0.325 ± 0.0812 | 0.28 ± 0.083 | 6.36 | 0.013 | 0.758 | 0.68 | 14.7 | 0.18 | -15 | 0.25 |
| | γ2 | 0.315 ± 0.0866 | 0.27 ± 0.086 | 5.52 | 0.02 | 0.625 | 0.8 | 13.8 | 0.21 | -8.2 | 0.53 |
| | γ3 | 0.306 ± 0.0942 | 0.26 ± 0.091 | 4.73 | 0.032 | 0.47 | 0.92 | 10.2 | 0.36 | -5.7 | 0.66 |
| R Front - R Par/Occ | β1 | 0.408 ± 0.071 | 0.37 ± 0.078 | 4.56 | 0.035 | 1.31 | 0.23 | -8.81 | 0.43 | -18 | 0.16 |

| | | | | | | | | | | | |
|---|---|---|---|---|---|---|---|---|---|---|---|
| | β2 | 0.404 ± 0.0761 | 0.37 ± 0.082 | 4.69 | 0.032 | 1.1 | 0.37 | -3.9 | 0.72 | -5.8 | 0.66 |
| R Cent - R Temp | α1 | 0.251 ± 0.0761 | 0.21 ± 0.089 | 5.42 | 0.022 | 0.526 | 0.88 | -4.93 | 0.66 | -9.5 | 0.46 |
| | α2 | 0.259 ± 0.0682 | 0.22 ± 0.09 | 5.95 | 0.016 | 0.405 | 0.95 | -4.78 | 0.67 | -16 | 0.22 |
| | β1 | 0.276 ± 0.064 | 0.23 ± 0.082 | 11.2 | 0.0011 | 0.625 | 0.8 | -2.62 | 0.81 | -13 | 0.31 |
| | β2 | 0.272 ± 0.0655 | 0.23 ± 0.085 | 8.57 | 0.0041 | 0.67 | 0.76 | 2.06 | 0.85 | -11 | 0.39 |
| | β3 | 0.268 ± 0.0709 | 0.23 ± 0.087 | 7.68 | 0.0065 | 0.483 | 0.91 | 1.38 | 0.9 | -7.5 | 0.56 |
| | β4 | 0.269 ± 0.075 | 0.23 ± 0.085 | 7.77 | 0.0062 | 0.528 | 0.88 | 7.73 | 0.48 | -4.5 | 0.73 |
| | γ1 | 0.265 ± 0.0809 | 0.22 ± 0.087 | 6.74 | 0.011 | 0.549 | 0.87 | 9.65 | 0.38 | -5 | 0.7 |
| | γ2 | 0.262 ± 0.0852 | 0.22 ± 0.089 | 6.79 | 0.01 | 0.456 | 0.93 | 7.56 | 0.49 | -1 | 0.94 |
| | γ3 | 0.257 ± 0.0942 | 0.21 ± 0.094 | 5.35 | 0.022 | 0.407 | 0.95 | 7.13 | 0.52 | 2.7 | 0.83 |
| | γ4 | 0.264 ± 0.0937 | 0.22 ± 0.095 | 4.7 | 0.032 | 0.44 | 0.93 | 6.44 | 0.56 | 4.8 | 0.71 |
| R Cent - R Par/Occ | β1 | 0.308 ± 0.0661 | 0.27 ± 0.1 | 4.51 | 0.036 | 0.66 | 0.77 | 4.74 | 0.67 | -11 | 0.41 |
| L Cent - R Cent | α2 | 0.385 ± 0.0926 | 0.35 ± 0.11 | 4.3 | 0.04 | 2.16 | 0.021 | -11.5 | 0.3 | -40 | 0.0013 |
| | β1 | 0.387 ± 0.0935 | 0.35 ± 0.11 | 5.56 | 0.02 | 2.42 | 0.0093 | -11.3 | 0.31 | -33 | 0.0099 |
| | β2 | 0.382 ± 0.0984 | 0.35 ± 0.11 | 2.94 | 0.089 | 2.49 | 0.0076 | -10.3 | 0.35 | -32 | 0.012 |
| | β3 | 0.372 ± 0.107 | 0.34 ± 0.11 | 3.18 | 0.077 | 2.38 | 0.011 | -13.9 | 0.21 | -32 | 0.012 |
| | β4 | 0.367 ± 0.111 | 0.34 ± 0.11 | 3.1 | 0.081 | 2.07 | 0.028 | -9.45 | 0.39 | -26 | 0.041 |
| L Par/Occ - R Par/Occ | β4 | 0.25 ± 0.086 | 0.21 ± 0.1 | 5.37 | 0.022 | 1.08 | 0.38 | -8.64 | 0.43 | 4.3 | 0.74 |
| | γ1 | 0.252 ± 0.0923 | 0.22 ± 0.1 | 4.99 | 0.027 | 0.75 | 0.69 | -2.16 | 0.85 | 8.3 | 0.52 |
| L Front - R Par/Occ | δ | 0.37 ± 0.0972 | 0.34 ± 0.089 | 4.44 | 0.037 | 0.922 | 0.52 | 7.66 | 0.49 | -8.2 | 0.53 |
| | α2 | 0.414 ± 0.0754 | 0.39 ± 0.075 | 4.41 | 0.038 | 1.13 | 0.34 | -1.84 | 0.87 | -31 | 0.014 |
| | β1 | 0.421 ± 0.0701 | 0.39 ± 0.076 | 7.59 | 0.0068 | 1.5 | 0.14 | -1.66 | 0.88 | -16 | 0.23 |
| | β2 | 0.418 ± 0.0736 | 0.39 ± 0.078 | 5.86 | 0.017 | 1.29 | 0.24 | -2.89 | 0.79 | -12 | 0.34 |
| | β3 | 0.407 ± 0.0794 | 0.38 ± 0.085 | 4.5 | 0.036 | 1.43 | 0.17 | -3.14 | 0.78 | -20 | 0.11 |
| | β4 | 0.402 ± 0.0853 | 0.37 ± 0.092 | 4.68 | 0.033 | 1.36 | 0.2 | 1.81 | 0.87 | -18 | 0.15 |
| L Cent - R Temp | α1 | 0.38 ± 0.0874 | 0.34 ± 0.1 | 5.03 | 0.027 | 1.85 | 0.053 | -12 | 0.28 | -40 | 0.0013 |
| | α2 | 0.394 ± 0.0835 | 0.35 ± 0.098 | 5.65 | 0.019 | 2.01 | 0.034 | -14.9 | 0.17 | -44 | 0.00039 |
| | β1 | 0.392 ± 0.0824 | 0.35 ± 0.095 | 5.89 | 0.017 | 2.37 | 0.011 | -10.1 | 0.36 | -35 | 0.0051 |
| | β2 | 0.387 ± 0.0863 | 0.36 ± 0.095 | 2.78 | 0.098 | 2.09 | 0.026 | -14.5 | 0.19 | -34 | 0.007 |
| | β3 | 0.376 ± 0.092 | 0.34 ± 0.099 | 4.29 | 0.041 | 2.04 | 0.03 | -9.94 | 0.37 | -35 | 0.0053 |
| L Cent - R Par/Occ | θ | 0.354 ± 0.0995 | 0.32 ± 0.099 | 4.48 | 0.036 | 0.882 | 0.56 | -5.95 | 0.59 | -26 | 0.04 |
| | α1 | 0.379 ± 0.0949 | 0.33 ± 0.11 | 9.4 | 0.0027 | 1.1 | 0.37 | -10.9 | 0.32 | -25 | 0.049 |
| | α2 | 0.389 ± 0.0949 | 0.34 ± 0.11 | 10.6 | 0.0015 | 1.23 | 0.27 | -8.85 | 0.42 | -33 | 0.009 |
| | β1 | 0.392 ± 0.0895 | 0.34 ± 0.11 | 13.2 | 0.00041 | 1.48 | 0.15 | -7.66 | 0.49 | -23 | 0.071 |
| | β2 | 0.383 ± 0.0939 | 0.33 ± 0.11 | 9.09 | 0.0031 | 1.24 | 0.27 | -12.3 | 0.26 | -24 | 0.056 |
| | β3 | 0.374 ± 0.0999 | 0.32 ± 0.11 | 9.16 | 0.003 | 1.39 | 0.19 | -10.5 | 0.34 | -28 | 0.03 |
| | β4 | 0.371 ± 0.105 | 0.31 ± 0.11 | 9.89 | 0.0021 | 1.23 | 0.27 | -6.87 | 0.53 | -27 | 0.033 |
| | γ1 | 0.361 ± 0.111 | 0.31 ± 0.1 | 7.91 | 0.0058 | 1.02 | 0.43 | -3.37 | 0.76 | -18 | 0.15 |
| | γ2 | 0.351 ± 0.116 | 0.29 ± 0.1 | 9.51 | 0.0025 | 0.912 | 0.53 | -2.6 | 0.81 | -11 | 0.4 |
| | γ3 | 0.339 ± 0.125 | 0.29 ± 0.1 | 6.67 | 0.011 | 0.798 | 0.64 | -4.54 | 0.68 | -5 | 0.7 |
| | γ4 | 0.339 ± 0.124 | 0.29 ± 0.1 | 5.48 | 0.021 | 0.723 | 0.71 | -1.68 | 0.88 | -1.7 | 0.89 |
| L Temp - R Par/Occ | β1 | 0.343 ± 0.0844 | 0.3 ± 0.095 | 5.71 | 0.018 | 0.993 | 0.46 | 4.66 | 0.67 | -13 | 0.3 |
| | β2 | 0.334 ± 0.0868 | 0.3 ± 0.096 | 4.4 | 0.038 | 0.91 | 0.53 | -0.273 | 0.98 | -7.6 | 0.56 |
| | β4 | 0.33 ± 0.0948 | 0.28 ± 0.096 | 7.3 | 0.0079 | 0.747 | 0.69 | 4.87 | 0.66 | -8.5 | 0.51 |
| | γ1 | 0.328 ± 0.105 | 0.28 ± 0.099 | 6.24 | 0.014 | 0.744 | 0.69 | 8.98 | 0.42 | -1.8 | 0.89 |
| | γ2 | 0.318 ± 0.107 | 0.27 ± 0.096 | 6.54 | 0.012 | 0.636 | 0.8 | 9.14 | 0.41 | 3.2 | 0.8 |
| R Cent - L Temp | α2 | 0.391 ± 0.0914 | 0.36 ± 0.1 | 4.68 | 0.032 | 1.15 | 0.33 | 1.85 | 0.87 | -24 | 0.065 |
| | β1 | 0.395 ± 0.0822 | 0.36 ± 0.098 | 7.44 | 0.0074 | 1.56 | 0.12 | 3.46 | 0.75 | -18 | 0.16 |
| | β2 | 0.387 ± 0.0892 | 0.36 ± 0.1 | 4.46 | 0.037 | 1.65 | 0.095 | 1.76 | 0.87 | -14 | 0.27 |
| R Cent - L Par/Occ | α2 | 0.385 ± 0.0939 | 0.34 ± 0.12 | 6.17 | 0.014 | 1.45 | 0.16 | -9.42 | 0.39 | -21 | 0.095 |
| | β1 | 0.391 ± 0.0881 | 0.34 ± 0.12 | 7.34 | 0.0077 | 1.33 | 0.22 | -1.78 | 0.87 | -19 | 0.14 |
| | β2 | 0.391 ± 0.0938 | 0.34 ± 0.12 | 6.65 | 0.011 | 1.2 | 0.29 | -1.73 | 0.88 | -13 | 0.31 |
| | β3 | 0.374 ± 0.0986 | 0.33 ± 0.13 | 4.85 | 0.03 | 1.39 | 0.19 | -4.59 | 0.68 | -17 | 0.19 |
| | β4 | 0.374 ± 0.105 | 0.33 ± 0.13 | 4.82 | 0.03 | 1.24 | 0.27 | -1.8 | 0.87 | -15 | 0.25 |
| R Temp - L Par/Occ | β4 | 0.335 ± 0.0935 | 0.3 ± 0.1 | 4.43 | 0.037 | 0.991 | 0.46 | -8.84 | 0.42 | -10 | 0.44 |

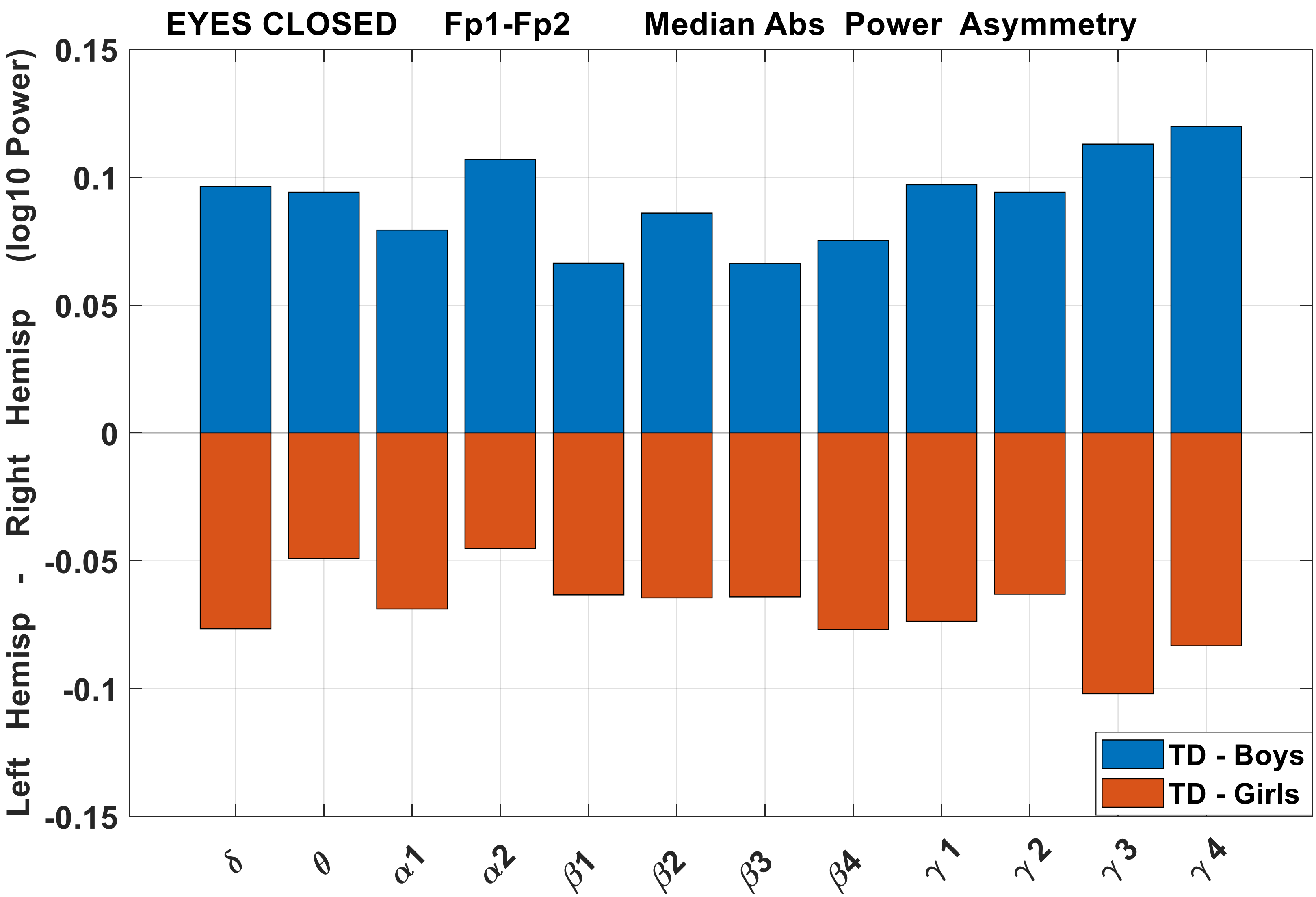


**Figure S1**. Comparison of median absolute power asymmetry at the frontal site (Fp1 – Fp2) between boys and girls

**Figure S-2:**Difference inf median absolute Spectral Power (log10) in 3 age bins for the EEG channels and δ, θ, α1, α2, β1, and β2 frequency bands.

Bin 1: 7-9 Years; n: Boys=37, Girls=27; Bin 2: 10-12 Years n: Boys=17, Girls=11;

Bin 3: 13-16 Years n: Boys=14, Girls=13; * indicated sig at $p < .05$

The figures are in a heat map style with a colormap that shows the hotter colors (brown and yellow) correspond to a positive value for the difference of girls minus boys (i.e., girls > boys), and the light green and blue (cooler colors) correspond to a negative value for the difference girls minus boys (i.e., boys > girls).

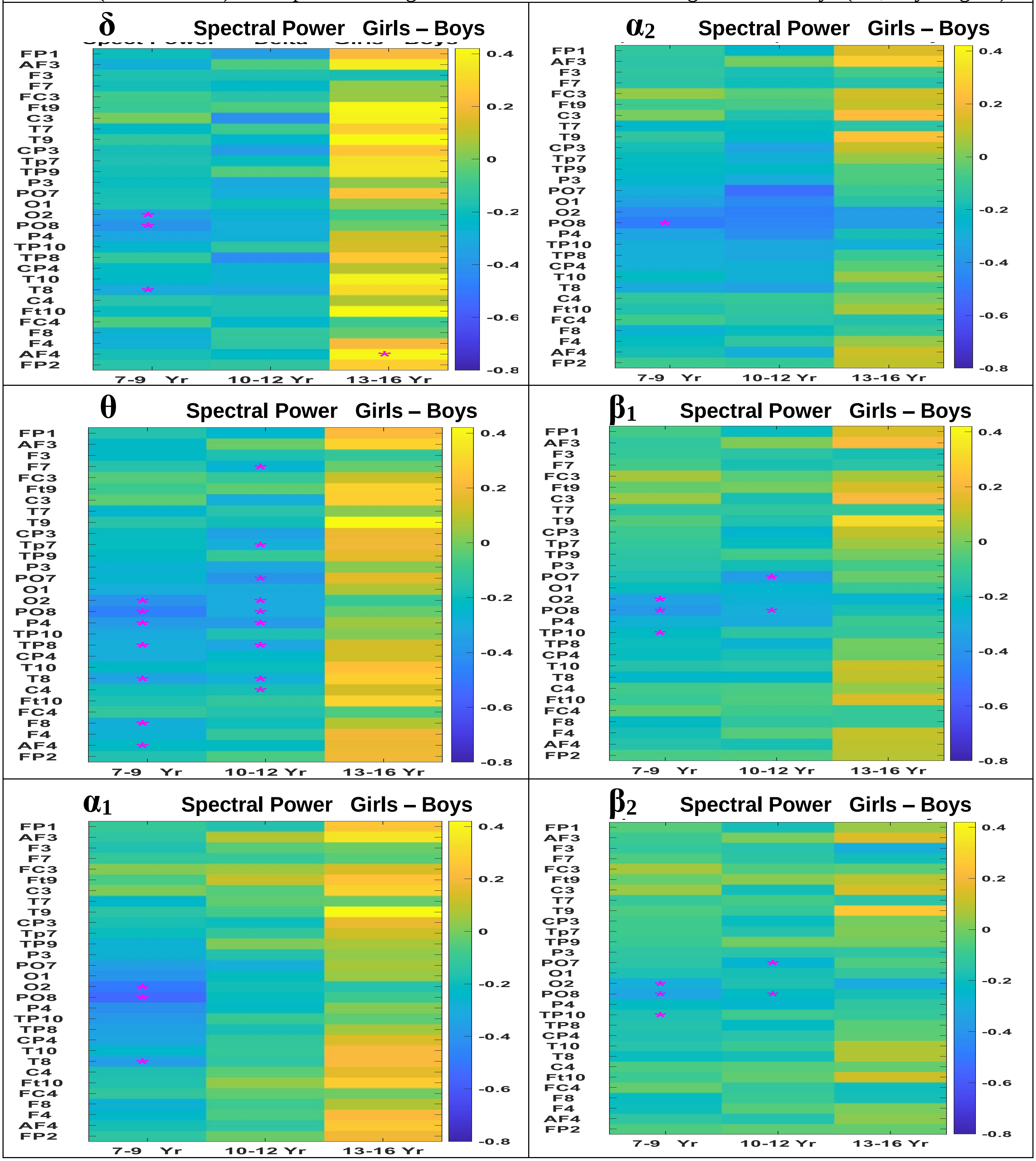

Supplement: Supplementary file 1 [file Data_Sheet_1.PDF]
